# Metabolic profiling of MRI‐measured liver fat in the UK Biobank

**DOI:** 10.1002/oby.23687

**Published:** 2023-03-05

**Authors:** Louisa Gnatiuc Friedrichs, Eirini Trichia, Diego Aguilar‐Ramirez, David Preiss

**Affiliations:** ^1^ Clinical Trial Service Unit and Epidemiological Studies Unit (CTSU), Nuffield Department of Population Health University of Oxford Oxford UK; ^2^ MRC Population Health Research Unit, Nuffield Department of Population Health University of Oxford Oxford UK

## Abstract

**Objective:**

Liver fat associates with obesity‐related metabolic disturbances and may precede incident diseases. Metabolomic profiles of liver fat in the UK Biobank were investigated.

**Methods:**

Regression models assessed the associations between 180 metabolites and proton density liver fat fraction (PDFF) measured 5 years later through magnetic resonance imaging, as the difference (in SD units) of each log metabolite measure with 1‐SD higher PDFF among those without chronic disease and not taking statins, and by diabetes and cardiovascular diseases.

**Results:**

After accounting for confounders, multiple metabolites were associated positively with liver fat (*p* < 0.0001 for 152 traits), particularly extremely large and very large lipoprotein particle concentrations, very low‐density lipoprotein triglycerides, small high‐density lipoprotein particles, glycoprotein acetyls, monounsaturated and saturated fatty acids, and amino acids. Extremely large and large high‐density lipoprotein concentrations had strong inverse associations with liver fat. Associations were broadly comparable among those with versus without vascular metabolic conditions, although negative, rather than positive, associations were observed between intermediate‐density and large low‐density lipoprotein particles among those with BMI ≥25 kg/m^2^, diabetes, or cardiovascular diseases. Metabolite principal components showed a 15% significant improvement in risk prediction for PDFF relative to BMI, which was twice as great (but nonsignificant) compared with conventional high‐density lipoprotein cholesterol and triglycerides.

**Conclusions:**

Hazardous metabolomic profiles are associated with ectopic hepatic fat and are relevant to risk of vascular‐metabolic disease.

**Study Importance**

**What is already known?**

- Metabolite‐profiling studies often focus on general adiposity, diagnosed hepatic steatosis, and major vascular diseases.
- No studies, to our knowledge, have assessed the metabolomic profiles of magnetic resonance imaging–measured liver fat content in otherwise healthy general populations and whether such profiles differ by presence of diabetes.

**What does this study add?**

- Lipoprotein particle sizes and concentrations were more hazardous for high liver fat content than total lipids or total low‐density lipoprotein cholesterol, particularly concentrations of extremely large, very large, and small high‐density lipoprotein particles and very low‐density lipoprotein triglycerides.
- Glycoprotein acetyls, monounsaturated and saturated fatty acids, and amino acids were also associated strongly with higher liver fat content.
- Among those with BMI >25 kg/m^2^, diabetes mellitus, or cardiovascular diseases, intermediate‐density lipoprotein and large low‐density lipoprotein particles were associated negatively with liver fat.

**How might these results change the direction of research?**

- The findings might potentially relate to causal insights, and these could be explored in further genetic and interventional studies.
- Further studies could assess the potential translational relevance of nuclear magnetic resonance‐measured biomarkers to liver fat content and cardiometabolic risk in other populations.

## INTRODUCTION

Excess adiposity is associated with ectopic fat depots, including accumulation of fat in the liver, which in turn is associated with increased risk of dysglycemia, diabetes [1], and cardiovascular diseases (CVD) [2]. Mendelian randomization studies have shown causal links between increased adiposity and multiple changes across the metabolite profile, both within the normal weight range and with changes in weight over time [3]. Furthermore, detailed profiling of circulating metabolites has shown that increased concentrations of very large particles of very low‐density lipoproteins (VLDL), as well as increased levels of triglycerides, unsaturated fatty acids (FAs), and branched‐chain amino acids, precede a diagnosis of hepatic steatosis [4], and they are also associated with the onset of hypertension [5] and incident cardiovascular events [6], independent of waist circumference, physical activity, and smoking. However, although the advent of novel assays such as high‐throughput nuclear magnetic resonance (NMR) metabolomic profiling now enables assessing adiposity‐associated hazards beyond traditional lipids, the objective assessment of hepatic steatosis or accumulation of fat in the liver in a general population (which is most likely to benefit from preventive interventions) is limited by the need for hepatic imaging by ultrasonography or other modalities because of the silent systemic or parenchymal changes that precede a clinical diagnosis. The UK Biobank provides a unique platform to objectively assess the metabolomic signatures of levels of hepatic fat, through available direct measures of 180 circulating metabolites and specialist readings of magnetic resonance imaging (MRI) of the liver [7]. We used the UK Biobank data to comprehensively assess the patterns of the associations among multiple metabolites and the 5‐year levels of liver fat.

## METHODS

The UK Biobank has been previously described (www.ukbiobank.com) [7]. Here we describe the data relevant to this report.

### Baseline and follow‐up assessment

Between 2006 and 2010, 502,493 men and women aged 40 years and older living in the UK were enrolled in a prospective study. Between 2014 and 2017 (approximately 5 years after the baseline UK Biobank assessments), 37,905 participants underwent detailed MRI body scans, including assessment of abdominal composition, using Advanced MR Analytics AB (AMRA Sweden). Liver fat was quantified in a subset of 4615 participants (at the time this analysis was conducted) using a 10‐point symmetric chemical shift encoded acquisition (10P) liver proton density fat fraction (PDFF) measured as the average PDFF in up to nine (and at least three) regions of interest in the liver [8]. These regions of interest avoid inhomogeneities, major vessels, and bile ducts, and values were derived by medical professionals based on the gradient echo imaging protocol. Among the participants with quantified liver fat, a subset of 1193 participants also had information on 180 metabolites quantified through NMR spectrometry on nonfasted blood samples collected at baseline, including direct measures of lipid subclasses and concentrations, FAs, amino acids, and markers of glycolysis (e.g., glucose, lactate, pyruvate), inflammation (e.g., glycoprotein acetyls), and renal function (e.g., creatinine, albumin), as well as an additional 69 derived ratios (details of subgroup classifications in Supporting Information Table S1) [9]. Values below the detection limit were assigned the value of the lower detection limit. Repeat measurements of the NMR metabolites were quantified in the blood collected at the first resurvey (2012–2013) in a sample of 5138 participants and they showed good reproducibility over time for most lipid subclasses and particle concentrations (correlation coefficients >0.5) and somewhat lower reproducibility for amino acids, glycolysis‐related metabolites, and ketone bodies (correlation coefficients <0.4) [10].

Among all 1193 participants with complete information on liver fat and NMR metabolites of interest for the current analysis, 1088 men and women had complete information on sociodemographic characteristics (e.g., Townsend index for deprivation, ethnicity), medical history (e.g., diagnosed disease, medication use), physical and biological assessments, lifestyle (e.g., smoking, alcohol consumption, physical activity habits), anthropometric measurements (e.g., height, weight), and biochemistry assays (e.g., glycated hemoglobin [hemoglobin A_1c_]); see footnote of Table 1 for credible values). Measurements of body composition (e.g., total fat and lean mass) were assessed using Tanita BC418MA bioelectrical impedance scales, as a function of age, sex, height, and weight. Fat mass index (FMI) and lean mass index (LMI) were calculated analogous to body mass index (BMI), so that bioimpedance direct estimates from fat mass (for FMI) and lean mass (for LMI) in kilograms were divided by height in meters squared (kg/m^2^).

**TABLE 1:** Characteristics of men and women aged 40–70 years at baseline

	Men (*n* = 517)		Women (*n* = 571)		All (*N* = 1088)	
Age (y), mean (SD)	56	7.7	55	7.7	56	7.7
<55 years, *n* (%)	203	39%	257	45%	460	42%
55+ years, *n* (%)	314	61%	314	55%	628	58%
Ethnicity, *n* (%)						
White	498	96%	559	98%	1057	97%
Smoking, *n* (%)						
Never	284	55%	355	62%	639	59%
Previous	208	40%	191	33%	399	37%
Current	25	5%	25	4%	50	5%
Drinking, *n* (%)						
Never	25	5%	36	6%	61	6%
Previous	72	14%	137	24%	209	19%
Current	420	81%	398	70%	818	75%
Biological measures^a^						
BMI (kg/m^2^), mean (SD)	27.2	3.8	26.3	4.6	26.7	4.3
FMI (kg/m^2^), mean (SD)	6.8	2.4	9.7	3.4	8.3	3.3
LMI (kg/m^2^), mean (SD)	20.3	1.8	16.6	1.5	18.4	2.5
Liver fat (PDFF), median (IQR)	2.6	1.6−5.2	1.9	1.2−4.2	2.2	1.3−4.7
Total cholesterol (mmol/L), mean (SD)	4.4	0.9	4.8	0.8	4.6	0.9
LDL‐c (mmol/L), mean (SD)	1.7	0.4	1.8	0.4	1.7	0.4
HDL‐c (mmol/L), mean (SD)	1.2	0.3	1.4	0.3	1.3	0.3
Total triglycerides (mmol/L), mean (SD)	1.4	0.6	1.2	0.5	1.3	0.5
Total esterified cholesterol (mmol/L), mean (SD)	3.2	0.6	3.5	0.6	3.4	0.6
Total free cholesterol (mmol/L), mean (SD)	1.2	0.2	1.3	0.2	1.3	0.2
Phosphoglycerides (mmol/L), mean (SD)	2.1	0.4	2.4	0.3	2.3	0.4
Apolipoprotein B (g/L), mean (SD)	0.8	0.2	0.9	0.2	0.9	0.2
Apolipoprotein A1 (g/L), mean (SD)	1.3	0.2	1.5	0.2	1.4	0.2
Total fatty acids (mmol/L), mean (SD)	11.6	2.4	12.0	2.1	11.8	2.2
Glucose (mmol/L), mean (SD)	3.4	0.7	3.5	0.7	3.5	0.7
Creatinine (mmol/L), mean (SD)	0.1	0.01	0.1	0.01	0.1	0.01
Albumin (mmol/L), mean (SD)	39.3	3.0	39.1	3.0	39.2	3.0
Glycoprotein acetyls (mmol/L), mean (SD)	0.8	0.1	0.8	0.1	0.8	0.1
Medical histories^b^, *n* (%)						
BMI ≥25 kg/m^2^	366	71%	311	54%	677	62%
Any chronic disease^c^	255	49%	231	40%	486	45%
Diabetes^d^	52	10%	49	9%	101	9%
Vascular disease^e^	224	43%	175	31%	399	37%
Lipid‐lowering medication^f^	106	20%	58	10%	164	15%

Abbreviations: FMI, fat mass index; HDL‐c, high‐density lipoprotein cholesterol; LDL‐c, low‐density lipoprotein cholesterol; LMI, lean mass index; PDFF, MRI‐measured proton density liver fat fraction.

^a^ All values represent absolute measures. Excluded: implausible values (height <120/>200 cm, weight <35/>250 kg, BMI <15/>60 kg/m^2^, metabolite measures </> 4 SD) and incomplete information.

^b^ Known diagnoses at the time of the first assessment.

^c^ Hospital ICD‐coded diagnoses of any vascular, diabetic, hepatic, renal, neoplastic, tuberculosis, or chronic respiratory diseases at baseline.

^d^ Self‐reported or hospital diagnoses (ICD‐10: E10–E16) or hemoglobin A_1c_ ≥48 mmol/L.

^e^ Hospital diagnoses (ICD‐10: I01–I99).

^f^ Self‐reported.

Self‐reported medical histories and linkage to the hospital episode statistics (HES) provided information on nonfatal events accrued between March 1992 and April 2021 (or March 2018 for participants from Wales), and they were coded according to the *International Classification of Diseases, Tenth Revision* (ICD‐10) [11]. The presence of diabetes at baseline assessment was defined by self‐reported medical diagnosis, any mention of HES‐reported ICD‐10 codes E10 to E16, or levels of measured hemoglobin A_1c_ ≥48 mmol/L (in the absence of a diagnosis). The presence of vascular disease was defined by any mention of the ICD‐10 codes I01 to I99 in the HES records. In addition to diabetes and vascular diseases, major chronic diseases also included HES‐reported diagnoses generally associated with adiposity, specifically chronic kidney disease (ICD‐10 codes N18–N19, N039, N049, N059, N12, N142, N281, N289, E102, E112, Q612, Q619, Y841, I120), hepatic diseases (ICD‐10 codes K70–K77, D134, I85, I864, I982–I983), neoplasms (ICD‐10 codes C00–D9, D37–D50), and major respiratory diseases (ICD‐10 codes A15–A19, B90, J40–J47).

The present analysis was conducted under the UK Biobank approved project number 70682, and the application was funded by the Nuffield Department of Population Health at the University of Oxford. UK Biobank has approval from the North West Multi‐center Research Ethics Committee (MREC) as a Research Tissue Bank (RTB) approval (Reference 21/NW/0157, the Integrated Research Application System [IRAS] Project ID 299116). All study participants provided written informed consent.

### Statistical analyses

Participants who had extreme values for anthropometry measures (height <120 cm or >200 cm, weight <35 kg or >250 kg, BMI <15 kg/m^2^ or >60 kg/m^2^), had implausible NMR measurements (outside 4 standard deviations [SDs]), or had missing values on any covariates were excluded. The sex‐specific characteristics of the subset with complete information of interest at baseline were described by univariate analyses. Sociodemographic, lifestyle, and medical characteristics, as well as levels of liver fat and NMR metabolites, were summarized as percentages, means with SDs, or medians with interquartile ranges (IQRs), as appropriate. The distributions of liver fat measures and of the major classes of NMR metabolites were histogrammed, and (because of skewness) NMR values (only) were log‐normalized (e.g., subtraction of the mean and division by the SD; Supporting Information Figure S1). Spearman correlation coefficients between direct measures of the NMR metabolites were mapped (Supporting Information Figure S2).

Linear regression first assessed the shapes of the associations of the estimated mean levels and 95% confidence intervals (CIs) of liver fat concentrations across quintiles of the distribution of indices of body mass (BMI, FMI, LMI) and across log‐values of major classes of NMR metabolites (total lipids in lipoproteins, high‐ and low‐density cholesterol, total triglycerides, apolipoprotein A [ApoA] and apolipoprotein B [ApoB], total FAs, phospholipids, cholesterol classes, specific amino acids, glucose, creatinine, ketone bodies, and total sizes and concentrations of VLDL, intermediate‐density lipoprotein [IDL], low‐density lipoprotein [LDL] cholesterol, and high‐density lipoprotein [HDL] particles). All analyses were adjusted for confounders (age, sex, quintiles of the Townsend index for deprivation, self‐reported ethnicity [British, Asian, Afro‐Caribbean, other], smoking and alcohol consumption habits [never, former, current]), spectrometer, and the position of the biological sample on the magnetic plaque. Estimates were displayed against the mean levels of each metabolite across 2 SDs of each marker (e.g., for visual comparison of shapes of the association).

Subsequently, the overall strength of the association between each NMR metabolite (and derived ratios) at baseline (e.g., exposure) and the 5‐year level of liver PDFF (e.g., outcome) was further estimated with each log‐normalized NMR marker and liver fat content, considered as a continuous increase per 1‐SD higher level across the ranges. The beta coefficients and corresponding 95% CIs were circumferentially plotted according to metabolic groups. To account for multiplicity, the false discovery rate was controlled at 5% using the Benjamini–Hochberg method [12]. Thus, for each association, a false discovery rate–adjusted *p* value < 0.05 was considered as evidence against the null hypothesis. Analyses were conducted among all eligible participants and separately among those without and with diabetes, without and with vascular diseases, and without and with known major chronic disease. Those taking lipid‐lowering medications were further excluded from the groups “without” disease. Additional analyses included estimates by age <55/≥55 years, sex, BMI <25 kg/m^2^/≥25 kg/m^2^, and smoking status (never/ever). The absolute differences in the strengths of the associations between the various subgroups were calculated as the difference in beta coefficients. Finally, to assess whether circulating NMR metabolites could improve prediction of liver fat concentrations, uncorrelated selected principal components (PCs), which retained 90% of the variance of the individual biomarkers, were added to conventional predictive models including BMI and biochemistry‐measured HDL cholesterol, triglycerides, alanine transaminase, and diagnosed diabetes. The overall predictive ability of each model was assessed by the bias‐adjusted *R* ^2^ statistic calculated with bootstrapping using 100 replications. *R* ^2^ quantifies the proportion of variance in the outcome explained by the predictors, whereas the Χ^2^ statistic shows improvement in predictability given progressive account for various predictors relative to the basic model. All statistical analyses were performed using Stata version 17 (StataCorp LLC), and plots were created in R version 4.0.5 (www.r-project.org) with the package RCircos [13].

## RESULTS

### General characteristics

**FIGURE 1:**
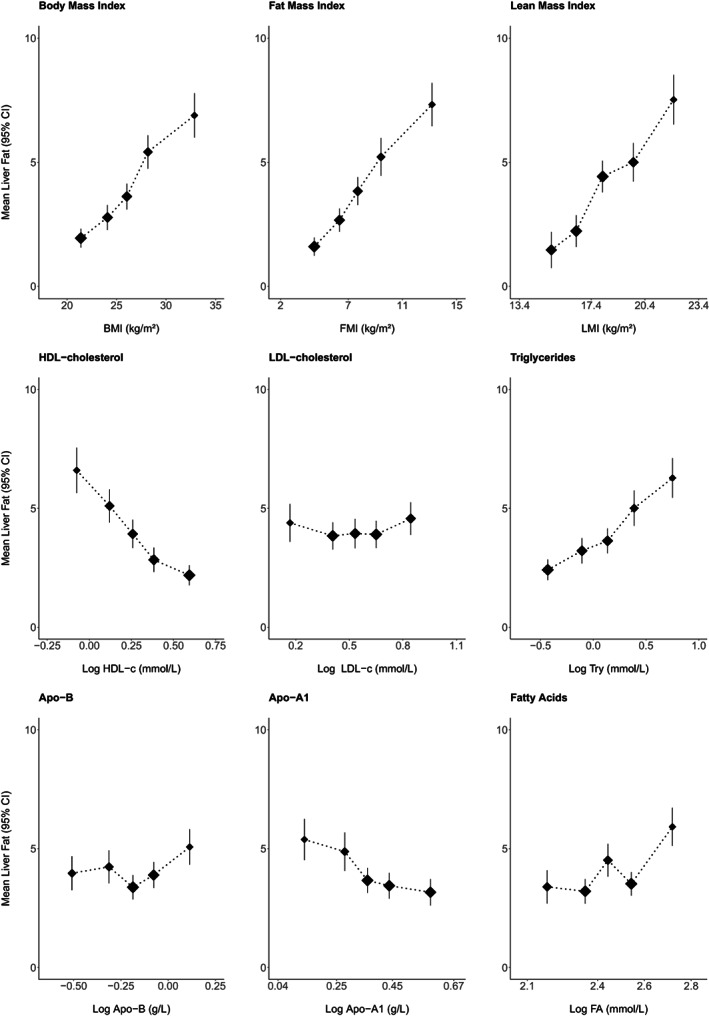
Levels of MRI‐measured proton density liver fat fraction by baseline levels of adiposity and selected NMR‐measured lipids. Estimates were calculated with regression models adjusted for confounders (age, sex, quintiles of the Townsend index, ethnicity, smoking and drinking status, NMR spectrometer, and the position of the blood sample in the magnetic plate) within five equally sized groups of the sex‐specific distributions of each measure at baseline. NMR metabolite values were log‐normalized. Y‐axes extend 1 SD above and below the mean of each marker. Square sizes are inversely proportional to the variance. Exclusions and conventions as per Table 1. Apo, apolipoprotein; FA, fatty acids; FMI, fat mass index; HDL‐c, high‐density lipoprotein cholesterol; LDL‐c, low‐density lipoprotein cholesterol; LMI, lean mass index; Try, triglycerides

Among 1088 participants with complete information (52% women, mean age 56.5 [SD 7.7] years), 59% were never smokers, 6% were never drinkers, 9% had diagnosed or screen‐detected diabetes, 37% had any type of vascular disease, and 45% had any type of chronic disease (Table 1). Mean (SD) BMI at baseline was 27.2 (3.8) kg/m^2^ in men and 26.3 (4.6) kg/m^2^ in women, 62% of all participants had BMI ≥ 25, and median liver fat concentration (5 years after baseline) was 2.6% (IQR 1.6%–5.2%) in men and 1.9% (IQR 1.2%–4.2%) in women. Baseline mean (SD) levels of NMR‐measured total cholesterol, triglycerides, and FAs were 4.6  (0.9), 1.3 (0.5), and 11.8 (2.2) mmol/L, respectively; ApoA1 and ApoB 1.4 (0.2) and 0.9 (0.2) g/L, respectively; glucose 3.5 (0.7) mmol/L; creatinine 0.1 (0.01) mmol/L; and glycoprotein acetyls 0.8 (0.1) mmol/L. (For comparison, the characteristics of various subgroups with and without NMR metabolites and MRI profiling are given in Supporting Information Table S4.) Across all metabolites, the mean levels of most measures were discretely higher in women compared with men (Supporting Information Table S1), and values were intercorrelated across the spectrum (Supporting Information Figure S2). Levels of liver fat increased linearly with increased levels of baseline BMI, FMI, and LMI (Figure 1).

### Lipids and metabolites versus liver fat concentrations

Levels of liver fat increased linearly with higher levels of triglycerides, small HDL cholesterol particles, creatinine, amino acids, and glycoprotein acetyls (Figure 1). Liver fat content was somewhat more modestly elevated with higher baseline levels of LDL cholesterol, ApoB, and total FAs, and it appeared inversely associated with baseline levels of total HDL cholesterol, extra‐large and large HDL particle concentrations, ApoA1, glycine, and glutamine (Supporting Information Figure S3).

**FIGURE 2:**
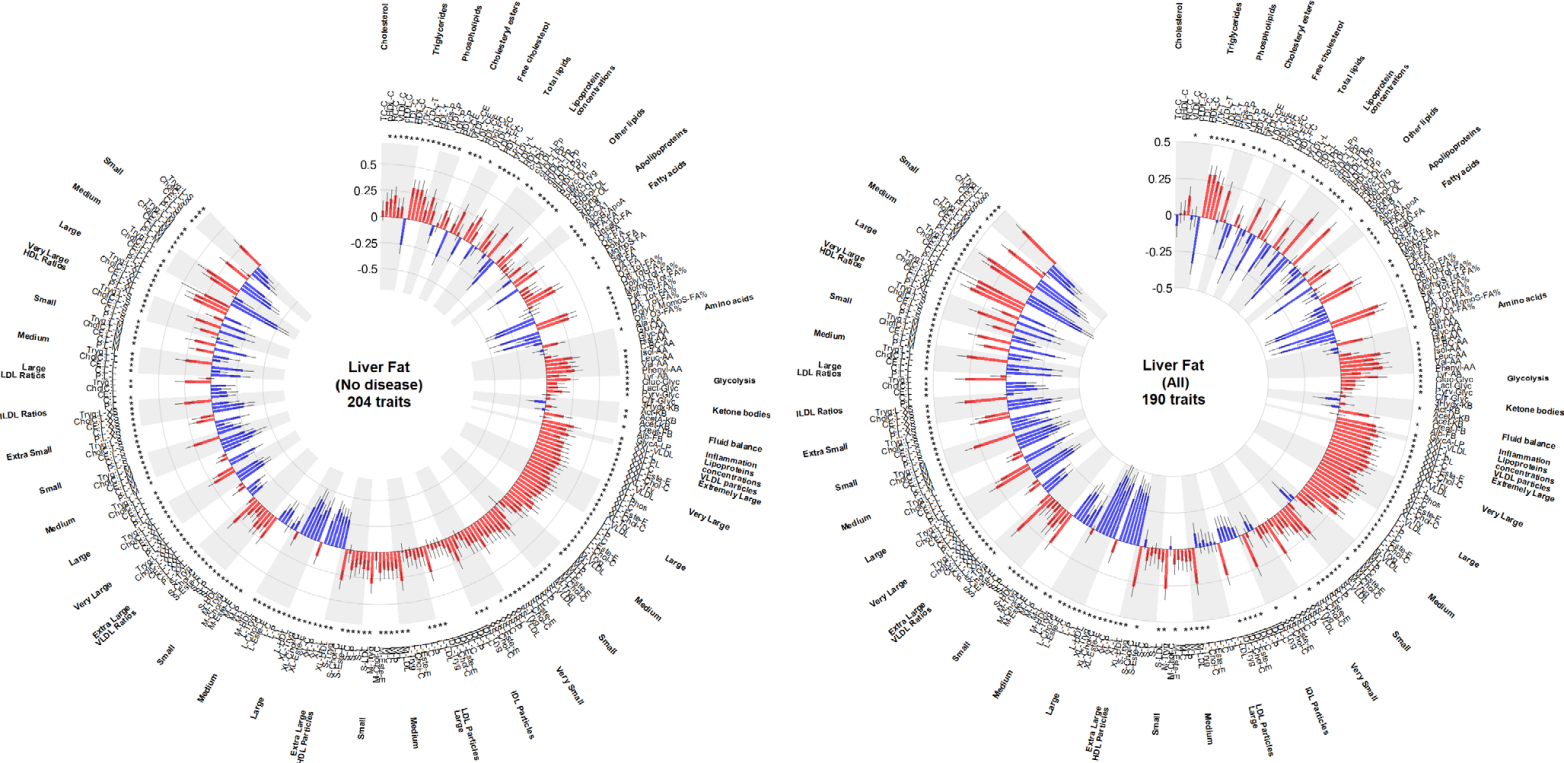
NMR metabolites and liver fat. Regression models estimated the beta coefficients and 95% CIs for 1‐SD increase in log‐levels of each NMR metabolite and their ratios at baseline assessment, with 1‐SD higher level of MRI‐measured proton density fat fraction in the liver 5 years later, adjusted for confounders (as per Figure 1). Blue bars show negative associations, red bars show positive associations. The black vertical lines through the bars represent the 95% CIs. Starred estimates represent *p* < 0.05 for Benjamini–Hochberg adjusted false discovery rate, and their total count (traits) is shown in the middle of the graph. Exclusions as per footnote of Table 1. Those without chronic disease excludes self‐reported or hospital‐recorded medical histories (defined as per footnote of Table 1) and those taking lipid‐lowering medication at baseline. HDL, high density lipoproteins; IDL, intermediate‐density lipoproteins; LDL, low‐density lipoproteins; VLDL, very low density lipoproteins [Color figure can be viewed at wileyonlinelibrary.com]

Figure 2 shows the lipid and metabolomic profiles associated with levels of liver fat 5 years later, separately in those without versus with known chronic diseases (expressed as strengths of the associations estimated by adjusted beta coefficients of mean differences per 1‐SD higher liver fat concentration, equivalent to an average 0.85% higher level, for a given difference per 1‐SD higher log‐NMR biomarker). Associations varied by lipoprotein subclass (i.e., VLDL, IDL, LDL, and HDL particles) and size (extremely large, extra‐large, medium, small, and extra‐small), by apolipoprotein and lipid subclass (i.e., ApoA1; ApoB; number of lipoprotein particles; and total, free, or esterified cholesterol, triglycerides, phospholipids, and total lipids), and by NMR biomarkers of glycolysis (e.g., glucose), fluid balance (e.g., creatinine), and inflammation (e.g., glycoprotein acetyls). More detailed analyses are provided in the following sections.

### ApoB‐carrying lipoproteins and their lipids

Higher levels of triglycerides at baseline were more strongly associated with higher levels of liver fat 5 years later (beta coefficients > 0.30, *p* < 0.00001) than phospholipids and total cholesterol (beta coefficients of 0.10–0.25, *p* ≥ 0.01; Figure 2; Supporting Information Table S2). Consequently, most lipid subtypes, considered as a ratio relative to total lipids, appeared inversely associated with higher liver fat. The concentrations of VLDL particles were more strongly associated with higher levels of liver fat than concentrations of LDL particles, but the strength of the association weakened with decreasing size of VLDL particles. Higher triglycerides in all lipoproteins (VLDL, IDL, LDL, HDL particles) were also associated with higher levels of liver fat, including when considered relative to total lipids. Consequently, higher ApoB and larger VLDL diameters at baseline were associated with higher liver fat 5 years later, and the associations were stronger, particularly among those without chronic disease and not taking lipid‐lowering treatment at the time the NMR metabolites were measured (beta coefficients 0.15 [*p* = 0.001] and 0.29 [*p* < 0.00001], respectively).

### HDL‐related biomarkers

The relevance of HDL biomarkers to liver fat concentrations varied by lipoprotein subclass and concentration. Associations were generally inverse for very large and large particle concentrations (Supporting Information Figure S3) but positive for small HDL particles, irrespective of the lipid type (phospholipids, total or free cholesterol, cholesteryl esters; Figure 2; Supporting Information Table S2). By comparison, higher levels of triglycerides within HDL particles were positively related to liver fat, irrespective of the size of the HDL particles (Supporting Information Table S2). Considering all HDL subclasses together, slightly lower ApoA1 levels and lower HDL diameters at baseline were associated with higher liver fat 5 years later, and these associations were consistent irrespective of known histories of chronic diseases (beta coefficients −0.18, [*p* < 0.00001] and −0.37 [*p* < 0.00001], respectively; Supporting Information Table S2).

### Fatty acids

Higher levels of total FAs were associated with higher levels of liver fat, with the strongest positive associations observed for monounsaturated FAs (MUFAs) and saturated FAs (SFAs) (beta coefficients >0.23, *p* < 0.00001; Supporting Information Table S2). By contrast, relative to total FAs, higher levels of omega‐3 and omega‐6, linoleic, and polyunsaturated FAs (e.g., ratios) were negatively associated with liver fat concentrations (beta coefficients <−0.07, *p* < 0.05; Supporting Information Table S2).

### Glycolysis metabolites, amino acids, ketone bodies, fluid balance, and inflammation

Higher levels of creatinine, isoleucine, leucine, valine, tyrosine, and Glyc‐A were positively associated with higher levels of liver fat (beta coefficients >0.21, *p* < 0.0001), and higher levels of phenylalanine, glucose, lactate, and pyruvate were modestly associated with liver fat (beta coefficients >0.08, *p* < 0.004; Figure 2; Supporting Information Table S2). By contrast, higher levels of acetate, acetone, glutamine, and glycine were inversely associated with levels of liver fat (beta coefficients <−0.03, *p* = 0.02 to 0.2).

### Associations by age, sex, and smoking

The patterns of the associations between levels of NMR metabolites and liver fat concentrations were broadly comparable irrespective of sex and smoking status (Supporting Information Figure S4). On average, associations were discretely stronger in women compared with men across the full spectrum of the metabolites, except for very small VLDL and extra‐large and large HDL particle concentrations (Supporting Information Table S3). By contrast, associations were somewhat heterogeneous in younger versus older ages, because the inverse associations for FAs, IDL particles, and large and medium LDL particles and the positive associations for amino acids with liver fat 5 years later appeared somewhat stronger among those older than 55 years (Supporting Information Figure S4 and  Table S3). Moreover, associations of baseline NMR metabolites with liver fat concentrations 5 years later were discretely stronger among never smokers compared with ever smokers, except for total HDL cholesterol (irrespective of lipid type), total particle concentration, medium and small HDL concentrations, ApoA1, triglycerides, FA subtypes, alanine, histidine, pyruvate, creatinine, citrate, and ketone bodies, which were discreetly more strongly associated with liver fat among smokers (difference in beta coefficients >−0.02; Supporting Information Table S3).

### Associations by level of obesity

**FIGURE 3:**
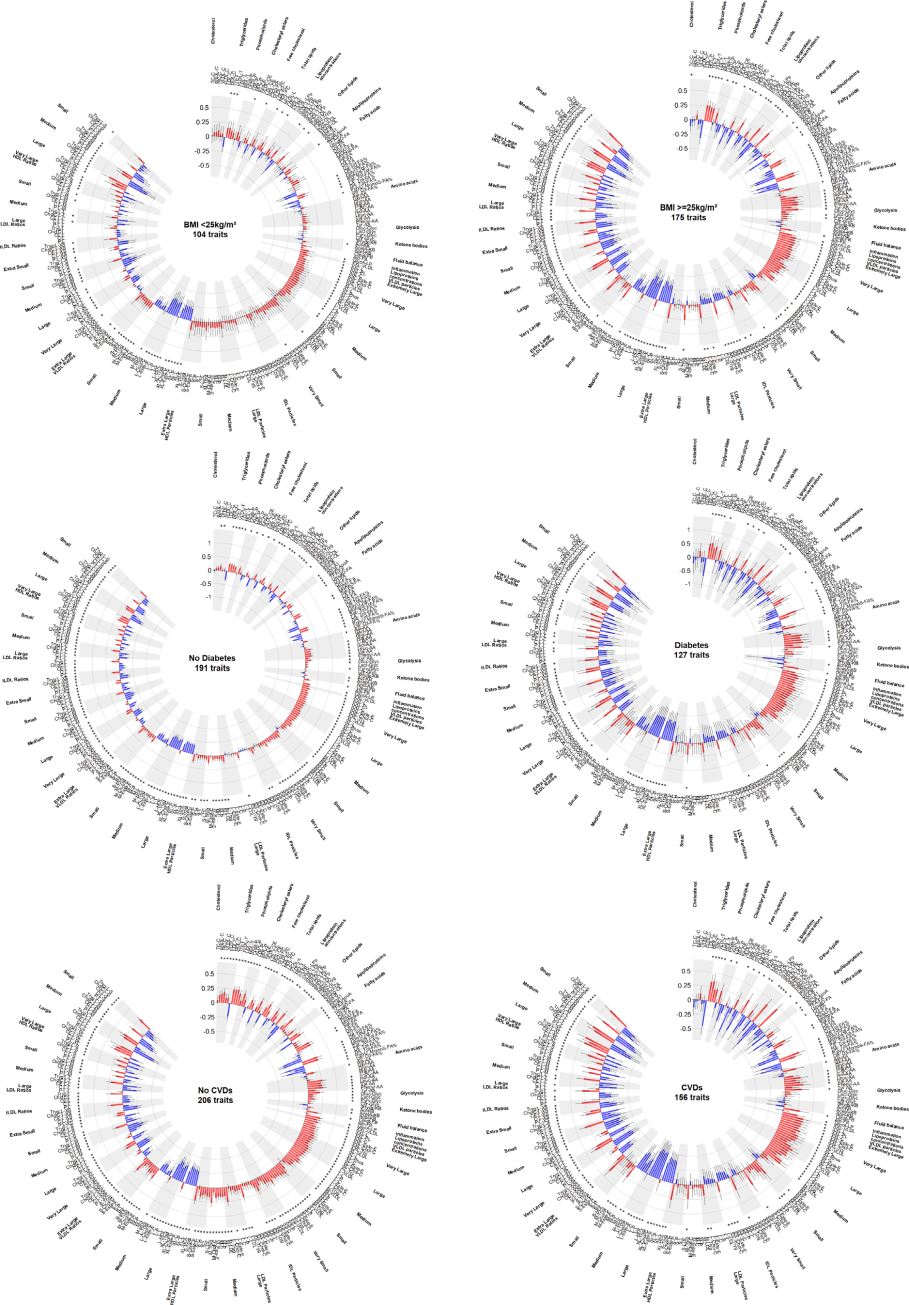
NMR metabolites and liver fat, by levels of BMI, diabetes status, and cardiovascular diseases (CVD). Analyses as per Figure 2. Diabetes was defined as per footnote to Table 1. The group without diabetes also excluded those who were taking lipid‐lowering medications at baseline. CVD defined as per footnote of Table 1. The group without CVD also excluded those who were taking lipid‐lowering medication at baseline. HDL, high‐density lipoproteins; IDL, intermediate‐density lipoproteins; LDL, low‐density lipoproteins; VLDL, very low density lipoproteins [Color figure can be viewed at wileyonlinelibrary.com]

The patterns of the associations between baseline levels of NMR metabolites with liver fat concentrations 5 years later were broadly consistent irrespective of the level of BMI at baseline, except for IDL particles, which were not associated with liver fat among those with BMI < 25 who were not taking lipid‐lowering medications (Figure 3). Associations for triglycerides, large VLDL particles, concentrations of HDL particles, MUFAs, SFAs, glycolysis‐related metabolites, and amino acids were stronger among those with BMI ≥ 25 compared with < 25, whereas associations for total cholesterol and ApoB were stronger among those with BMI < 25 rather than BMI ≥ 25 (Figure 3; Supporting Information Table S3). Isolated opposite associations were observed for omega‐3 and omega‐6 FAs, polyunsaturated FAs, linoleic FAs, cholesteryl esters, and free cholesterol in small VLDL and large LDL particles, which appeared inversely associated with liver fat among those with BMI ≥ 25 rather than BMI < 25, and for lactate, pyruvate, and citrate, which appeared inversely associated with liver fat among those with BMI ≥ 25 rather than < 25 (Figure 3; Supporting Information Table S3).

### Associations by diabetes

The patterns of the associations between baseline levels of NMR metabolites and liver fat concentrations 5 years later were somewhat heterogeneous among those without diabetes not taking lipid‐lowering medications compared with those with diabetes, and they were about twice as strong among those with versus without diabetes (Figure 3). Among those with diabetes, triglycerides, concentrations of large VLDL particles (irrespective of size and lipid class), glucose and glycolysis‐related metabolites, MUFAs, SFAs, most amino acids, creatinine, glycoprotein acetyls, and lipoproteins were strongly positively associated with liver fat (beta coefficients of 0.30 to 0.70), whereas large lipoprotein concentrations in HDL particles were strongly inversely associated with liver fat (beta coefficients <−0.65; Supporting Information Table S3). By comparison, ApoA1, small VLDL particles, and lipoprotein concentrations in medium HDL particles were modestly associated with liver fat (beta coefficients of 0.20 to 0.40). Opposite associations were observed for total LDL cholesterol and concentrations of LDL particles, ApoB, remnant cholesterol, phospholipids, cholesteryl esters in LDL particles, and omega‐3 FAs, which appeared positively associated with liver fat among those without diabetes only (Figure 3; Supporting Information Table S3).

### Associations by cardiovascular and other chronic diseases

The patterns of the associations between baseline levels of NMR metabolites and liver fat concentrations 5 years later differed among those with versus without a history of CVD (Figure 3), although associations were largely comparable (both qualitatively and quantitatively) between those with diabetes versus with CVD and between those without diabetes versus without CVD. Further exclusion of any other chronic disease showed similar patterns, but much stronger associations, as described among those without diabetes or without CVD (Figure 1; Supporting Information Table S3). Across the full spectrum of metabolites studied, the associations with liver fat concentrations were more numerous among those who did not have any chronic disease at the time that the NMR metabolites were measured (*p* < 0.05 for 204 traits).

### Predictive value of the NMR metabolites

Conventional prediction models showed that baseline BMI, in addition to age, sex, smoking, and drinking, explained 13% (8%–19%) of the 5‐year variation in liver fat concentrations, and the addition of biochemistry‐measured HDL cholesterol, triglycerides, alanine transaminase, and a history of diabetes further explained 1% to 7% of the variation in liver fat concentrations (Supporting Information Table S5). By comparison, the addition of the first 10 PCs of the NMR metabolites (explaining 90% of the initial variation of all metabolites included; see Supporting Information Table S6 and footnote of Supporting Information Table S5 for details) significantly increased the predictive performance of the conventional model with BMI alone and explained an additional 15% of the variation in liver fat concentrations (changes in the *R* ^2^ statistic from 0.13 [ 0.08–0.19] to 0.28 [0.22–0.33]; Supporting Information Table S5). Similarly, when added to the full conventional models, PCs explained an additional 6% to 8% of the variation in liver fat (*R* ^2^ statistic of 0.30; 0.23–0.36); however, these additive improvements did not reach conventional significance.

## DISCUSSION

In this study of objectively measured concentrations of liver fat, detailed biomarker profiling showed multiple adverse characteristics with circulating lipids and metabolites. The patterns of these associations were associated with alterations in biomarkers linked to excess general adiposity [3, 4, 14, 15, 16], diabetes [17, 18], CVD [5, 6, 19, 20], and other major chronic diseases. Hazardous metabolic profiles linked to higher concentrations of fat in the liver involved higher levels of VLDL triglycerides, extremely large and very large lipoprotein concentrations, small HDL particles, glycolysis and inflammatory markers, MUFAs and SFAs, and amino acids. These unfavorable metabolic profiles were stronger among those with higher levels of general adiposity (compared with those with BMI <25 kg/m^2^) and among those with diagnosed or screen‐detected diabetes (compared with those without diabetes not taking lipid‐lowering medications). However, among those with excess BMI or diabetes, associations with higher total cholesterol, ApoB, and IDL and large LDL particles, which were otherwise hazardous among those without such metabolic conditions, appeared inverse. Such findings likely reflect residual effects from a greater usage of lipid‐lowering medications among individuals with diabetes and comorbid CVD, which are also more likely to be diagnosed among individuals with a high BMI. The observed qualitative and quantitative differences, both in adverse metabolic profiles with liver fat in general and between individuals with versus without major chronic disease, highlight opportunities for earlier identification and, potentially, prevention of nonalcoholic fatty liver disease (NAFLD). However, when added to the conventional predictors, NMR‐metabolite PCs achieved statistically insignificant improvements in liver fat concentration risk prediction, except when compared with BMI alone.

The present findings add to the previous evidence describing complex hazardous associations among body composition, metabolic disorders, NAFLD, diabetes, and CVD risk in the same population [14, 15], and they are in line with previous Mendelian randomization studies from Finnish or other populations, which have established causal links between excess adiposity and unfavorable metabolomic profiles [3, 6, 21], as well as consequent excess risk of hepatic steatosis [4], high blood pressure [5], and cardiovascular events [20, 21] (and also for some chronic respiratory diseases and neoplasms [21]). The mechanisms through which unfavorable lipid profiles increase risk from atherosclerosis and insulin dysregulation through excess lipid accumulation in the liver are complex but well established [2, 22]. Such unfavorable metabolic perturbations are more hazardous with excess adiposity in the visceral regions than with excess gluteo‐femoral adiposity or total body mass [23], and they likely reflect hazards from excess hepatic fat. Our findings add novel hypotheses about the relevance of different lipoprotein particle sizes and concentrations, complementary to the known hazards from total LDL and HDL cholesterol for mechanisms of NAFLD and vascular‐metabolic disease. The systemic consequences of high concentrations of adipocytokines, interleukins, small dense LDL particles, and nonesterified fatty acids and the reduced clearance of ApoB, triglycerides, and very low‐density lipids are related to impaired liver function, increased insulin resistance, glucose intolerance, and procoagulation factors [2]. Higher levels of VLDL, triglycerides, FAs, leucine, isoleucine, valine, glucose, and ketone bodies have been linked to incident insulin resistance and type 2 diabetes [17, 18, 24]. Moreover, detailed metabolic studies of clinical trials have highlighted that reductions in liver and pancreatic fat are a critical step toward the remission of type 2 diabetes [25]. Furthermore, our findings on the hazardous associations between elevated MUFAs and SFAs and concentrations of liver fat intersect with findings from meta‐analyses of multiethnic cohorts, showing that concentrations of selected fatty acids are involved in de novo lipogenesis and that they have been positively associated with incident diabetes [26]. Alterations in ApoB‐carrying VLDL, triglycerides, and inflammation markers link adiposity to a predisposition to atherogenic plaques, atherothrombotic CVD [27, 28], and coronary artery disease [29], yet potential mechanisms underlying the associations between unfavorable lipid profiles and kidney, respiratory, or neoplastic diseases are not fully understood.

To our knowledge, this is the first study to comprehensively assess the metabolomic profiles of liver fat from imaging. The study has several strengths and limitations. Liver fat concentration was estimated based on highly sensitive quantitative MRI techniques, and NMR metabolomics has been widely used as a novel platform toward precision medicine studies in general populations. However, interpreting observational metabolite associations remains complex and challenging [30], and we were unable to prospectively assess potential differences by incident (rather than prevalent) hepatic steatosis, diabetic, vascular, metabolic, or other relevant diseases, and the relevance of long‐term rather than baseline levels of metabolites is likely underestimated. Furthermore, the blood samples were collected in nonfasting conditions, resulting in likely greater variability in the metabolite concentrations than fasting measurements and likely a dilution of the estimates toward the null. Nonetheless, fasting duration has previously been shown to account for only a small proportion of variation in the concentrations of plasma metabolites [31]. In addition, although these observational findings are informative, causal associations by means of genetic instruments for the biomarkers of interest are needed to formally assess these preliminary findings. Such studies would likely overcome some of the potential collider bias that might be induced by the use of statins or stratification by levels of adiposity or preexisting cardiometabolic disease [32]. Furthermore, although this study is one of the largest of its kind, the limited number of study participants with complete information of interest cannot rule out some potential residual selection bias and the play of chance; however, the general characteristics of those with complete NMR measures and confounders data were comparable to the subset included in these analyses. Finally, the UK Biobank population is somewhat more healthy than the general UK population [33], and our data are not expected to be considered representative or to impact on the generalizability of our findings to the wider UK or other populations [34]. Therefore, comparable studies in other populations are warranted.

## CONCLUSION

In this UK population, hazardous metabolomic profiling was associated with higher concentrations of liver fat, including NMR measures of VLDL and LDL subtypes, glycolysis and inflammation biomarkers, and amino acids, whereas HDL particles showed inverse associations. These findings have potential translational applications for assessment of diabetic, cardiovascular, and other major chronic disease risk in otherwise healthy middle‐aged adults with mildly elevated levels of general adiposity, although the additional predictive value of the metabolites over conventional risk factors appears limited beyond BMI.

## AUTHOR CONTRIBUTIONS

Louisa Gnatiuc Friedrichs and David Preiss designed the study. Eirini Trichia provided data management and processing. Louisa Gnatiuc Friedrichs performed the analyses and wrote the first draft. Diego Aguilar‐Ramirez designed the graphic templates. All authors revised the article for intellectual content.

## FUNDING INFORMATION

The UK Biobank application was funded by the Nuffield Department of Population Health (NDPH) at the University of Oxford, Oxford, UK. Louisa Gnatiuc Friedrichs, Eirini Trichia, and David Preiss are funded by the MRC Population Health Research Unit, NDPH, University of Oxford. Diego Aguilar‐Ramirez is funded by a Junior Research Fellowship awarded by the NDPH at the University of Oxford.

## CONFLICT OF INTEREST

The authors declared no conflict of interest.

## Supporting information


**FIGURE S1:** Distributions of liver fat and selected NMR metabolites (log‐values).
**FIGURE S2:** Correlation map of direct measures of NMR metabolites.
**TABLE S1:** Sex‐specific levels of NMR metabolites.
**FIGURE S3:** Levels of MRI‐measured proton density liver fat fraction by baseline levels of total NMR‐measured lipids.
**FIGURE S4:** NMR metabolites and liver fat by age, sex, and smoking status.
**TABLE S2:** Associations of log‐levels of NMR metabolites (per 1 SD) with levels of liver fat (per 1 SD), by diabetes.
**TABLE S3:** Absolute differences in the associations of log‐levels of NMR metabolites (per 1 SD) with levels of liver fat (per 1 SD), by different characteristics at baseline.
**TABLE S4:** Baseline characteristics of study population subgroups with and without NMR metabolomics and MRI profiling.
**TABLE S5:** Risk prediction models for liver fat concentrations (PDFF) comparing conventional risk factors and NMR metabolites.
**TABLE S6:** Characterization of the variation in NMR metabolites explained by the first 10 metabolic NMR‐biomarker principal components (PC).
