# Supplementary material for: Metabolic profiling of MRI‐measured liver fat in the UK Biobank
Source: Obesity (Silver Spring). 2023 Mar 5;31(4):1121–32. doi: 10.1002/oby.23687 (PMC11423419; doi:10.1002/oby.23687)
Supplement: Supplementary file 1 — FIGURE S1: Distributions of liver fat and selected NMR metabolites (log‐values). FIGURE S2: Correlation map of direct measures of NMR metabolites. TABLE S1: Sex‐specific levels of NMR metabolites. FIGURE S3: Levels of MRI‐measured proton density liver fat fraction by baseline levels of total NMR‐measured lipids. FIGURE S4: NMR metabolites and liver fat by age, sex, and smoking status. TABLE S2: Associations of log‐levels of NMR metabolites (per 1 SD) with levels of liver fat (per 1 SD), by diabetes. TABLE S3: Absolute differences in the associations of log‐levels of NMR metabolites (per 1 SD) with levels of liver fat (per 1 SD), by different characteristics at baseline. TABLE S4: Baseline characteristics of study population subgroups with and without NMR metabolomics and MRI profiling. TABLE S5: Risk prediction models for liver fat concentrations (PDFF) comparing conventional risk factors and NMR metabolites. TABLE S6: Characterization of the variation in NMR metabolites explained by the first 10 metabolic NMR‐biomarker principal components (PC). [file OBY-31-1121-s001.pdf]

Supplemental Tables and Figures

Figure S1: Distributions of liver fat and selected NMR-metabolites (log-values)

Figure S2: Correlation map of direct measures of NMR-metabolites

Table S1: Sex-specific levels of NMR-metabolites

Figure S3: Levels of MRI-measured proton density liver fat fraction by baseline levels of total NMR-measured lipids

Figure S4: NMR metabolites and liver fat by age, sex and smoking status

Table S2: Associations of log-levels of NMR metabolites (per 1 SD) with levels of liver fat (per 1 SD), by diabetes

Table S3: Absolute differences in the associations of log-levels of NMR metabolites (per 1 SD) with levels of liver fat (per 1 SD), by different characteristics at baseline

Table S4: Baseline characteristics of study population sub-groups with and without NMR-metabolomic and MRI profiling

Table S5: Risk prediction models for liver fat concentrations (PDFF) comparing conventional risk factors and NMR-metabolites

Table S6: Characterization of the variation in NMR-metabolites explained by the first 10 metabolic NMR-biomarker principal components (PC)

Figure S1: Distributions of liver fat MRI-measures and log values of selected NMR metabolites

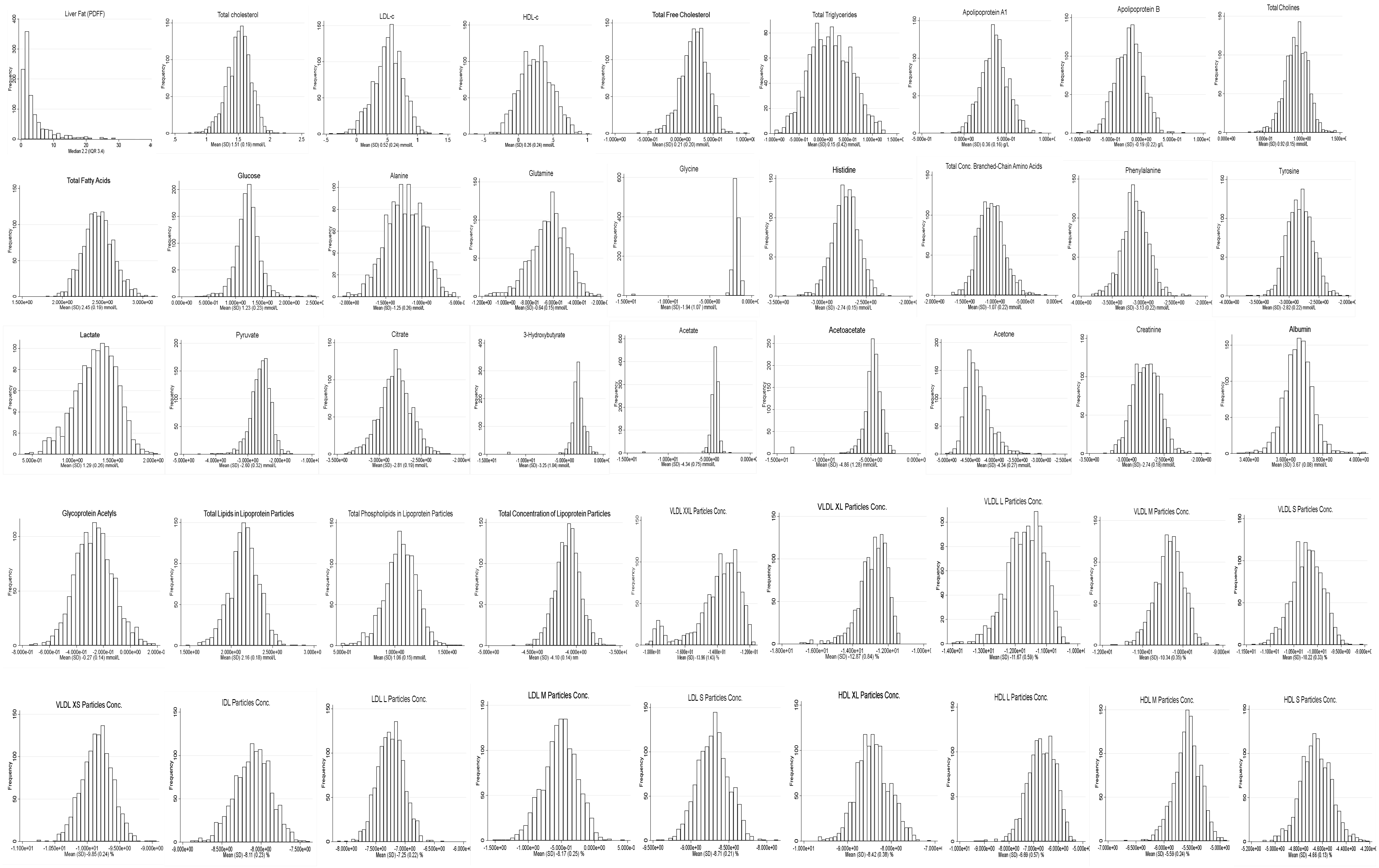

Values corresponding to those included in analyses (see details of footnote of Table 1).

Figure S2: Correlations of the NMR metabolites

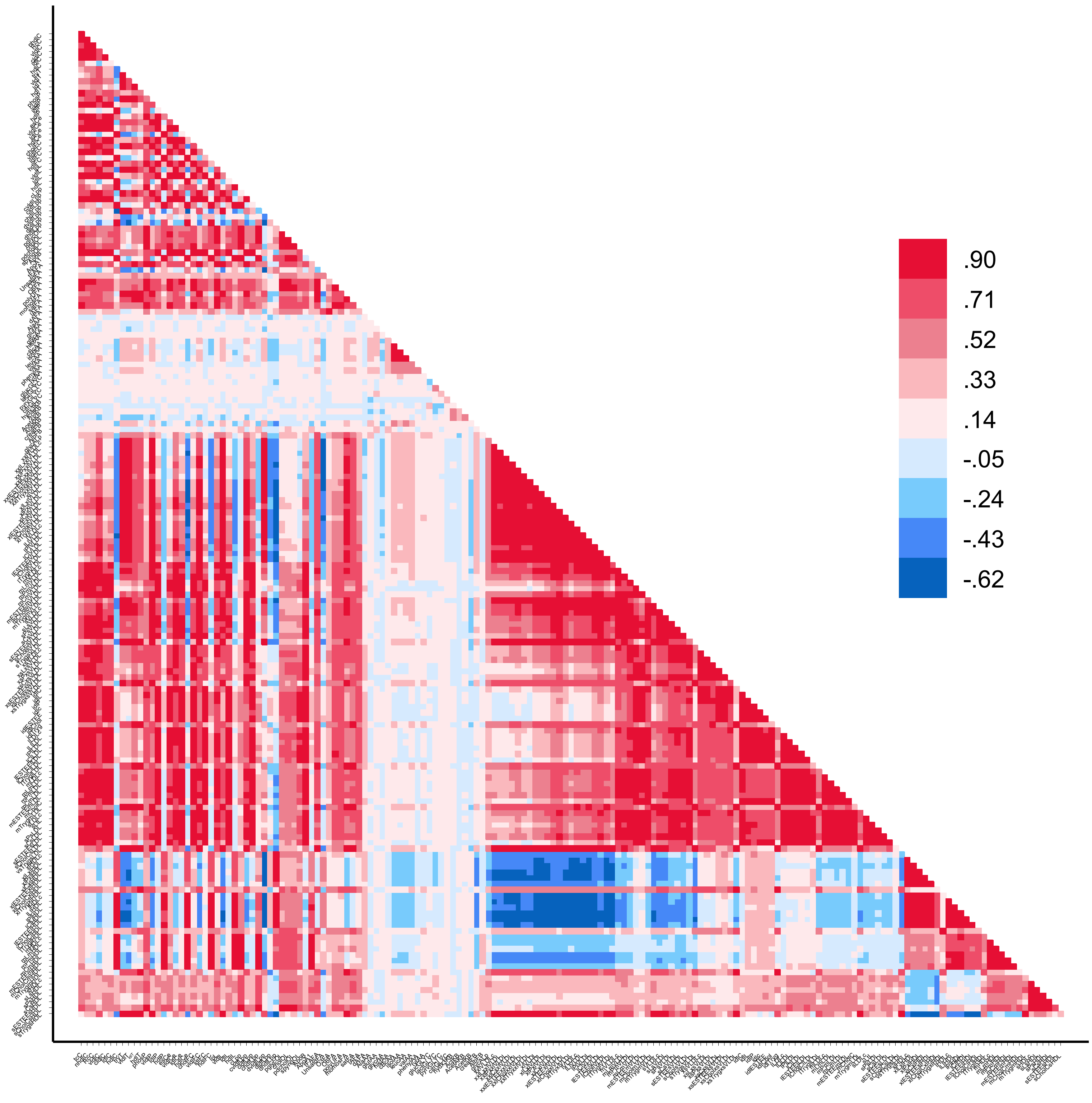

Spearman correlates between absolute measures of the NMR metabolites. The intensity of the colours reflect the strength of the correlation coefficients. Label details as per table S1; they follow the same s equence from right to left for X-axis, and from top to bottom for the Y-axis.

Table S1: Sex-specific values of direct and derived measures of NMR metabolites

Metabolites	Label	Men				Women				All			
		Mean	SD	Median	IQR	Mean	SD	Median	IQR	Mean	SD	Median	IQR
**Cholesterol (mmol/L)**													
Total Cholesterol	TC-C	4.42	0.86	4.40	1.15	4.79	0.82	4.78	1.07	4.62	0.86	4.59	1.09
Non HDL Cholesterol	nHDL-C	3.25	0.79	3.22	1.02	3.37	0.74	3.32	1.02	3.31	0.77	3.26	1.01
Remnant Cholesterol	RC-C	1.53	0.39	1.53	0.50	1.61	0.37	1.59	0.50	1.57	0.38	1.56	0.50
VLDL Cholesterol	VLDL-C	0.73	0.23	0.71	0.32	0.72	0.22	0.70	0.29	0.73	0.22	0.71	0.30
Clinical LDL Cholesterol	cLDL-C	2.51	0.68	2.50	0.87	2.60	0.65	2.55	0.86	2.56	0.66	2.53	0.86
LDL Cholesterol	LDL-C	1.72	0.41	1.70	0.54	1.76	0.39	1.73	0.51	1.74	0.40	1.72	0.53
HDL Cholesterol	HDL-C	1.17	0.26	1.14	0.33	1.43	0.30	1.39	0.39	1.31	0.31	1.27	0.40
**Triglycerides (mmol/L)**													
Total Triglycerides	Try-T	1.37	0.55	1.29	0.72	1.22	0.51	1.09	0.65	1.29	0.53	1.18	0.72
Triglycerides in VLDL	VLDL-T	1.00	0.46	0.93	0.61	0.84	0.43	0.72	0.56	0.92	0.45	0.81	0.61
Triglycerides in LDL	LDL-T	0.14	0.04	0.13	0.05	0.14	0.04	0.13	0.04	0.14	0.04	0.13	0.05
Triglycerides in HDL	HDL-T	0.14	0.04	0.13	0.05	0.14	0.04	0.14	0.06	0.14	0.04	0.14	0.05
**Phospholipids (mmol/L)**													
Total Phospholipids in Lipoprotein Particles	Phos-P	2.77	0.43	2.76	0.56	3.03	0.40	3.02	0.53	2.90	0.44	2.89	0.60
Phospholipids in VLDL	VLDL-P	0.49	0.17	0.47	0.24	0.46	0.16	0.43	0.22	0.47	0.17	0.44	0.23
Phospholipids in LDL	LDL-P	0.60	0.13	0.60	0.17	0.61	0.13	0.60	0.17	0.61	0.13	0.60	0.17
Phospholipids in HDL	HDL-P	1.40	0.26	1.37	0.34	1.65	0.28	1.60	0.35	1.53	0.30	1.51	0.38
**Cholesteryl esters (mmol/L)**													
Total Esterified Cholesterol	Es-CE	3.20	0.62	3.19	0.82	3.48	0.59	3.46	0.75	3.35	0.62	3.34	0.80
Cholesteryl Esters in VLDL	VLDL-CE	0.43	0.13	0.42	0.18	0.43	0.13	0.42	0.17	0.43	0.13	0.42	0.17
Cholesteryl Esters in LDL	LDL-CE	1.26	0.30	1.25	0.40	1.28	0.29	1.26	0.39	1.27	0.29	1.26	0.39
Cholesteryl Esters in HDL	HDL-CE	0.91	0.20	0.88	0.26	1.11	0.23	1.08	0.30	1.02	0.24	0.99	0.32
**Free cholesterol (mmol/L)**													
Total Free Cholesterol	Chol-FC	1.22	0.25	1.21	0.32	1.31	0.23	1.30	0.32	1.26	0.25	1.26	0.32
Free Cholesterol in VLDL	VLDL-FC	0.30	0.10	0.29	0.14	0.29	0.10	0.27	0.13	0.29	0.10	0.28	0.13
Free Cholesterol in LDL	LDL-FC	0.46	0.11	0.45	0.14	0.48	0.11	0.47	0.14	0.47	0.11	0.46	0.14
Free Cholesterol in HDL	HDL-FC	0.26	0.06	0.25	0.07	0.32	0.07	0.31	0.09	0.29	0.07	0.28	0.09
**Total lipids (mmol/L)**													
Total Lipids in Lipoprotein Particles	Lp-L	8.56	1.58	8.53	1.96	9.04	1.43	9.00	1.90	8.81	1.52	8.75	1.87
Total Lipids in VLDL	VLDL-L	2.22	0.81	2.13	1.06	2.02	0.77	1.85	1.02	2.12	0.79	1.97	1.08
Total Lipids in LDL	LDL-L	2.46	0.56	2.43	0.72	2.51	0.53	2.46	0.72	2.49	0.55	2.45	0.72
Total Lipids in HDL	HDL-L	2.71	0.51	2.65	0.66	3.22	0.57	3.13	0.74	2.98	0.60	2.92	0.77

Table S1 continued		Mean	SD	Median	IQR	Mean	SD	Median	IQR	Mean	SD	Median	IQR
**Lipoprotein concentrations (nm)**													
Total Concentration of Lipoprotein Particles	c-LPp	0.016	0.002	0.016	0.003	0.017	0.002	0.017	0.003	0.017	0.002	0.017	0.003
Concentration of VLDL Particles	cVLDL-LPp	0.00015	0.00004	0.00014	0.00006	0.00014	0.00004	0.00014	0.00005	0.00014	0.00004	0.00014	0.00005
Concentration of LDL Particles	cLDL-LPp	0.0012	0.0003	0.0012	0.0003	0.0012	0.0002	0.0012	0.0003	0.0012	0.0003	0.0012	0.0003
Concentration of HDL Particles	cHDL-LPp	0.014	0.002	0.014	0.003	0.016	0.002	0.015	0.003	0.015	0.002	0.015	0.003
Average Diameter for VLDL Particles	dVLDL-LPs	39.06	1.08	39.08	1.50	38.35	1.10	38.19	1.66	38.69	1.15	38.66	1.76
Average Diameter for LDL Particles	dLDL-LPs	23.90	0.09	23.91	0.13	23.94	0.08	23.94	0.11	23.92	0.08	23.93	0.12
Average Diameter for HDL Particles	dHDL-LPs	9.55	0.16	9.52	0.19	9.73	0.19	9.71	0.30	9.64	0.20	9.61	0.28
**Other lipids (mmol/L)**													
Phosphoglycerides	Pglyc-OL	2.13	0.36	2.10	0.46	2.37	0.34	2.35	0.46	2.25	0.37	2.25	0.51
Phosphoglycerides:Tryglicerides Ratio	Pglyc:Tryg-OL	0.64	0.21	0.63	0.30	0.51	0.19	0.46	0.26	0.57	0.21	0.54	0.31
Total Cholines	Chol-OL	2.40	0.37	2.39	0.48	2.66	0.35	2.64	0.48	2.54	0.38	2.53	0.52
Phosphatidylcholines	Pdchol-OL	1.96	0.33	1.93	0.44	2.20	0.32	2.19	0.43	2.08	0.35	2.09	0.49
Sphingomyelins	Spyng-OL	0.42	0.06	0.42	0.08	0.46	0.06	0.46	0.08	0.44	0.07	0.44	0.09
Apolipoproteins (g/L)													
Apolipoprotein B	Apo-B	0.85	0.19	0.83	0.24	0.86	0.18	0.84	0.24	0.85	0.18	0.84	0.24
Apolipoprotein A1	Apo-A1	1.34	0.20	1.32	0.27	1.52	0.22	1.49	0.27	1.44	0.23	1.42	0.29
ApoB:ApoA Ratio	ApoB:ApoA	0.64	0.16	0.63	0.23	0.57	0.14	0.56	0.20	0.61	0.16	0.59	0.22
**Fatty acids (mmol/L)**													
Total Fatty Acids	TFA-FA	11.62	2.36	11.37	2.90	12.02	2.10	11.85	2.73	11.83	2.23	11.60	2.86
Degree of Unsaturation	UnsatD-FA	1.35	0.08	1.35	0.09	1.38	0.07	1.38	0.09	1.37	0.08	1.37	0.10
Omega-3 Fatty Acids	O3-FA	0.50	0.21	0.47	0.26	0.55	0.20	0.52	0.26	0.53	0.21	0.50	0.27
Omega-6 Fatty Acids	O6-FA	4.36	0.65	4.32	0.87	4.61	0.62	4.57	0.81	4.49	0.65	4.45	0.82
Polyunsaturated Fatty Acids	PolyU-FA	4.87	0.77	4.80	1.01	5.17	0.73	5.14	0.98	5.02	0.76	4.99	1.02
Monounsaturated Fatty Acids	MomoS-FA	2.80	0.79	2.68	1.00	2.77	0.71	2.63	0.91	2.79	0.75	2.65	0.95
Saturated Fatty Acids	Sat-FA	3.95	0.95	3.84	1.15	4.08	0.81	3.97	1.03	4.02	0.88	3.91	1.08
Linoleic Acid	LA-FA	3.35	0.66	3.30	0.86	3.58	0.63	3.52	0.82	3.47	0.66	3.44	0.82
Docosahexaenoic Acid	DA-FA	0.22	0.08	0.21	0.09	0.25	0.08	0.24	0.10	0.24	0.08	0.23	0.09
Omega-3: Total Fatty Acids %	O3:Tot-FA%	4.32	1.48	4.11	1.86	4.57	1.47	4.40	1.86	4.45	1.48	4.27	1.87
Omega-6: Total Fatty Acids %	O6:Tot-FA%	38.04	3.82	38.32	4.88	38.76	3.19	39.16	4.02	38.42	3.52	38.88	4.65
Polyunsaturated:Total Fatty Acids %	PolyU:Tot-FA%	42.38	3.85	42.76	5.11	43.34	3.29	43.83	3.95	42.88	3.60	43.44	4.43
Monounsaturated: Total Fatty Acids %	MomoS:Tot-FA	23.82	2.56	23.49	3.54	22.84	2.32	22.58	2.99	23.31	2.49	23.01	3.36
Saturated Fatty: Total Acids %	Sat: Tot-FA%	33.77	2.02	33.69	2.68	33.82	1.75	33.73	2.30	33.80	1.88	33.71	2.47
Linoleic: Total F Acid%	LA: Tot-FA%	29.10	3.62	29.28	4.91	29.95	3.15	30.04	4.35	29.54	3.41	29.70	4.65
Docosahexaenoic Acid: Total Acids%	DA: Tot-FA%	1.94	0.64	1.84	0.83	2.11	0.64	2.06	0.79	2.03	0.65	1.95	0.81
Polyunsaturated: Monounsaturated Acids %	PolyU: MomoS-FA	1.81	0.34	1.82	0.48	1.93	0.31	1.95	0.42	1.87	0.33	1.90	0.45
Omega-6:Omega-3 Fatty Acids %	O6: O3-FA	9.71	3.70	8.98	4.48	9.39	3.39	8.72	4.06	9.54	3.55	8.80	4.28

Table S1 continued		Mean	SD	Median	IQR	Mean	SD	Median	IQR	Mean	SD	Median	IQR
**Amino acids (mmol/L)**													
Alanine	Ala-AA	0.30	0.07	0.30	0.11	0.29	0.08	0.28	0.10	0.30	0.08	0.29	0.11
Glutamine	Glut-AA	0.54	0.08	0.54	0.11	0.52	0.08	0.52	0.10	0.53	0.08	0.53	0.11
Glycine	Glyc-AA	0.14	0.04	0.14	0.05	0.18	0.06	0.17	0.08	0.16	0.06	0.15	0.07
Histidine	Hist-AA	0.07	0.01	0.07	0.01	0.06	0.01	0.06	0.01	0.07	0.01	0.07	0.01
Tot. Conc. Branched-Chain Amino Acids	C-BC-AA	0.37	0.07	0.36	0.09	0.33	0.07	0.32	0.10	0.35	0.08	0.34	0.10
Isoleucine	Isol-AA	0.05	0.02	0.05	0.02	0.05	0.01	0.04	0.02	0.05	0.02	0.05	0.02
Leucine	Leuc-AA	0.11	0.02	0.10	0.03	0.09	0.02	0.09	0.03	0.10	0.02	0.10	0.03
Valine	Val-AA	0.21	0.04	0.21	0.05	0.19	0.04	0.19	0.05	0.20	0.04	0.20	0.05
Phenylalanine	Phenyl-AA	0.05	0.01	0.04	0.01	0.04	0.01	0.04	0.01	0.04	0.01	0.04	0.01
Tyrosine	Tyr-AA	0.06	0.01	0.06	0.02	0.06	0.01	0.06	0.02	0.06	0.01	0.06	0.02
**Glycolysis (mmol/L)**													
Glucose	Gluc-Glyc	3.45	0.72	3.41	0.80	3.50	0.72	3.43	0.83	3.47	0.72	3.42	0.82
Lactate	Lact-Glyc	3.79	0.96	3.79	1.32	3.70	0.91	3.66	1.27	3.74	0.94	3.72	1.31
Pyruvate	Pyrv-Glyc	0.07	0.02	0.07	0.03	0.08	0.02	0.08	0.03	0.08	0.02	0.08	0.03
Citrate	Citr-Glyc	0.06	0.01	0.06	0.01	0.06	0.01	0.06	0.01	0.06	0.01	0.06	0.01
**Ketone bodies (mmol/L)**													
3-Hydroxybutyrate	3Hydx-KB	0.05	0.04	0.04	0.03	0.05	0.04	0.04	0.03	0.05	0.04	0.04	0.03
Acetate	Act-KB	0.01	0.01	0.01	0.01	0.01	0.01	0.01	0.01	0.01	0.01	0.01	0.01
Acetoacetate	AcetA-KB	0.01	0.01	0.01	0.01	0.01	0.01	0.01	0.01	0.01	0.01	0.01	0.01
Acetone	Acet-KB	0.013	0.004	0.013	0.004	0.013	0.004	0.012	0.004	0.013	0.004	0.012	0.004
**Fluid balance (mmol/L)**													
Creatinine	Creat-FB	0.07	0.01	0.07	0.01	0.06	0.01	0.06	0.01	0.07	0.01	0.07	0.02
Albumin	Alb-FB	39.31	3.02	39.36	3.70	39.07	3.01	39.00	3.55	39.18	3.02	39.22	3.67
**Inflammation (mmol/L)**													
Glycoprotein Acetyls	GlycA-LP	0.77	0.11	0.76	0.14	0.78	0.11	0.77	0.15	0.78	0.11	0.77	0.14
**Lipoproteins concentrations**													
**VLDL particles - Extremely Large**													
Concentration of VLDL Particles	XXL-VLDL	0.0000	0.0000	0.0000	0.0000	0.0000	0.0000	0.0000	0.0000	0.0000	0.0000	0.0000	0.0000
Total Lipids in VLDL	XXL-L	0.26	0.19	0.23	0.26	0.20	0.18	0.13	0.22	0.23	0.19	0.17	0.25
Phospholipids in VLDL	XXL-PL	0.04	0.03	0.04	0.04	0.03	0.03	0.02	0.03	0.04	0.03	0.03	0.04
Cholesterol in VLDL	XXL-C	0.06	0.04	0.06	0.05	0.05	0.04	0.04	0.05	0.06	0.04	0.05	0.05
Cholesteryl Esters in VLDL	XXL-Este-E	0.04	0.02	0.03	0.03	0.03	0.02	0.02	0.03	0.03	0.02	0.03	0.03
Free Cholesterol in VLDL	XXL-Chol-C	0.03	0.02	0.03	0.02	0.02	0.02	0.02	0.02	0.03	0.02	0.02	0.02
Triglycerides in VLDL	XXL-Try	0.16	0.13	0.14	0.17	0.12	0.12	0.08	0.14	0.14	0.12	0.10	0.16

Table S1 continued		Mean	SD	Median	IQR	Mean	SD	Median	IQR	Mean	SD	Median	IQR
**VLDL particles -Very Large**													
Concentration of VLDL Particles	XL-VLDL	0.00000	0.00000	0.00000	0.00000	0.00000	0.00000	0.00000	0.00000	0.00000	0.00000	0.00000	0.00000
Total Lipids in VLDL	XL-L	0.23	0.13	0.21	0.17	0.18	0.12	0.15	0.16	0.21	0.13	0.18	0.17
Phospholipids in VLDL	XL-Phos	0.04	0.02	0.04	0.03	0.03	0.02	0.03	0.03	0.04	0.02	0.03	0.03
Cholesterol in VLDL	XL-C	0.06	0.03	0.06	0.04	0.05	0.03	0.04	0.04	0.05	0.03	0.05	0.04
Cholesteryl Esters in VLDL	XL-Este-E	0.03	0.01	0.03	0.02	0.03	0.01	0.03	0.02	0.03	0.01	0.03	0.02
Free Cholesterol in VLDL	XL-Chol-C	0.03	0.01	0.03	0.02	0.02	0.01	0.02	0.02	0.02	0.01	0.02	0.02
Triglycerides in VLDL	XL-Try	0.13	0.08	0.11	0.10	0.10	0.07	0.07	0.09	0.11	0.08	0.09	0.10
**VLDL particles - Large**													
Concentration of VLDL Particles	L-VLDL	0.00001	0.00001	0.00001	0.00001	0.00001	0.00000	0.00001	0.00001	0.00001	0.00001	0.00001	0.00001
Total Lipids in VLDL	L-L	0.37	0.16	0.35	0.22	0.30	0.16	0.27	0.21	0.33	0.16	0.30	0.23
Phospholipids in VLDL	L-P	0.07	0.04	0.07	0.05	0.06	0.04	0.05	0.05	0.07	0.04	0.06	0.05
Cholesterol in VLDL	L-C	0.11	0.04	0.10	0.06	0.09	0.04	0.08	0.06	0.10	0.04	0.09	0.06
Cholesteryl Esters in VLDL	L-Este-E	0.05	0.02	0.05	0.03	0.05	0.02	0.05	0.03	0.05	0.02	0.05	0.03
Free Cholesterol in VLDL	L-Chol-C	0.05	0.02	0.05	0.03	0.04	0.02	0.04	0.03	0.05	0.02	0.04	0.03
Triglycerides in VLDL	L-Tryg	0.19	0.09	0.17	0.12	0.15	0.08	0.13	0.11	0.17	0.09	0.15	0.12
**VLDL particles - Medium**													
Concentration of VLDL Particles	M-VLDL	0.00004	0.00001	0.00003	0.00001	0.00003	0.00001	0.00003	0.00001	0.00004	0.00001	0.00003	0.00001
Total Lipids in VLDL	M-L	0.60	0.19	0.58	0.26	0.56	0.18	0.54	0.24	0.58	0.19	0.55	0.25
Phospholipids in VLDL	M-P	0.13	0.04	0.13	0.06	0.13	0.04	0.12	0.06	0.13	0.04	0.12	0.06
Cholesterol in VLDL	M-C	0.17	0.06	0.17	0.08	0.18	0.06	0.17	0.08	0.17	0.06	0.17	0.08
Cholesteryl Esters in VLDL	M-Este-E	0.09	0.04	0.09	0.05	0.10	0.03	0.10	0.05	0.09	0.03	0.09	0.05
Free Cholesterol in VLDL	M-Chol-C	0.08	0.03	0.08	0.04	0.08	0.03	0.08	0.03	0.08	0.03	0.08	0.04
Triglycerides in VLDL	M-Tryg	0.29	0.11	0.28	0.15	0.26	0.10	0.23	0.14	0.28	0.11	0.26	0.15
**VLDL particles - Small**													
Concentration of VLDL Particles	S-VLDL	0.00004	0.00001	0.00004	0.00002	0.00004	0.00001	0.00004	0.00001	0.00004	0.00001	0.00004	0.00002
Total Lipids in VLDL	S-L	0.42	0.12	0.41	0.16	0.40	0.12	0.38	0.15	0.41	0.12	0.39	0.16
Phospholipids in VLDL	S-P	0.10	0.03	0.09	0.04	0.10	0.03	0.09	0.03	0.10	0.03	0.09	0.04
Cholesterol in VLDL	S-C	0.16	0.05	0.15	0.06	0.16	0.05	0.15	0.06	0.16	0.05	0.15	0.06
Cholesteryl Esters in VLDL	S-Este-E	0.10	0.03	0.10	0.04	0.10	0.03	0.09	0.04	0.10	0.03	0.09	0.04
Free Cholesterol in VLDL	S-Chol-C	0.06	0.02	0.06	0.02	0.06	0.02	0.06	0.02	0.06	0.02	0.06	0.02
Triglycerides in VLDL	S-Tryg	0.16	0.06	0.16	0.07	0.15	0.05	0.14	0.07	0.16	0.06	0.15	0.07
**VLDL particles - Very Small**													
Concentration of VLDL Particles	XS-VLDL	0.00005	0.00001	0.00005	0.00002	0.00006	0.00001	0.00006	0.00002	0.00005	0.00001	0.00005	0.00002
Total Lipids in VLDL	XS-L	0.34	0.08	0.33	0.11	0.37	0.08	0.37	0.10	0.36	0.08	0.35	0.10
Phospholipids in VLDL	XS-P	0.10	0.02	0.10	0.03	0.11	0.02	0.11	0.03	0.10	0.02	0.10	0.03
Cholesterol in VLDL	XS-C	0.18	0.04	0.17	0.06	0.20	0.04	0.20	0.06	0.19	0.04	0.18	0.06
Cholesteryl Esters in VLDL	XS-Este-E	0.12	0.03	0.12	0.04	0.14	0.03	0.14	0.04	0.13	0.03	0.13	0.04

Table S1 continued		Mean	SD	Median	IQR	Mean	SD	Median	IQR	Mean	SD	Median	IQR
Free Cholesterol in VLDL	XS-Chol-C	0.06	0.01	0.05	0.02	0.06	0.01	0.06	0.02	0.06	0.01	0.06	0.02
Triglycerides in VLDL	XS-Tryg	0.07	0.02	0.06	0.02	0.07	0.02	0.07	0.02	0.07	0.02	0.06	0.02
**IDL Particles**													
Concentration of IDL Particles	IDL-C	0.0003	0.0001	0.0003	0.0001	0.0003	0.0001	0.0003	0.0001	0.0003	0.0001	0.0003	0.0001
Total Lipids in IDL	IDL-L	1.17	0.26	1.17	0.34	1.29	0.25	1.29	0.34	1.23	0.26	1.22	0.34
Phospholipids in IDL	IDL-P	0.28	0.06	0.28	0.08	0.31	0.06	0.31	0.08	0.29	0.06	0.29	0.08
Cholesterol in IDL	IDL-C	0.80	0.19	0.80	0.26	0.89	0.19	0.88	0.24	0.84	0.20	0.84	0.26
Cholesteryl Esters in IDL	IDL-Este-E	0.59	0.14	0.59	0.19	0.65	0.14	0.65	0.18	0.62	0.15	0.62	0.19
Free Cholesterol in IDL	IDL-Chol-C	0.21	0.05	0.21	0.06	0.23	0.05	0.23	0.07	0.22	0.05	0.22	0.07
Triglycerides in IDL	IDL-Tryg	0.10	0.02	0.09	0.03	0.10	0.02	0.10	0.03	0.10	0.02	0.09	0.03
**LDL Particles - Large**													
Concentration of LDL Particles	L-LDL	0.0007	0.0002	0.0007	0.0002	0.0007	0.0002	0.0007	0.0002	0.0007	0.0002	0.0007	0.0002
Total Lipids in LDL	L-L	1.55	0.35	1.53	0.45	1.61	0.33	1.59	0.44	1.58	0.34	1.57	0.44
Phospholipids in LDL	L-P	0.35	0.08	0.35	0.10	0.36	0.07	0.36	0.10	0.36	0.08	0.35	0.10
Cholesterol in LDL	L-C	1.10	0.26	1.09	0.33	1.16	0.25	1.14	0.34	1.13	0.26	1.12	0.33
Cholesteryl Esters in LDL	L-Este-E	0.82	0.19	0.80	0.25	0.85	0.18	0.84	0.25	0.83	0.19	0.82	0.25
Free Cholesterol in LDL	L-Chol-C	0.29	0.07	0.28	0.09	0.31	0.07	0.30	0.09	0.30	0.07	0.29	0.09
Triglycerides in LDL	L-Tryg	0.09	0.02	0.09	0.03	0.09	0.02	0.09	0.03	0.09	0.02	0.09	0.03
**LDL Particles - Medium**													
Concentration of LDL Particles	M-LDL	0.00030	0.00007	0.00030	0.00009	0.00029	0.00007	0.00029	0.00009	0.00030	0.00007	0.00029	0.00009
Total Lipids in LDL	M-L	0.63	0.16	0.61	0.20	0.61	0.15	0.60	0.19	0.62	0.15	0.61	0.20
Phospholipids in LDL	M-P	0.16	0.04	0.16	0.05	0.16	0.04	0.15	0.05	0.16	0.04	0.16	0.05
Cholesterol in LDL	M-C	0.43	0.11	0.42	0.14	0.42	0.10	0.41	0.14	0.43	0.11	0.42	0.14
Cholesteryl Esters in LDL	M-Este-E	0.31	0.08	0.31	0.10	0.30	0.08	0.29	0.10	0.31	0.08	0.30	0.10
Free Cholesterol in LDL	M-Chol-C	0.12	0.03	0.12	0.04	0.12	0.03	0.12	0.04	0.12	0.03	0.12	0.04
Triglycerides in LDL	M-Tryg	0.03	0.01	0.03	0.01	0.03	0.01	0.03	0.01	0.03	0.01	0.03	0.01
**LDL Particles - Small**													
Concentration of LDL Particles	S-LDL	0.00017	0.00003	0.00017	0.00005	0.00017	0.00003	0.00017	0.00004	0.00017	0.00003	0.00017	0.00005
Total Lipids in LDL	S-L	0.29	0.06	0.28	0.08	0.28	0.06	0.28	0.08	0.28	0.06	0.28	0.08
Phospholipids in LDL	S-P	0.09	0.02	0.09	0.02	0.09	0.02	0.09	0.02	0.09	0.02	0.09	0.02
Cholesterol in LDL	S-C	0.18	0.04	0.18	0.05	0.18	0.04	0.18	0.05	0.18	0.04	0.18	0.05
Cholesteryl Esters in LDL	S-Este-E	0.13	0.03	0.13	0.04	0.13	0.03	0.13	0.04	0.13	0.03	0.13	0.04
Free Cholesterol in LDL	S-Chol-C	0.05	0.01	0.05	0.01	0.05	0.01	0.05	0.01	0.05	0.01	0.05	0.01
Triglycerides in LDL	S-Tryg	0.02	0.01	0.01	0.01	0.01	0.00	0.01	0.01	0.02	0.01	0.01	0.01

Table S1 continued		Mean	SD	Median	IQR	Mean	SD	Median	IQR	Mean	SD	Median	IQR
**HDL Particles - Very Large**													
Concentration of HDL Particles	XL-HDL	0.0002	0.0001	0.0002	0.0001	0.0003	0.0001	0.0002	0.0001	0.0002	0.0001	0.0002	0.0001
Total Lipids in HDL	XL-L	0.14	0.06	0.13	0.06	0.20	0.08	0.18	0.11	0.17	0.08	0.15	0.09
Phospholipids in HDL	XL-P	0.06	0.03	0.06	0.03	0.10	0.04	0.09	0.06	0.08	0.04	0.07	0.05
Cholesterol in HDL	XL-C	0.07	0.02	0.07	0.03	0.09	0.03	0.09	0.05	0.08	0.03	0.08	0.04
Cholesteryl Esters in HDL	XL-Este-E	0.05	0.02	0.04	0.02	0.07	0.03	0.06	0.04	0.06	0.03	0.05	0.03
Free Cholesterol in HDL	XL-Chol-C	0.02	0.01	0.02	0.01	0.03	0.01	0.02	0.01	0.02	0.01	0.02	0.01
Triglycerides in HDL	XL-Tryg	0.007	0.002	0.006	0.003	0.007	0.002	0.007	0.003	0.007	0.002	0.007	0.003
**HDL Particles - Large**													
Concentration of HDL Particles	L-HDL	0.0011	0.0006	0.0009	0.0006	0.0017	0.0007	0.0016	0.0010	0.0014	0.0007	0.0012	0.0010
Total Lipids in HDL	L-L	0.50	0.24	0.45	0.26	0.78	0.31	0.75	0.43	0.65	0.31	0.57	0.42
Phospholipids in HDL	L-P	0.25	0.11	0.23	0.12	0.38	0.14	0.36	0.20	0.32	0.14	0.29	0.20
Cholesterol in HDL	L-C	0.22	0.12	0.19	0.13	0.36	0.16	0.34	0.22	0.30	0.16	0.26	0.22
Cholesteryl Esters in HDL	L-Este-E	0.17	0.10	0.15	0.10	0.28	0.12	0.26	0.17	0.23	0.12	0.20	0.17
Free Cholesterol in HDL	L-Chol-C	0.05	0.03	0.04	0.03	0.08	0.04	0.08	0.05	0.07	0.04	0.06	0.05
Triglycerides in HDL	L-Tryg	0.03	0.01	0.03	0.01	0.03	0.01	0.03	0.01	0.03	0.01	0.03	0.02
**HDL Particles - Medium**													
Concentration of HDL Particles	M-HDL	0.0034	0.0008	0.0034	0.0010	0.0041	0.0008	0.0040	0.0011	0.0038	0.0009	0.0037	0.0011
Total Lipids in HDL	M-L	0.94	0.19	0.92	0.25	1.09	0.20	1.06	0.25	1.02	0.21	1.01	0.26
Phospholipids in HDL	M-P	0.44	0.09	0.44	0.11	0.51	0.09	0.50	0.11	0.48	0.09	0.47	0.12
Cholesterol in HDL	M-C	0.44	0.10	0.43	0.13	0.53	0.11	0.51	0.14	0.49	0.11	0.48	0.15
Cholesteryl Esters in HDL	M-Este-E	0.37	0.08	0.36	0.11	0.43	0.09	0.42	0.12	0.40	0.09	0.39	0.12
Free Cholesterol in HDL	M-Chol-C	0.08	0.02	0.07	0.03	0.09	0.02	0.09	0.03	0.09	0.02	0.08	0.03
Triglycerides in HDL	M-Tryg	0.05	0.02	0.05	0.02	0.05	0.02	0.05	0.02	0.05	0.02	0.05	0.02
**HDL Particles - Small**													
Concentration of HDL Particles	S-HDL	0.010	0.001	0.009	0.002	0.010	0.001	0.010	0.002	0.010	0.001	0.009	0.002
Total Lipids in HDL	S-L	1.13	0.15	1.12	0.18	1.16	0.15	1.14	0.20	1.14	0.15	1.13	0.20
Phospholipids in HDL	S-P	0.64	0.09	0.63	0.11	0.66	0.09	0.66	0.11	0.65	0.09	0.65	0.11
Cholesterol in HDL	S-C	0.44	0.06	0.43	0.07	0.44	0.06	0.44	0.08	0.44	0.06	0.44	0.08
Cholesteryl Esters in HDL	S-Este-E	0.33	0.04	0.32	0.06	0.33	0.05	0.32	0.06	0.33	0.04	0.32	0.06
Free Cholesterol in HDL	S-Chol-C	0.11	0.02	0.11	0.02	0.12	0.01	0.11	0.02	0.11	0.02	0.11	0.02
Triglycerides in HDL	S-Tryg	0.05	0.02	0.05	0.02	0.05	0.02	0.05	0.02	0.05	0.02	0.05	0.02
**VLDL % - Extremely Large**													
Phospholipids:Tot. Lipids	P:L-XXL VLDL	15.63	3.08	15.63	1.95	14.82	4.27	15.51	2.21	15.20	3.77	15.58	2.03
Cholesterol:Tot. Lipids	C:L-XXL VLDL	27.34	8.59	25.05	6.87	29.31	10.40	26.18	8.48	28.37	9.63	25.65	7.91
Cholesteryl Esters:Tot. Lipids	CE:L-XXL VLDL	15.19	5.68	13.82	4.66	15.89	6.63	14.16	5.79	15.56	6.21	14.04	5.19
Free Cholesterol:Tot. Lipids	CholC:L-XXL VLDL	11.96	3.11	11.13	2.39	13.08	3.76	12.09	3.40	12.54	3.51	11.62	3.03
Triglicerydes:Tot. Lipids	Tryg:L-XXL VLDL	57.25	9.62	58.89	8.19	56.42	10.80	58.56	9.79	56.81	10.26	58.74	8.94

Table S1 continued		Mean	SD	Median	IQR	Mean	SD	Median	IQR	Mean	SD	Median	IQR
**VLDL % - Very Large**													
Phospholipids:Tot. Lipids	P:L-XL VLDL	18.84	1.52	19.04	1.44	18.68	1.93	18.95	1.83	18.76	1.74	18.98	1.63
Cholesterol:Tot. Lipids	C:L-XL VLDL	27.49	5.38	26.94	7.14	30.32	6.68	29.53	8.44	28.97	6.26	28.07	8.50
Cholesteryl Esters:Tot. Lipids	CE:L-XL VLDL	15.67	4.37	15.10	5.65	17.85	5.36	17.20	6.73	16.81	5.03	16.04	6.67
Free Cholesterol:Tot. Lipids	CholC:L-XL VLDL	11.82	1.17	11.78	1.45	12.47	1.53	12.23	1.92	12.16	1.40	11.94	1.71
Triglicerydes:Tot. Lipids	Tryg:L-XL VLDL	53.67	5.88	54.03	7.75	50.94	7.05	51.65	9.19	52.24	6.66	52.97	8.80
**VLDL % - Large**													
Phospholipids:Tot. Lipids	P:L-L VLDL	19.69	2.20	20.14	1.78	18.76	2.74	19.49	2.73	19.21	2.54	19.81	2.25
Cholesterol:Tot. Lipids	C:L-L VLDL	29.65	3.50	29.48	4.69	31.00	3.85	30.68	5.59	30.36	3.75	30.18	5.08
Cholesteryl Esters:Tot. Lipids	CE:L-L VLDL	15.57	2.83	15.51	3.93	17.16	3.15	17.10	4.34	16.41	3.10	16.21	4.42
Free Cholesterol:Tot. Lipids	CholC:L-L VLDL	14.08	1.00	14.10	1.07	13.85	1.14	13.86	1.38	13.95	1.08	13.97	1.21
Triglicerydes:Tot. Lipids in Large VLDL %	Tryg:L-L VLDL	50.66	4.38	50.69	4.92	50.23	4.86	50.19	6.15	50.43	4.64	50.51	5.49
**VLDL % - Medium**													
Phospholipids:Tot. Lipids	P:L-M VLDL	22.03	1.54	22.01	2.13	22.77	1.50	22.91	2.02	22.42	1.56	22.47	2.10
Cholesterol:Tot. Lipids	C:L-M VLDL	28.84	5.88	28.99	8.08	31.98	5.89	32.73	7.95	30.49	6.09	30.80	8.71
Cholesteryl Esters:Tot. Lipids	CE:L-M VLDL	15.37	4.41	15.38	6.06	17.74	4.45	18.24	6.20	16.61	4.59	16.82	6.59
Free Cholesterol:Tot. Lipids	CholC:L-M VLDL	13.47	1.51	13.50	2.04	14.24	1.49	14.39	2.05	13.87	1.55	14.01	2.16
Triglicerydes:Tot. Lipids in Medium VLDL %	Tryg:L-M VLDL	49.14	7.36	48.93	10.30	45.25	7.29	44.27	10.09	47.10	7.57	46.66	10.61
**VLDL % - Small**													
Phospholipids:Tot. Lipids	P:L-S VLDL	23.46	1.86	23.42	2.41	24.18	1.85	24.21	2.41	23.84	1.89	23.81	2.54
Cholesterol:Tot. Lipids	C:L-S VLDL	37.83	4.30	37.96	5.84	38.87	4.23	39.15	5.55	38.38	4.30	38.59	5.84
Cholesteryl Esters:Tot. Lipids	CE:L-S VLDL	23.73	2.56	23.85	3.45	24.02	2.53	24.12	3.16	23.88	2.54	23.98	3.41
Free Cholesterol:Tot. Lipids	CholC:L-S VLDL	14.10	1.96	14.11	2.58	14.88	1.97	14.87	2.51	14.51	2.00	14.53	2.63
Triglicerydes:Tot. Lipids in Small VLDL %	Tryg:L-S VLDL	38.71	6.07	38.79	8.35	36.94	5.96	36.80	8.00	37.78	6.07	37.49	8.02
**VLDL % - Very Small**													
Phospholipids:Tot. Lipids	P:L-XS VLDL	29.27	0.80	29.29	1.08	29.02	0.72	28.98	0.93	29.14	0.77	29.10	1.00
Cholesterol:Tot. Lipids	C:L-XS VLDL	51.05	4.25	51.54	5.59	52.88	3.67	53.35	4.37	52.01	4.06	52.67	5.07
Cholesteryl Esters:Tot. Lipids	CE:L-XS VLDL	34.88	3.85	35.32	5.23	36.71	3.33	37.17	3.90	35.84	3.70	36.41	4.76
Free Cholesterol:Tot. Lipids	CholC:L-XS VLDL	16.14	0.57	16.28	0.66	16.17	0.46	16.21	0.60	16.16	0.51	16.24	0.63
Triglicerydes:Tot. Lipids in VSmall VLDL %	Tryg:L-XS VLDL	19.69	3.82	19.36	5.05	18.11	3.27	17.74	3.80	18.86	3.63	18.29	4.54
**IDL %**													
Phospholipids:Tot. Lipids	P:L-IDL	23.84	0.86	23.81	1.05	23.81	0.88	23.87	1.29	23.82	0.87	23.84	1.17
Cholesterol:Tot. Lipids	C:L-IDL	67.84	2.63	68.34	3.52	68.46	2.38	68.84	2.89	68.17	2.52	68.59	3.15
Cholesteryl Esters:Tot. Lipids	CE:L-IDL	49.88	2.25	50.06	2.88	50.52	2.08	50.71	2.51	50.22	2.19	50.49	2.69
Free Cholesterol:Tot. Lipids	CholC:L-IDL	17.94	1.00	18.03	1.30	17.93	0.92	17.91	1.14	17.93	0.96	17.96	1.21
Triglicerydes:Tot. Lipids in IDL %	Tryg:L-IDL	8.32	2.14	7.84	2.60	7.71	1.82	7.45	2.05	8.00	2.00	7.65	2.41

Table S1 continued		Mean	SD	Median	IQR	Mean	SD	Median	IQR	Mean	SD	Median	IQR
**LDL % - Large**													
Phospholipids:Tot. Lipids	P:L-L LDL	22.71	0.64	22.70	0.91	22.56	0.78	22.55	1.11	22.64	0.72	22.65	0.99
Cholesterol:Tot. Lipids	C:L-L LDL	71.12	1.44	71.22	1.88	71.52	1.34	71.61	1.79	71.33	1.41	71.45	1.85
Cholesteryl Esters:Tot. Lipids	CE:L-L LDL	52.65	0.99	52.76	1.40	52.61	1.07	52.69	1.56	52.63	1.04	52.74	1.48
Free Cholesterol:Tot. Lipids	CholC:L-L LDL	18.49	1.10	18.64	1.45	18.93	1.05	19.09	1.32	18.72	1.09	18.88	1.45
Triglicerydes:Tot. Lipids in Large LDL %	Tryg:L-L LDL	6.14	1.43	5.91	1.85	5.89	1.25	5.73	1.44	6.01	1.34	5.81	1.63
**ILDL % - Medium**													
Phospholipids:Tot. Lipids	P:L-M LDL	26.11	0.75	26.16	1.02	26.02	0.76	26.04	1.00	26.06	0.76	26.10	1.01
Cholesterol:Tot. Lipids	C:L-M LDL	68.66	1.30	68.87	1.57	68.84	1.27	69.02	1.45	68.76	1.29	68.95	1.48
Cholesteryl Esters:Tot. Lipids	CE:L-M LDL	49.51	1.64	49.54	1.93	49.19	1.68	49.24	2.26	49.34	1.67	49.39	2.09
Free Cholesterol:Tot. Lipids	CholC:L-M LDL	19.13	1.68	19.23	2.04	19.66	1.55	19.77	1.97	19.40	1.63	19.53	2.12
Triglicerydes:Tot. Lipids	Tryg:L-M LDL	5.24	1.27	5.03	1.56	5.14	1.18	4.91	1.38	5.19	1.23	4.94	1.48
**LDL % - Small**													
Phospholipids:Tot. Lipids	P:L-S LDL	30.97	1.49	30.82	2.01	31.29	1.64	31.26	2.23	31.14	1.58	31.06	2.08
Cholesterol:Tot. Lipids	C:L-S LDL	63.54	1.67	63.73	2.09	63.53	1.68	63.62	2.10	63.54	1.68	63.69	2.10
Cholesteryl Esters:Tot. Lipids	CE:L-S LDL	46.12	1.81	46.19	2.37	45.84	1.86	45.87	2.41	45.97	1.84	45.99	2.40
Free Cholesterol:Tot. Lipids	CholC:L-S LDL	17.40	1.74	17.68	2.11	17.66	1.56	17.90	1.96	17.54	1.65	17.83	2.00
Triglicerydes:Tot. Lipids	Tryg:L-S LDL	5.49	1.56	5.18	1.86	5.18	1.43	4.92	1.66	5.33	1.50	5.00	1.74
**HDL % - Very Large**													
Phospholipids:Tot. Lipids	P:L-XL HDL	42.90	5.45	43.87	7.17	47.06	4.55	48.25	5.47	45.08	5.41	46.14	7.20
Cholesterol:Tot. Lipids	C:L-XL HDL	51.56	4.25	50.82	5.59	48.69	3.35	48.06	3.84	50.05	4.06	49.27	5.22
Cholesteryl Esters:Tot. Lipids	CE:L-XL HDL	35.09	2.87	34.94	3.53	34.86	2.31	34.81	2.35	34.97	2.59	34.87	2.85
Free Cholesterol:Tot. Lipids	CholC:L-XL HDL	16.50	3.01	16.32	3.76	13.83	2.43	13.33	2.97	15.10	3.03	14.68	4.30
Triglicerydes:Tot. Lipids	Tryg:L-XL HDL	5.38	2.67	4.85	3.10	4.22	2.23	3.49	2.43	4.77	2.52	4.14	2.91
**HDL % - Large**													
Phospholipids:Tot. Lipids	P:L-L HDL	50.59	2.74	50.17	3.65	49.71	2.25	49.12	2.90	50.12	2.54	49.62	3.33
Cholesterol:Tot. Lipids	C:L-L HDL	43.46	4.82	44.31	6.30	45.59	4.06	46.61	4.84	44.58	4.56	45.44	5.69
Cholesteryl Esters:Tot. Lipids	CE:L-L HDL	33.72	4.33	34.47	5.79	35.20	3.62	36.09	4.35	34.50	4.04	35.31	4.87
Free Cholesterol:Tot. Lipids	CholC:L-L HDL	9.78	1.00	9.93	1.20	10.39	0.74	10.53	0.81	10.10	0.92	10.29	1.08
Triglicerydes:Tot. Lipids	Tryg:L-L HDL	5.98	2.87	5.39	3.78	4.70	2.24	4.15	2.47	5.31	2.64	4.62	3.21
**HDL % - Medium**													
Phospholipids:Tot. Lipids	P:L-M HDL	47.40	1.21	47.32	1.73	46.83	1.10	46.67	1.43	47.10	1.19	46.95	1.56
Cholesterol:Tot. Lipids	C:L-M HDL	46.98	2.78	47.23	3.78	48.17	2.61	48.43	3.26	47.61	2.75	47.84	3.67
Cholesteryl Esters:Tot. Lipids	CE:L-M HDL	39.00	2.49	39.26	3.27	39.61	2.27	39.91	2.78	39.32	2.40	39.58	3.08
Free Cholesterol:Tot. Lipids	CholC:L-M HDL	7.98	0.64	7.99	0.83	8.57	0.58	8.54	0.79	8.29	0.67	8.30	0.90
Triglicerydes:Tot. Lipids	Tryg:L-M HDL	5.62	1.69	5.47	2.25	5.00	1.61	4.89	2.07	5.29	1.68	5.17	2.21

Table S1 continued		Mean	SD	Median	IQR	Mean	SD	Median	IQR	Mean	SD	Median	IQR
**HDL % - Small**													
Phospholipids:Tot. Lipids	P:L-S HDL	56.65	1.10	56.67	1.55	57.40	1.09	57.36	1.56	57.04	1.16	57.01	1.57
Cholesterol:Tot. Lipids	C:L-S HDL	38.58	1.72	38.57	2.38	38.35	1.66	38.39	2.33	38.46	1.69	38.52	2.33
Cholesteryl Esters:Tot. Lipids	CE:L-S HDL	28.83	1.71	28.88	2.25	28.32	1.67	28.41	2.30	28.56	1.70	28.65	2.26
Free Cholesterol:Tot. Lipids	CholC:L-S HDL	9.75	0.41	9.72	0.55	10.03	0.52	9.94	0.67	9.90	0.49	9.85	0.61
Triglicerydes:Tot. Lipids	Tryg:L-S HDL	4.77	1.16	4.67	1.49	4.25	1.17	4.18	1.46	4.50	1.19	4.45	1.54

Figure S3: Levels of MRI−measured proton density liver fat fraction (PDLFF) by baseline levels of NMR−measured lipids

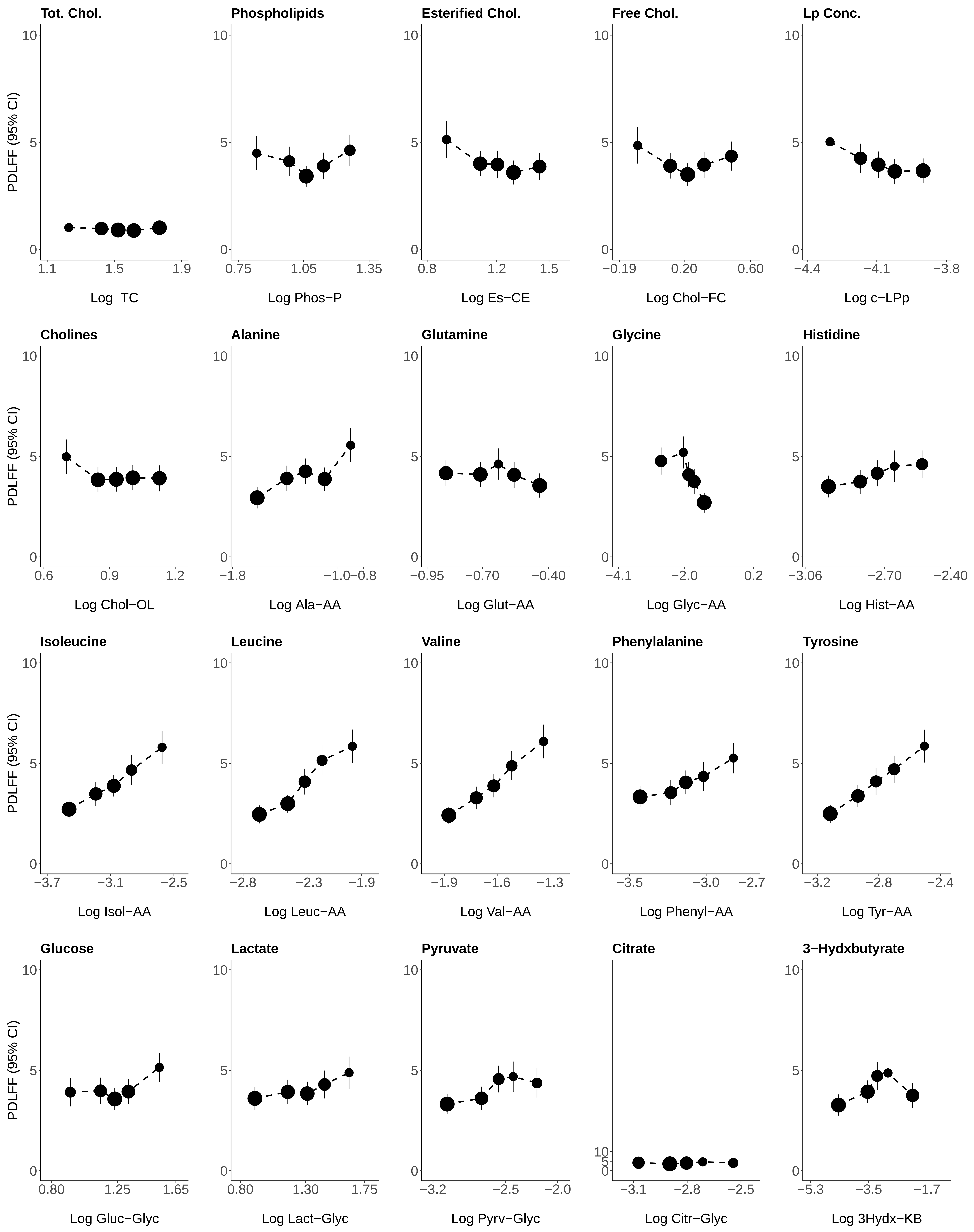

Analyses as per Figure 1.

Figure S3 Continued: Levels of MRI−measured proton density liver fat fraction by baseline levels of NMR−measured lipids

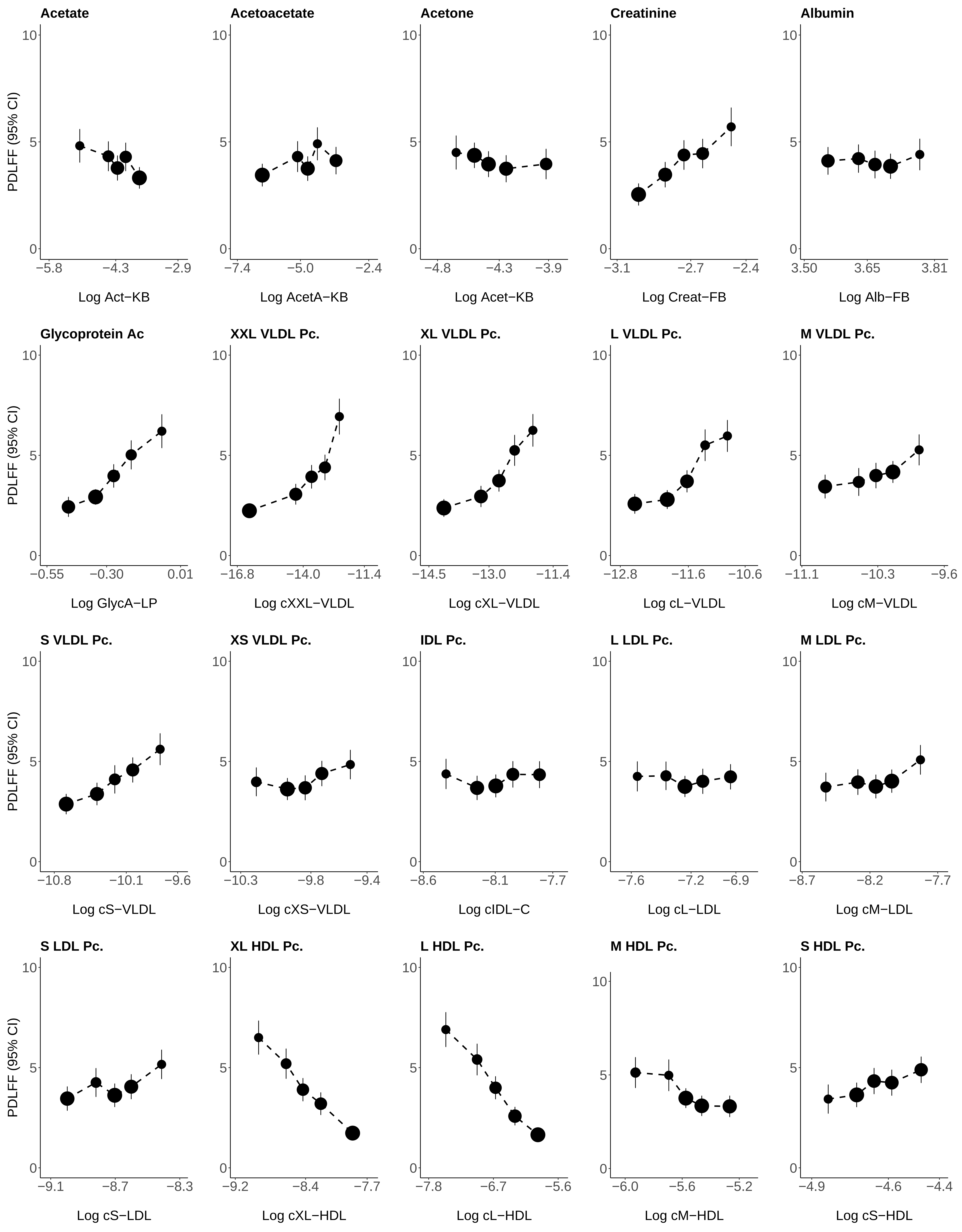

Analyses as per Figure 1.

Figure S4. NMR metabolites and lifer fat by age, sex, and smoking

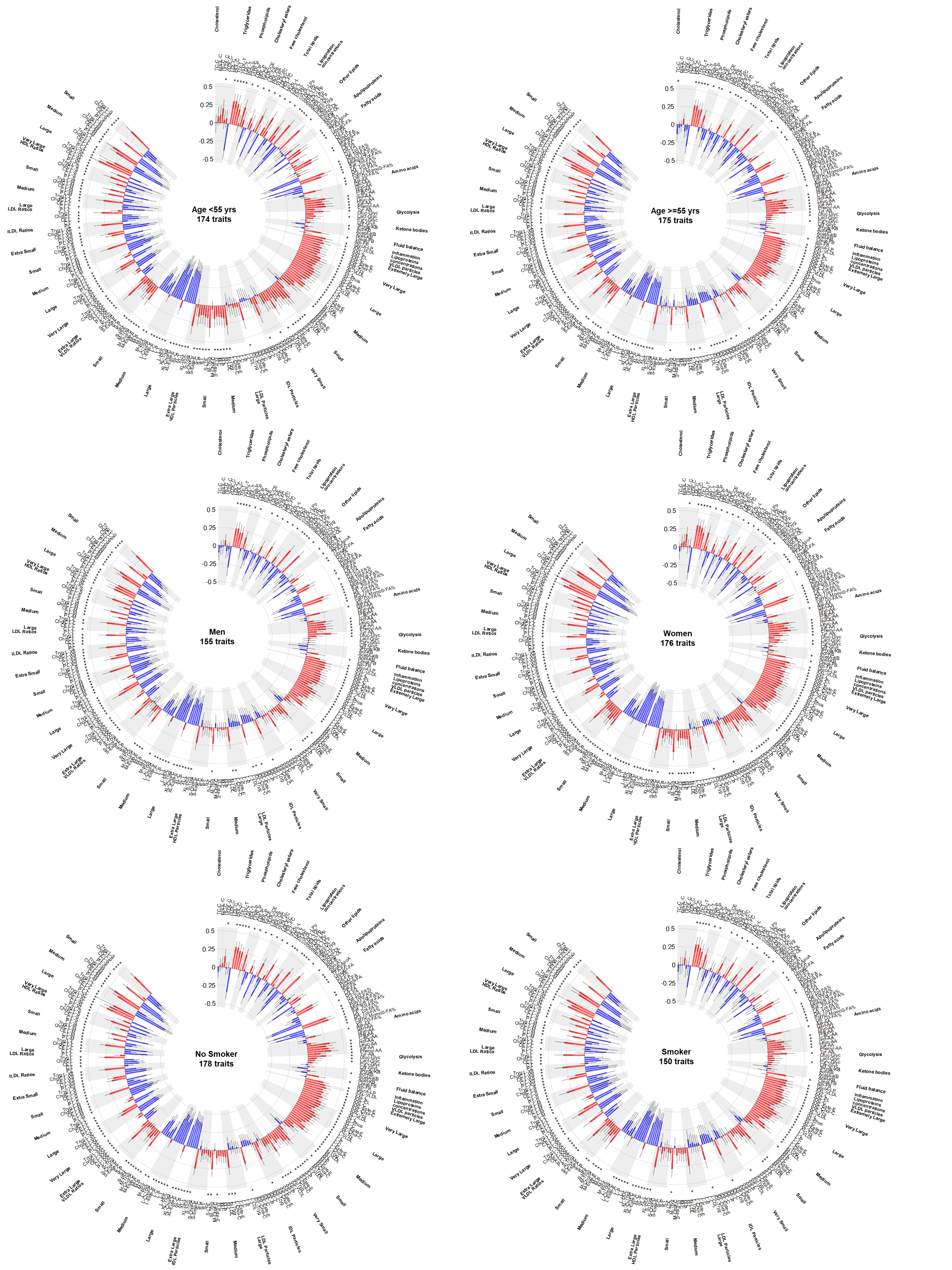

Analyses as per Figure 2

Table S2: Associations of log-levels of NMR metabolites (per 1 SD) with log- levels of liver fat (1 SD) , by diabetes

		No chronic disease		No Diabetes		Diabetes		All	
**Metabolites**	**Label**	**β-coefficient (95% CIs)**	**p-value**	**β-coefficient (95% CIs)**	**p-value**	**β-coefficient (95% CIs)**	**p-value**	**β-coefficient (95% CIs)**	**p-value**
**Cholesterol (mmol/L)**									
Total Cholesterol	TC-C	0.055 (-0.038-0.148)	0.249	-0.040 (-0.124-0.043)	0.343	-0.240 (-0.645-0.165)	0.242	-0.079 (-0.155--0.003)	0.041
Non HDL-C	nHDL-C	0.139 (0.050-0.227)	0.002	0.065 (-0.017-0.146)	0.120	-0.095 (-0.477-0.287)	0.621	0.013 (-0.059-0.084)	0.729
Remnant Cholesterol	RC-C	0.167 (0.078-0.256)	<0.0001	0.091 (0.008-0.174)	0.032	-0.041 (-0.396-0.313)	0.817	0.030 (-0.040-0.101)	0.400
VLDL Cholesterol	VLDL-C	0.205 (0.122-0.289)	<0.00001	0.166 (0.090-0.243)	<0.0001	0.223 (-0.070-0.516)	0.134	0.132 (0.067-0.197)	<0.0001
Clinical LDL Cholesterol	cLDL-C	0.094 (0.005-0.182)	0.038	0.023 (-0.057-0.102)	0.577	-0.181 (-0.561-0.199)	0.346	-0.029 (-0.100-0.042)	0.428
LDL Cholesterol	LDL-C	0.105 (0.017-0.193)	0.019	0.037 (-0.041-0.116)	0.350	-0.144 (-0.547-0.259)	0.479	-0.005 (-0.076-0.066)	0.884
HDL Cholesterol	HDL-C	-0.253 (-0.342--0.163)	<0.00001	-0.313 (-0.384--0.241)	<0.00001	-0.673 (-1.117--0.23)	0.003	-0.326 (-0.401--0.250)	<0.00001
**Triglycerides (mmol/L)**									
Total Triglycerides	Try-T	0.298 (0.213-0.382)	<0.00001	0.275 (0.210-0.339)	<0.00001	0.551 (0.254-0.848)	<0.0001	0.297 (0.233-0.361)	<0.00001
Triglycerides in VLDL	VLDL-T	0.293 (0.210-0.376)	<0.00001	0.275 (0.213-0.337)	<0.00001	0.584 (0.288-0.880)	<0.0001	0.301 (0.239-0.363)	<0.00001
Triglycerides in LDL	LDL-T	0.295 (0.201-0.388)	<0.00001	0.247 (0.166-0.329)	<0.00001	0.400 (0.072-0.727)	0.017	0.260 (0.183-0.337)	<0.00001
Triglycerides in HDL	HDL-T	0.238 (0.160-0.316)	<0.00001	0.215 (0.154-0.275)	<0.00001	0.478 (0.144-0.812)	0.006	0.234 (0.171-0.298)	<0.00001
**Phospholipids (mmol/L)**									
Total Phospholipids in Lipoproteins	Phos-P	0.117 (0.028-0.206)	0.010	0.010 (-0.073-0.093)	0.808	-0.089 (-0.525-0.346)	0.684	-0.018 (-0.098-0.062)	0.661
Phospholipids in VLDL	VLDL-P	0.253 (0.170-0.337)	<0.00001	0.224 (0.152-0.295)	<0.00001	0.416 (0.132-0.701)	0.005	0.216 (0.151-0.281)	<0.00001
Phospholipids in LDL	LDL-P	0.132 (0.045-0.218)	0.003	0.064 (-0.012-0.141)	0.099	-0.101 (-0.500-0.297)	0.614	0.019 (-0.051-0.089)	0.596
Phospholipids in HDL	HDL-P	-0.103 (-0.177--0.029)	0.007	-0.182 (-0.251--0.113)	<0.00001	-0.358 (-0.868-0.152)	0.167	-0.186 (-0.261--0.110)	<0.00001
**Cholesteryl esters (mmol/L)**									
Total Esterified Cholesterol	Es-CE	0.038 (-0.054-0.131)	0.415	-0.057 (-0.139-0.026)	0.177	-0.264 (-0.672-0.144)	0.201	-0.093 (-0.170--0.017)	0.017
Cholesteryl Esters in VLDL	VLDL-CE	0.177 (0.094-0.261)	<0.00001	0.133 (0.054-0.211)	0.001	0.107 (-0.198-0.413)	0.487	0.086 (0.020-0.152)	0.010
Cholesteryl Esters in LDL	LDL-CE	0.126 (0.039-0.214)	0.005	0.060 (-0.019-0.139)	0.138	-0.090 (-0.492-0.312)	0.657	0.020 (-0.051-0.091)	0.582
Cholesteryl Esters in HDL	HDL-CE	-0.267 (-0.36--0.175)	<0.00001	-0.322 (-0.394--0.249)	<0.00001	-0.679 (-1.109--0.248)	0.002	-0.335 (-0.410--0.260)	<0.00001
**Free cholesterol (mmol/L)**									
Total Free Cholesterol	Chol-FC	0.092 (-0.002-0.186)	0.056	0.001 (-0.084-0.086)	0.983	-0.175 (-0.568-0.218)	0.379	-0.043 (-0.118-0.032)	0.258
Free Cholesterol in VLDL	VLDL-FC	0.236 (0.153-0.319)	<0.00001	0.205 (0.132-0.278)	<0.00001	0.359 (0.074-0.644)	0.014	0.188 (0.124-0.253)	<0.00001
Free Cholesterol in LDL	LDL-FC	0.037 (-0.051-0.126)	0.409	-0.028 (-0.104-0.048)	0.466	-0.273 (-0.659-0.112)	0.162	-0.075 (-0.146--0.003)	0.041
Free Cholesterol in HDL	HDL-FC	-0.184 (-0.263--0.105)	<0.00001	-0.263 (-0.332--0.193)	<0.00001	-0.604 (-1.088--0.119)	0.015	-0.275 (-0.351--0.199)	<0.00001

Table S2 Continued

**Total lipids (mmol/L)**									
Total Lipids in Lipoprotein Particles	Lp-L	0.182 (0.091-0.273)	<0.00001	0.093 (0.010-0.177)	0.028	0.031 (-0.356-0.418)	0.872	0.059 (-0.016-0.134)	0.124
Total Lipids in VLDL	VLDL-L	0.270 (0.187-0.354)	<0.00001	0.245 (0.177-0.313)	<0.00001	0.470 (0.185-0.754)	0.002	0.249 (0.184-0.313)	<0.00001
Total Lipids in LDL	LDL-L	0.129 (0.042-0.216)	0.004	0.061 (-0.018-0.140)	0.129	-0.101 (-0.506-0.304)	0.620	0.019 (-0.053-0.090)	0.608
Total Lipids in HDL	HDL-L	-0.152 (-0.23--0.075)	<0.0001	-0.228 (-0.298--0.159)	<0.00001	-0.478 (-0.974-0.018)	0.058	-0.236 (-0.311--0.160)	<0.00001
**Lipoprotein Conc. (mmol/L)**									
Total Conc. of Lipoprotein Particles	c-LPp	-0.017 (-0.095-0.060)	0.663	-0.103 (-0.179--0.028)	0.008	-0.249 (-0.727-0.228)	0.302	-0.112 (-0.190--0.034)	0.005
Conc. of VLDL Particles	cVLDL-LPp	0.224 (0.139-0.310)	<0.00001	0.190 (0.114-0.267)	<0.00001	0.306 (0.001-0.612)	0.049	0.170 (0.101-0.238)	<0.00001
Conc. of LDL Particles	cLDL-LPp	0.129 (0.038-0.219)	0.005	0.074 (-0.006-0.154)	0.069	-0.073 (-0.436-0.29)	0.689	0.028 (-0.041-0.096)	0.431
Conc. of HDL Particles	cHDL-LPp	-0.046 (-0.123-0.031)	0.244	-0.124 (-0.198--0.049)	0.001	-0.267 (-0.752-0.218)	0.277	-0.127 (-0.204--0.050)	0.001
Average Diameter for VLDL Particles	dVLDL-LPp	0.293 (0.214-0.372)	<0.00001	0.281 (0.223-0.338)	<0.00001	0.668 (0.344-0.991)	<0.0001	0.323 (0.261-0.385)	<0.00001
Average Diameter for LDL Particles	dLDL-LPp	-0.165 (-0.245--0.085)	<0.0001	-0.203 (-0.271--0.135)	<0.00001	-0.624 (-0.970--0.278)	0.001	-0.251 (-0.32--0.183)	<0.00001
Average Diameter for HDL Particles	dHDL-LPp	-0.298 (-0.375--0.221)	<0.00001	-0.338 (-0.397--0.278)	<0.00001	-0.779 (-1.160--0.397)	<0.0001	-0.367 (-0.43--0.304)	<0.00001
**Other lipids (mmol/L)**									
Phosphoglycerides	Pglyc-OL	0.109 (0.025-0.193)	0.011	0.001 (-0.079-0.082)	0.971	-0.050 (-0.536-0.437)	0.839	-0.013 (-0.095-0.068)	0.748
Phosphoglycerides:Tryglicerides Ratio	Pglyc:Tryg	0.308 (0.221-0.394)	<0.00001	0.318 (0.255-0.382)	<0.00001	0.716 (0.381-1.051)	<0.0001	0.352 (0.287-0.417)	<0.00001
Total Cholines	Chol-OL	0.065 (-0.019-0.148)	0.129	-0.044 (-0.125-0.036)	0.281	-0.141 (-0.619-0.337)	0.558	-0.062 (-0.142-0.018)	0.131
Phosphatidylcholines	Pdchol-OL	0.058 (-0.026-0.143)	0.174	-0.049 (-0.130-0.032)	0.235	-0.119 (-0.624-0.386)	0.642	-0.061 (-0.144-0.022)	0.152
Sphingomyelins	Spyng-OL	0.013 (-0.07-0.096)	0.761	-0.076 (-0.152-0.001)	0.053	-0.390 (-0.820-0.040)	0.075	-0.113 (-0.187--0.039)	0.003
**Apolipoproteins (gl/L)**									
Apolipoprotein B	Apo-B	0.149 (0.06-0.239)	0.001	0.090 (0.010-0.170)	0.028	-0.038 (-0.400-0.324)	0.836	0.039 (-0.030-0.108)	0.263
Apolipoprotein A1	Apo-A1	-0.101 (-0.176--0.025)	0.009	-0.175 (-0.246--0.104)	<0.00001	-0.359 (-0.854-0.136)	0.153	-0.179 (-0.255--0.104)	<0.00001
ApoB:ApoA Ratio	ApoB:ApoA	0.169 (0.086-0.252)	<0.0001	0.164 (0.094-0.234)	<0.00001	0.158 (-0.185-0.501)	0.363	0.125 (0.061-0.190)	<0.0001
**Fatty acids (mmol/L)**									
Total Fatty Acids	TFA-FA	0.252 (0.164-0.340)	<0.00001	0.175 (0.100-0.251)	<0.00001	0.293 (-0.061-0.648)	0.103	0.173 (0.100-0.246)	<0.00001
Degree of Unsaturation	UnsatD-FA	-0.244 (-0.347--0.141)	<0.00001	-0.261 (-0.334--0.189)	<0.00001	-0.527 (-0.844--0.209)	0.001	-0.315 (-0.388--0.243)	<0.00001
Omega-3 FA	O3-FA	0.097 (0.003-0.190)	0.042	0.048 (-0.026-0.123)	0.203	-0.12 (-0.516-0.276)	0.548	0.024 (-0.051-0.099)	0.537
Omega-6 FA	O6-FA	0.089 (0.001-0.177)	0.049	-0.016 (-0.093-0.062)	0.695	-0.002 (-0.403-0.399)	0.991	-0.038 (-0.109-0.033)	0.298
Polyunsaturated FA	PolyU-FA	0.106 (0.009-0.203)	0.032	0.003 (-0.078-0.084)	0.942	-0.022 (-0.446-0.403)	0.919	-0.025 (-0.100-0.050)	0.507
Monounsaturated FA	MomoS-FA	0.292 (0.209-0.376)	<0.00001	0.236 (0.167-0.304)	<0.00001	0.462 (0.143-0.780)	0.005	0.259 (0.191-0.326)	<0.00001
Saturated FA	Sat-FA	0.304 (0.216-0.392)	<0.00001	0.236 (0.162-0.310)	<0.00001	0.305 (-0.025-0.635)	0.070	0.233 (0.160-0.306)	<0.00001
Linoleic FA	LA-FA	0.038 (-0.055-0.131)	0.418	-0.060 (-0.141-0.020)	0.142	-0.015 (-0.372-0.342)	0.933	-0.071 (-0.141--0.002)	0.044

Table S2 Continued

Docosahexaenoic FA	DA-FA	-0.015 (-0.120-0.089)	0.772	-0.065 (-0.146-0.017)	0.119	-0.394 (-0.712--0.076)	0.016	-0.107 (-0.183--0.032)	0.005
Omega-3: Total FA %	O3:Tot-FA%	-0.003 (-0.090-0.084)	0.946	-0.028 (-0.099-0.042)	0.429	-0.330 (-0.604--0.056)	0.019	-0.068 (-0.136-0.001)	0.053
Omega-6: Total FA %	O6:Tot-FA%	-0.394 (-0.498--0.290)	<0.00001	-0.361 (-0.436--0.285)	<0.00001	-0.458 (-0.761--0.155)	0.003	-0.391 (-0.465--0.317)	<0.00001
Polyunsaturated:Total FA %	PolyU:Tot-FA%	-0.412 (-0.513--0.312)	<0.00001	-0.366 (-0.438--0.295)	<0.00001	-0.488 (-0.787--0.188)	0.002	-0.401 (-0.473--0.328)	<0.00001
Monounsaturated: Total FA %	MomoS:Tot-FA%	0.314 (0.230-0.399)	<0.00001	0.298 (0.236-0.361)	<0.00001	0.683 (0.365-1.001)	<0.0001	0.348 (0.282-0.414)	<0.00001
Saturated : Total FA%	Sat: Tot-FA%	0.311 (0.227-0.396)	<0.00001	0.294 (0.230-0.359)	<0.00001	0.356 (0.030-0.683)	0.033	0.311 (0.245-0.377)	<0.00001
Linoleic: Total FA%	LA: Tot-FA%	-0.386 (-0.495--0.278)	<0.00001	-0.377 (-0.454--0.299)	<0.00001	-0.444 (-0.761--0.127)	0.007	-0.389 (-0.462--0.315)	<0.00001
Docosahexaenoic : Total FA%	DA: Tot-FA%	-0.148 (-0.239--0.058)	0.001	-0.154 (-0.225--0.083)	<0.0001	-0.476 (-0.760--0.192)	0.001	-0.197 (-0.265--0.129)	<0.00001
Polyunsaturated: Monounsaturated FA %	PolyU:MomoS-FA%	-0.373 (-0.465--0.280)	<0.00001	-0.342 (-0.409--0.276)	<0.00001	-0.590 (-0.895--0.285)	<0.001	-0.386 (-0.455--0.318)	<0.00001
Omega-6:Omega-3 FA %	O6: O3-FA%	-0.079 (-0.17-0.012)	0.089	-0.058 (-0.129-0.014)	0.113	0.120 (-0.232-0.472)	0.498	-0.040 (-0.111-0.031)	0.268
**Amino acids (mmol/L)**									
Alanine	Ala-AA	0.151 (0.084-0.218)	0.000	0.140 (0.080-0.199)	<0.00001	0.465 (0.027-0.902)	0.038	0.181 (0.114-0.249)	<0.00001
Glutamine	Glut-AA	-0.033 (-0.106-0.040)	0.378	-0.043 (-0.105-0.02)	0.180	-0.272 (-0.520--0.024)	0.032	-0.049 (-0.104-0.006)	0.083
Glycine	Glyc-AA	-0.005 (-0.043-0.034)	0.806	-0.059 (-0.120-0.003)	0.061	0.001 (-0.200-0.202)	0.993	-0.032 (-0.083-0.018)	0.207
Histidine	Hist-AA	0.064 (-0.001-0.128)	0.053	0.061 (0.008-0.114)	0.024	0.274 (-0.023-0.571)	0.070	0.083 (0.028-0.139)	0.003
Tot. Conc. Branched-Chain AA	C-BC-AA	0.257 (0.178-0.336)	<0.00001	0.229 (0.166-0.292)	<0.00001	0.651 (0.351-0.951)	<0.0001	0.282 (0.215-0.349)	<0.00001
Isoleucine	Isol-AA	0.208 (0.135-0.281)	<0.00001	0.191 (0.130-0.252)	<0.00001	0.580 (0.244-0.915)	0.001	0.232 (0.167-0.297)	<0.00001
Leucine	Leuc-AA	0.234 (0.154-0.314)	<0.00001	0.219 (0.157-0.282)	<0.00001	0.613 (0.288-0.938)	<0.0001	0.264 (0.198-0.331)	<0.00001
Valine	Val-AA	0.267 (0.188-0.346)	<0.00001	0.228 (0.166-0.290)	<0.00001	0.679 (0.389-0.968)	<0.0001	0.286 (0.220-0.353)	<0.00001
Phenylalanine	Phenyl-AA	0.121 (0.056-0.186)	<0.0001	0.112 (0.048-0.176)	0.001	0.302 (0.023-0.581)	0.034	0.138 (0.076-0.200)	<0.0001
Tyrosine	Tyr-AA	0.224 (0.147-0.301)	<0.00001	0.198 (0.136-0.260)	<0.00001	0.610 (0.353-0.868)	<0.0001	0.252 (0.187-0.317)	<0.00001
**Glycolysis (mmol/L)**									
Glucose	Gluc-Glyc	0.096 (0.023-0.169)	0.010	0.073 (0.010-0.135)	0.023	0.122 (-0.131-0.376)	0.339	0.096 (0.032-0.160)	0.004
Lactate	Lact-Glyc	0.108 (0.025-0.191)	0.011	0.062 (-0.003-0.126)	0.060	0.293 (-0.125-0.712)	0.167	0.096 (0.031-0.162)	0.004
Pyruvate	Pyrv-Glyc	0.148 (0.076-0.220)	<0.0001	0.072 (0.013-0.131)	0.017	0.336 (0.016-0.657)	0.040	0.081 (0.025-0.137)	0.005
Citrate	Citr-Glyc	0.035 (-0.042-0.113)	0.372	0.011 (-0.055-0.077)	0.749	0.023 (-0.262-0.309)	0.871	0.013 (-0.050-0.076)	0.684
**Ketone bodies (mmol/L)**									
3-Hydroxybutyrate	3Hydx-KB	0.038 (-0.015-0.091)	0.164	0.023 (-0.030-0.075)	0.392	-0.066 (-0.283-0.15)	0.544	0.036 (-0.018-0.089)	0.194
Acetate	Act-KB	-0.110 (-0.190--0.029)	0.007	-0.095 (-0.161--0.029)	0.005	-0.810 (-1.407--0.213)	0.008	-0.101 (-0.187--0.015)	0.021
Acetoacetate	AcetA-KB	0.061 (0.007-0.115)	0.028	0.064 (0.017-0.112)	0.008	0.095 (-0.064-0.253)	0.238	0.039 (-0.022-0.101)	0.208
Acetone	Acet-KB	-0.029 (-0.100-0.041)	0.419	-0.043 (-0.105-0.019)	0.175	-0.252 (-0.616-0.112)	0.172	-0.055 (-0.123-0.013)	0.110

Table S2 Continued

**Fluid balance (mmol/L)**									
Creatinine	Creat-FB	0.191 (0.105-0.277)	<0.00001	0.175 (0.101-0.249)	<0.00001	0.561 (0.161-0.960)	0.007	0.213 (0.139-0.286)	<0.00001
Albumin	Alb-FB	0.020 (-0.038-0.078)	0.501	0.001 (-0.054-0.057)	0.963	-0.084 (-0.421-0.252)	0.620	-0.003 (-0.058-0.052)	0.921
**Inflammation (mmol/L)**									
Glycoprotein Acetyls	GlycA-LP	0.298 (0.226-0.371)	<0.00001	0.247 (0.188-0.306)	<0.00001	0.469 (0.172-0.766)	0.002	0.271 (0.210-0.331)	<0.00001
**Lipoproteins Conc.**									
**VLDL particles, Extremely Large**									
Conc. of Extremely Large VLDL Particles	XXL-VLDL	0.234 (0.165-0.303)	<0.00001	0.239 (0.183-0.295)	<0.00001	0.594 (0.304-0.885)	<0.0001	0.282 (0.226-0.339)	<0.00001
Total Lipids in Extremely Large VLDL	XXL-L	0.268 (0.193-0.343)	<0.00001	0.263 (0.206-0.320)	<0.00001	0.628 (0.336-0.921)	<0.0001	0.302 (0.244-0.360)	<0.00001
Phospholipids in Extremely Large VLDL	XXL-PL	0.201 (0.136-0.266)	<0.00001	0.210 (0.158-0.262)	<0.00001	0.540 (0.225-0.855)	0.001	0.255 (0.200-0.310)	<0.00001
Cholesterol in Extremely Large VLDL	XXL-C	0.251 (0.179-0.322)	<0.00001	0.251 (0.194-0.308)	<0.00001	0.586 (0.285-0.888)	<0.0001	0.287 (0.231-0.344)	<0.00001
Cholesteryl Esters in Extremely Large VLDL	XXL-Este-E	0.246 (0.177-0.315)	<0.00001	0.243 (0.185-0.301)	<0.00001	0.520 (0.223-0.818)	<0.0001	0.273 (0.218-0.329)	<0.00001
Free Cholesterol in Extremely Large VLDL	XXL-Chol-C	0.248 (0.175-0.321)	<0.00001	0.251 (0.194-0.308)	<0.00001	0.630 (0.327-0.932)	<0.0001	0.293 (0.235-0.351)	<0.00001
Triglycerides in Extremely Large VLDL	XXL-Try	0.260 (0.172-0.347)	<0.00001	0.246 (0.183-0.309)	<0.00001	0.682 (0.367-0.996)	<0.0001	0.277 (0.215-0.338)	<0.00001
**Very Large VLDL**									
Conc. of Very Large VLDL Particles	XL-VLDL	0.278 (0.200-0.355)	<0.00001	0.275 (0.216-0.335)	<0.00001	0.599 (0.309-0.889)	<0.0001	0.303 (0.245-0.361)	<0.00001
Total Lipids in Very Large VLDL	XL-L	0.280 (0.202-0.358)	<0.00001	0.276 (0.217-0.336)	<0.00001	0.594 (0.306-0.881)	<0.0001	0.303 (0.245-0.362)	<0.00001
Phospholipids in Very Large VLDL	XL-Phos	0.258 (0.183-0.334)	<0.00001	0.261 (0.201-0.322)	<0.00001	0.553 (0.269-0.837)	<0.0001	0.285 (0.227-0.343)	<0.00001
Cholesterol in Very Large VLDL	XL-C	0.251 (0.175-0.328)	<0.00001	0.242 (0.179-0.304)	<0.00001	0.514 (0.226-0.802)	<0.0001	0.248 (0.190-0.306)	<0.00001
Cholesteryl Esters in Very Large VLDL	XL-Este-E	0.233 (0.158-0.309)	<0.00001	0.219 (0.154-0.284)	<0.00001	0.446 (0.155-0.736)	0.003	0.213 (0.155-0.270)	<0.00001
Free Cholesterol in Very Large VLDL	XL-Chol-C	0.263 (0.186-0.340)	<0.00001	0.259 (0.197-0.320)	<0.00001	0.555 (0.270-0.840)	<0.0001	0.277 (0.218-0.336)	<0.00001
Triglycerides in Very Large VLDL	XL-Try	0.287 (0.209-0.366)	<0.00001	0.286 (0.228-0.344)	<0.00001	0.621 (0.329-0.913)	<0.0001	0.320 (0.262-0.378)	<0.00001
**Large VLDL**									
Conc. of Large VLDL Particles	L-VLDL	0.275 (0.195-0.354)	<0.00001	0.264 (0.202-0.326)	<0.00001	0.574 (0.279-0.869)	<0.0001	0.283 (0.223-0.343)	<0.00001
Total Lipids in Large VLDL	L-L	0.270 (0.190-0.349)	<0.00001	0.259 (0.198-0.320)	<0.00001	0.567 (0.275-0.858)	<0.0001	0.277 (0.217-0.336)	<0.00001
Phospholipids in Large VLDL	L-P	0.259 (0.183-0.335)	<0.00001	0.257 (0.198-0.317)	<0.00001	0.583 (0.286-0.880)	<0.0001	0.278 (0.221-0.335)	<0.00001
Cholesterol in Large VLDL	L-C	0.241 (0.164-0.317)	<0.00001	0.230 (0.168-0.293)	<0.00001	0.511 (0.215-0.808)	0.001	0.234 (0.176-0.293)	<0.00001
Cholesteryl Esters in Large VLDL	L-Este-E	0.210 (0.135-0.286)	<0.00001	0.194 (0.129-0.260)	<0.00001	0.413 (0.118-0.708)	0.007	0.183 (0.124-0.241)	<0.00001
Free Cholesterol in Large VLDL	L-Chol-C	0.266 (0.189-0.344)	<0.00001	0.260 (0.200-0.321)	<0.00001	0.582 (0.285-0.878)	<0.0001	0.278 (0.220-0.336)	<0.00001
Triglycerides in Large VLDL	L-Tryg	0.278 (0.198-0.359)	<0.00001	0.264 (0.205-0.324)	<0.00001	0.583 (0.290-0.877)	<0.0001	0.288 (0.229-0.348)	<0.00001

Table S2 Continued

**Medium VLDL**									
Conc. of Medium VLDL Particles	M-VLDL	0.189 (0.105-0.273)	<0.0001	0.146 (0.068-0.225)	<0.0001	0.200 (-0.105-0.505)	0.196	0.115 (0.049-0.181)	0.001
Total Lipids in Medium VLDL	M-L	0.211 (0.128-0.295)	<0.00001	0.171 (0.095-0.247)	<0.0001	0.283 (-0.019-0.585)	0.066	0.149 (0.083-0.216)	<0.0001
Phospholipids in Medium VLDL	M-P	0.188 (0.102-0.273)	<0.0001	0.134 (0.050-0.218)	0.002	0.164 (-0.139-0.466)	0.284	0.099 (0.030-0.168)	0.005
Cholesterol in Medium VLDL	M-C	0.076 (-0.016-0.169)	0.106	0.010 (-0.080-0.099)	0.834	-0.121 (-0.442-0.199)	0.454	-0.040 (-0.112-0.031)	0.265
Cholesteryl Esters in Medium VLDL	M-Este-E	-0.008 (-0.113-0.098)	0.885	-0.077 (-0.171-0.016)	0.104	-0.146 (-0.408-0.117)	0.274	-0.114 (-0.187--0.04)	0.002
Free Cholesterol in Medium VLDL	M-Chol-C	0.155 (0.068-0.241)	<0.001	0.099 (0.014-0.184)	0.023	0.071 (-0.247-0.389)	0.658	0.057 (-0.012-0.126)	0.106
Triglycerides in Medium VLDL	M-Tryg	0.259 (0.177-0.341)	<0.00001	0.237 (0.172-0.301)	<0.00001	0.512 (0.207-0.817)	0.001	0.249 (0.187-0.311)	<0.00001
**Small VLDL**									
Conc. of Small VLDL Particles	S-VLDL	0.222 (0.137-0.306)	<0.00001	0.200 (0.127-0.272)	<0.00001	0.394 (0.075-0.713)	0.016	0.194 (0.127-0.261)	<0.00001
Total Lipids in Small VLDL	S-L	0.225 (0.141-0.309)	<0.00001	0.197 (0.123-0.270)	<0.00001	0.371 (0.047-0.694)	0.025	0.189 (0.121-0.256)	<0.00001
Phospholipids in Small VLDL	S-P	0.188 (0.103-0.273)	<0.0001	0.145 (0.065-0.224)	<0.0001	0.196 (-0.146-0.538)	0.259	0.117 (0.048-0.186)	0.001
Cholesterol in Small VLDL	S-C	0.176 (0.093-0.259)	<0.0001	0.140 (0.064-0.217)	<0.0001	0.144 (-0.199-0.486)	0.406	0.106 (0.039-0.172)	0.002
Cholesteryl Esters in Small VLDL	S-Este-E	0.189 (0.107-0.270)	<0.0001	0.162 (0.089-0.235)	<0.0001	0.208 (-0.126-0.543)	0.219	0.133 (0.069-0.197)	<0.0001
Free Cholesterol in Small VLDL	S-Chol-C	0.147 (0.060-0.234)	0.001	0.095 (0.013-0.177)	0.023	0.035 (-0.321-0.391)	0.845	0.054 (-0.016-0.123)	0.131
Triglycerides in Small VLDL	S-Tryg	0.250 (0.167-0.332)	<0.00001	0.235 (0.171-0.298)	<0.00001	0.538 (0.226-0.851)	0.001	0.258 (0.194-0.322)	<0.00001
**Very Small VLDL**									
Conc. of Very Small VLDL Particles	XS-VLDL	0.163 (0.072-0.253)	<0.001	0.117 (0.035-0.199)	0.005	0.055 (-0.307-0.417)	0.763	0.072 (-0.002-0.145)	0.055
Total Lipids in Very Small VLDL	XS-L	0.158 (0.067-0.249)	<0.001	0.109 (0.027-0.191)	0.010	0.049 (-0.311-0.409)	0.787	0.065 (-0.009-0.138)	0.085
Phospholipids in Very Small VLDL	XS-P	0.164 (0.072-0.255)	<0.001	0.119 (0.038-0.201)	0.004	0.112 (-0.226-0.450)	0.511	0.083 (0.011-0.155)	0.024
Cholesterol in Very Small VLDL	XS-C	0.080 (-0.012-0.172)	0.086	0.021 (-0.062-0.104)	0.618	-0.173 (-0.537-0.191)	0.347	-0.041 (-0.115-0.033)	0.276
Cholesteryl Esters in Very Small VLDL	XS-Este-E	0.050 (-0.042-0.141)	0.285	-0.013 (-0.095-0.070)	0.762	-0.219 (-0.576-0.139)	0.227	-0.074 (-0.149-0.001)	0.053
Free Cholesterol in Very Small VLDL	XS-Chol-C	0.135 (0.044-0.226)	0.004	0.088 (0.006-0.170)	0.036	-0.024 (-0.378-0.330)	0.893	0.036 (-0.037-0.108)	0.331
Triglycerides in Very Small VLDL	XS-Tryg	0.245 (0.156-0.333)	<0.00001	0.224 (0.153-0.296)	<0.00001	0.445 (0.140-0.750)	0.005	0.243 (0.173-0.312)	<0.00001
**IDL Particles**									
Conc. of IDL Particles	IDL-C	0.143 (0.060-0.226)	<0.001	0.060 (-0.014-0.135)	0.114	-0.092 (-0.458-0.275)	0.621	-0.004 (-0.074-0.066)	0.913
Total Lipids in IDL	IDL-L	0.081 (-0.009-0.172)	0.079	-0.016 (-0.098-0.067)	0.711	-0.240 (-0.631-0.151)	0.225	-0.073 (-0.150-0.004)	0.063
Phospholipids in IDL	IDL-P	0.046 (-0.047-0.139)	0.331	-0.034 (-0.117-0.048)	0.414	-0.259 (-0.647-0.129)	0.188	-0.086 (-0.162--0.010)	0.027
Cholesterol in IDL	IDL-C	0.052 (-0.039-0.142)	0.266	-0.048 (-0.128-0.033)	0.246	-0.268 (-0.639-0.102)	0.154	-0.102 (-0.179--0.025)	0.010
Cholesteryl Esters in IDL	IDL-Este-E	0.058 (-0.032-0.147)	0.208	-0.049 (-0.130-0.032)	0.233	-0.258 (-0.634-0.117)	0.174	-0.102 (-0.179--0.024)	0.010
Free Cholesterol in IDL	IDL-Chol-C	0.032 (-0.061-0.125)	0.495	-0.043 (-0.122-0.037)	0.295	-0.298 (-0.657-0.061)	0.103	-0.100 (-0.174--0.025)	0.009
Triglycerides in IDL	IDL-Tryg	0.254 (0.161-0.348)	<0.00001	0.217 (0.138-0.296)	<0.00001	0.357 (0.021-0.693)	0.037	0.225 (0.150-0.300)	<0.00001

Table S2 Continued

**LDL Particles, Large**									
Conc. of Large LDL Particles	L-LDL	0.095 (0.005-0.186)	0.038	0.033 (-0.047-0.113)	0.419	-0.169 (-0.525-0.186)	0.346	-0.013 (-0.082-0.055)	0.702
Total Lipids in Large LDL	L-L	0.093 (0.005-0.181)	0.038	0.021 (-0.058-0.100)	0.597	-0.184 (-0.595-0.228)	0.376	-0.023 (-0.095-0.05)	0.542
Phospholipids in Large LDL	L-P	0.104 (0.017-0.191)	0.019	0.032 (-0.045-0.109)	0.415	-0.167 (-0.564-0.23)	0.405	-0.017 (-0.089-0.054)	0.631
Cholesterol in Large LDL	L-C	0.064 (-0.024-0.152)	0.155	-0.006 (-0.084-0.072)	0.882	-0.225 (-0.632-0.182)	0.274	-0.048 (-0.120-0.025)	0.196
Cholesteryl Esters in Large LDL	L-Este-E	0.079 (-0.009-0.167)	0.080	0.010 (-0.068-0.088)	0.801	-0.189 (-0.602-0.224)	0.366	-0.029 (-0.101-0.044)	0.434
Free Cholesterol in Large LDL	L-Chol-C	0.018 (-0.072-0.108)	0.692	-0.052 (-0.129-0.025)	0.182	-0.308 (-0.687-0.071)	0.110	-0.099 (-0.172--0.026)	0.008
Triglycerides in Large LDL	L-Tryg	0.285 (0.190-0.379)	<0.00001	0.237 (0.154-0.320)	<0.00001	0.364 (0.015-0.714)	0.041	0.246 (0.168-0.324)	<0.00001
**Medium LDL**									
Conc. of Medium LDL Particles	M-LDL	0.162 (0.074-0.249)	<0.001	0.127 (0.051-0.204)	0.001	0.077 (-0.300-0.454)	0.686	0.085 (0.017-0.152)	0.014
Total Lipids in Medium LDL	M-L	0.181 (0.095-0.266)	<0.0001	0.123 (0.046-0.201)	0.002	0.047 (-0.337-0.431)	0.809	0.088 (0.019-0.157)	0.012
Phospholipids in Medium LDL	M-P	0.172 (0.089-0.255)	<0.0001	0.114 (0.039-0.189)	0.003	0.024 (-0.368-0.415)	0.905	0.080 (0.012-0.148)	0.021
Cholesterol in Medium LDL	M-C	0.167 (0.082-0.252)	<0.001	0.111 (0.033-0.188)	0.005	0.015 (-0.368-0.399)	0.936	0.072 (0.003-0.141)	0.040
Cholesteryl Esters in Medium LDL	M-Este-E	0.193 (0.108-0.278)	<0.0001	0.139 (0.061-0.217)	<0.0001	0.092 (-0.278-0.462)	0.622	0.104 (0.036-0.173)	0.003
Free Cholesterol in Medium LDL	M-Chol-C	0.077 (-0.009-0.162)	0.080	0.019 (-0.055-0.093)	0.618	-0.187 (-0.583-0.208)	0.349	-0.024 (-0.093-0.046)	0.504
Triglycerides in Medium LDL	M-Tryg	0.301 (0.208-0.395)	<0.00001	0.254 (0.172-0.335)	<0.0001	0.430 (0.116-0.744)	0.008	0.271 (0.194-0.348)	<0.0001
**Small LDL**									
Conc. of Small LDL Particles	S-LDL	0.172 (0.084-0.260)	<0.0001	0.127 (0.050-0.205)	0.001	0.085 (-0.259-0.429)	0.624	0.089 (0.022-0.157)	0.010
Total Lipids in Small LDL	S-L	0.166 (0.077-0.256)	<0.0001	0.108 (0.030-0.187)	0.007	0.024 (-0.347-0.394)	0.900	0.071 (0.002-0.140)	0.044
Phospholipids in Small LDL	S-P	0.124 (0.034-0.215)	0.007	0.070 (-0.006-0.146)	0.072	-0.055 (-0.449-0.339)	0.781	0.034 (-0.035-0.104)	0.330
Cholesterol in Small LDL	S-C	0.153 (0.066-0.241)	<0.0001	0.095 (0.017-0.172)	0.017	-0.013 (-0.382-0.357)	0.946	0.054 (-0.014-0.122)	0.118
Cholesteryl Esters in Small LDL	S-Este-E	0.187 (0.100-0.274)	<0.001	0.129 (0.050-0.207)	0.001	0.074 (-0.281-0.429)	0.681	0.091 (0.023-0.159)	0.009
Free Cholesterol in Small LDL	S-Chol-C	0.037 (-0.050-0.124)	0.408	-0.012 (-0.084-0.060)	0.740	-0.232 (-0.610-0.146)	0.226	-0.053 (-0.122-0.016)	0.132
Triglycerides in Small LDL	S-Tryg	0.298 (0.208-0.389)	<0.00001	0.261 (0.188-0.334)	<0.00001	0.473 (0.180-0.767)	0.002	0.281 (0.210-0.352)	<0.00001
**HDL Particles, Extra Large**									
Conc. of Very Large HDL Particles	XL-HDL	-0.282 (-0.361--0.204)	<0.00001	-0.331 (-0.395--0.267)	<0.00001	-0.731 (-1.095--0.368)	<0.0001	-0.350 (-0.417--0.282)	<0.00001
Total Lipids in Very Large HDL	XL-L	-0.308 (-0.387--0.228)	<0.00001	-0.348 (-0.412--0.285)	<0.00001	-0.724 (-1.057--0.390)	<0.0001	-0.369 (-0.437--0.301)	<0.00001
Phospholipids in Very Large HDL	XL-P	-0.303 (-0.386--0.220)	<0.00001	-0.342 (-0.407--0.276)	<0.00001	-0.654 (-0.962--0.345)	<0.0001	-0.359 (-0.427--0.290)	<0.00001
Cholesterol in Very Large HDL	XL-C	-0.331 (-0.414--0.248)	<0.00001	-0.365 (-0.429--0.301)	<0.00001	-0.753 (-1.088--0.419)	<0.0001	-0.386 (-0.455--0.318)	<0.00001
Cholesteryl Esters in Very Large HDL	XL-Este-E	-0.358 (-0.446--0.271)	<0.00001	-0.390 (-0.456--0.324)	<0.00001	-0.754 (-1.094--0.415)	<0.0001	-0.405 (-0.477--0.333)	<0.00001

Table S2 Continued

Free Cholesterol in Very Large HDL	XL-Chol-C	-0.233 (-0.304--0.162)	<0.00001	-0.271 (-0.330--0.212)	<0.00001	-0.665 (-0.995--0.334)	<0.0001	-0.303 (-0.366--0.241)	<0.00001
Triglycerides in Very Large HDL	XL-Tryg	0.139 (0.054-0.223)	0.001	0.096 (0.018-0.174)	0.016	0.206 (-0.079-0.491)	0.154	0.096 (0.021-0.172)	0.012
**Large HDL**									
Conc. of Large HDL Particles	L-HDL	-0.343 (-0.446--0.240)	<0.00001	-0.393 (-0.470--0.315)	<0.00001	-0.757 (-1.141--0.374)	<0.0001	-0.403 (-0.488--0.319)	<0.00001
Total Lipids in Large HDL	L-L	-0.306 (-0.403--0.209)	<0.00001	-0.360 (-0.436--0.284)	<0.00001	-0.768 (-1.181--0.356)	<0.0001	-0.378 (-0.459--0.298)	<0.00001
Phospholipids in Large HDL	L-P	-0.249 (-0.355--0.142)	<0.0001	-0.313 (-0.402--0.223)	<0.00001	-0.748 (-1.199--0.297)	0.001	-0.335 (-0.423--0.247)	<0.00001
Cholesterol in Large HDL	L-C	-0.368 (-0.476--0.260)	<0.00001	-0.408 (-0.487--0.329)	<0.00001	-0.760 (-1.131--0.389)	<0.0001	-0.421 (-0.505--0.337)	<0.00001
Cholesteryl Esters in Large HDL	L-Este-E	-0.387 (-0.494--0.279)	<0.00001	-0.419 (-0.496--0.341)	<0.00001	-0.747 (-1.116--0.379)	<0.0001	-0.431 (-0.514--0.349)	<0.00001
Free Cholesterol in Large HDL	L-Chol-C	-0.285 (-0.405--0.166)	<0.00001	-0.352 (-0.450--0.254)	<0.00001	-0.749 (-1.125--0.373)	<0.0001	-0.369 (-0.466--0.272)	<0.00001
Triglycerides in Large HDL	L-Tryg	0.079 (0.009-0.150)	0.027	0.048 (-0.016-0.112)	0.140	0.107 (-0.241-0.455)	0.543	0.042 (-0.026-0.110)	0.231
**Medium HDL**									
Conc. of Medium HDL Particles	M-HDL	-0.097 (-0.174--0.020)	0.013	-0.170 (-0.243--0.097)	<0.00001	-0.315 (-0.817-0.188)	0.216	-0.168 (-0.245--0.091)	<0.00001
Total Lipids in Medium HDL	M-L	-0.054 (-0.128-0.019)	0.147	-0.128 (-0.200--0.057)	<0.0001	-0.223 (-0.734-0.287)	0.387	-0.124 (-0.200--0.047)	0.002
Phospholipids in Medium HDL	M-P	-0.002 (-0.074-0.069)	0.945	-0.078 (-0.149--0.007)	0.032	-0.102 (-0.606-0.402)	0.688	-0.069 (-0.146-0.007)	0.075
Cholesterol in Medium HDL	M-C	-0.154 (-0.238--0.070)	<0.001	-0.219 (-0.292--0.145)	<0.00001	-0.436 (-0.923-0.052)	0.079	-0.220 (-0.297--0.143)	<0.00001
Cholesteryl Esters in Medium HDL	M-Este-E	-0.163 (-0.248--0.077)	<0.001	-0.223 (-0.297--0.150)	<0.00001	-0.440 (-0.915-0.036)	0.069	-0.225 (-0.301--0.148)	<0.00001
Free Cholesterol in Medium HDL	M-Chol-C	-0.110 (-0.188--0.031)	0.006	-0.190 (-0.264--0.116)	<0.00001	-0.401 (-0.929-0.126)	0.134	-0.192 (-0.272--0.112)	<0.00001
Triglycerides in Medium HDL	M-Tryg	0.242 (0.166-0.319)	<0.00001	0.219 (0.161-0.277)	<0.00001	0.521 (0.175-0.866)	0.004	0.242 (0.182-0.303)	<0.00001
**Small HDL**									
Conc. of Small HDL Particles	S-HDL	0.144 (0.078-0.210)	<0.0001	0.090 (0.024-0.157)	0.008	0.133 (-0.291-0.556)	0.535	0.100 (0.033-0.167)	0.004
Total Lipids in Small HDL	S-L	0.200 (0.132-0.267)	<0.00001	0.138 (0.072-0.204)	<0.0001	0.243 (-0.176-0.661)	0.252	0.155 (0.086-0.223)	<0.00001
Phospholipids in Small HDL	S-P	0.206 (0.138-0.274)	<0.00001	0.140 (0.074-0.206)	<0.0001	0.263 (-0.154-0.680)	0.212	0.157 (0.088-0.226)	<0.00001
Cholesterol in Small HDL	S-C	0.111 (0.044-0.179)	0.001	0.056 (-0.012-0.124)	0.108	0.057 (-0.381-0.495)	0.796	0.063 (-0.006-0.132)	0.072
Cholesteryl Esters in Small HDL	S-Este-E	0.093 (0.026-0.160)	0.007	0.047 (-0.020-0.114)	0.171	0.056 (-0.355-0.467)	0.787	0.056 (-0.011-0.123)	0.100
Free Cholesterol in Small HDL	S-Chol-C	0.155 (0.080-0.231)	<0.00001	0.076 (0.003-0.150)	0.042	0.053 (-0.445-0.552)	0.831	0.075 (-0.001-0.152)	0.054
Triglycerides in Small HDL	S-Tryg	0.308 (0.225-0.390)	<0.00001	0.295 (0.235-0.355)	<0.00001	0.655 (0.341-0.968)	<0.00001	0.329 (0.268-0.390)	<0.00001
**VLDL Ratios, Extra Large (%)**									
Phospholipids:Tot. Lipids	P:L-XXL VLDL	0.069 (0.035-0.103)	<0.0001	0.071 (0.044-0.097)	<0.00001	0.230 (0.014-0.446)	0.037	0.086 (0.060-0.112)	<0.00001
Cholesterol:Tot. Lipids	C:L-XXL VLDL	-0.136 (-0.196--0.075)	<0.0001	-0.150 (-0.199--0.100)	<0.00001	-0.570 (-0.860--0.280)	<0.0001	-0.183 (-0.234--0.132)	<0.00001
Cholesteryl Esters:Tot. Lipids	CE:L-XXL VLDL	-0.090 (-0.147--0.032)	0.002	-0.111 (-0.163--0.058)	<0.0001	-0.423 (-0.737--0.109)	0.009	-0.134 (-0.187--0.081)	<0.00001
Free Cholesterol:Tot. Lipids	CholC:L-XXL VLDL	-0.156 (-0.220--0.092)	<0.00001	-0.164 (-0.215--0.113)	<0.00001	-0.499 (-0.797--0.201)	0.001	-0.195 (-0.246--0.143)	<0.00001
Triglicerydes:Tot. Lipids	Tryg:L-XXL VLDL	0.078 (0.006-0.149)	0.033	0.076 (0.031-0.121)	0.001	0.614 (0.113-1.114)	0.017	0.092 (0.050-0.134)	<0.0001

Table S2 Continued

**Very Large VLDL Ratios (%)**									
Phospholipids:Tot. Lipids	P:L-XL VLDL	0.041 (0.005-0.077)	0.026	0.038 (-0.009-0.086)	0.113	0.158 (0.002-0.314)	0.047	0.044 (0.003-0.085)	0.036
Cholesterol:Tot. Lipids	C:L-XL VLDL	-0.278 (-0.356--0.200)	<0.00001	-0.276 (-0.332--0.219)	<0.00001	-0.646 (-0.944--0.347)	<0.0001	-0.318 (-0.378--0.259)	<0.00001
Cholesteryl Esters:Tot. Lipids	CE:L-XL VLDL	-0.281 (-0.362--0.201)	<0.00001	-0.277 (-0.336--0.218)	<0.00001	-0.607 (-0.893--0.320)	<0.0001	-0.318 (-0.380--0.255)	<0.00001
Free Cholesterol:Tot. Lipids	CholC:L-XL VLDL	-0.241 (-0.308--0.174)	<0.00001	-0.247 (-0.297--0.196)	<0.00001	-0.683 (-1.029--0.337)	<0.0001	-0.286 (-0.339--0.232)	<0.00001
Triglicerydes:Tot. Lipids	Tryg:L-XL VLDL	0.224 (0.159-0.289)	<0.00001	0.227 (0.179-0.276)	<0.00001	0.674 (0.323-1.025)	<0.0001	0.269 (0.217-0.322)	<0.00001
**Large VLDL Ratios (%)**									
Phospholipids:Tot. Lipids	P:L-L VLDL	0.164 (0.109-0.220)	<0.00001	0.192 (0.141-0.243)	<0.00001	0.463 (0.098-0.829)	0.014	0.211 (0.164-0.258)	<0.00001
Cholesterol:Tot. Lipids	C:L-L VLDL	-0.185 (-0.254--0.115)	<0.00001	-0.178 (-0.231--0.124)	<0.00001	-0.645 (-0.992--0.299)	<0.0001	-0.221 (-0.279--0.163)	<0.00001
Cholesteryl Esters:Tot. Lipids	CE:L-L VLDL	-0.280 (-0.360--0.200)	<0.00001	-0.266 (-0.324--0.207)	<0.00001	-0.656 (-0.976--0.336)	<0.0001	-0.312 (-0.375--0.248)	<0.00001
Free Cholesterol:Tot. Lipids	CholC:L-L VLDL	0.073 (0.025-0.121)	0.003	0.087 (0.043-0.131)	<0.0001	0.108 (-0.307-0.523)	0.606	0.081 (0.039-0.124)	<0.0001
Triglicerydes:Tot. Lipids	Tryg:L-L VLDL	0.035 (-0.016-0.085)	0.176	0.036 (-0.011-0.083)	0.136	0.337 (-0.042-0.715)	0.081	0.063 (0.014-0.111)	0.012
**Medium VLDL Ratios (%)**									
Phospholipids:Tot. Lipids	P:L-M VLDL	-0.173 (-0.256--0.090)	<0.0001	-0.193 (-0.262--0.124)	<0.00001	-0.429 (-0.748--0.110)	0.009	-0.215 (-0.282--0.148)	<0.00001
Cholesterol:Tot. Lipids	C:L-M VLDL	-0.306 (-0.406--0.206)	<0.00001	-0.292 (-0.364--0.221)	<0.00001	-0.440 (-0.733--0.147)	0.004	-0.306 (-0.380--0.233)	<0.00001
Cholesteryl Esters:Tot. Lipids	CE:L-M VLDL	-0.350 (-0.466--0.234)	<0.00001	-0.325 (-0.406--0.244)	<0.00001	-0.248 (-0.536-0.040)	0.091	-0.293 (-0.391--0.194)	<0.00001
Free Cholesterol:Tot. Lipids	CholC:L-M VLDL	-0.246 (-0.337--0.154)	<0.00001	-0.241 (-0.308--0.173)	<0.00001	-0.486 (-0.791--0.182)	0.002	-0.265 (-0.334--0.196)	<0.00001
Triglicerydes:Tot. Lipids	Tryg:L-M VLDL	0.256 (0.176-0.336)	<0.00001	0.257 (0.198-0.316)	<0.00001	0.636 (0.319-0.952)	<0.0001	0.294 (0.232-0.357)	<0.00001
**Small VLDL Ratios (%)**									
Phospholipids:Tot. Lipids	P:L-S VLDL	-0.232 (-0.320--0.144)	<0.00001	-0.245 (-0.309--0.180)	<0.00001	-0.494 (-0.778--0.210)	0.001	-0.274 (-0.340--0.208)	<0.00001
Cholesterol:Tot. Lipids	C:L-S VLDL	-0.162 (-0.240--0.083)	<0.0001	-0.156 (-0.215--0.096)	<0.00001	-0.425 (-0.722--0.129)	0.005	-0.194 (-0.257--0.132)	<0.00001
Cholesteryl Esters:Tot. Lipids	CE:L-S VLDL	-0.084 (-0.152--0.015)	0.017	-0.070 (-0.125--0.016)	0.012	-0.378 (-0.706--0.050)	0.025	-0.112 (-0.170--0.055)	<0.0001
Free Cholesterol:Tot. Lipids	CholC:L-S VLDL	-0.247 (-0.339--0.155)	<0.00001	-0.251 (-0.317--0.185)	<0.00001	-0.462 (-0.741--0.182)	0.002	-0.277 (-0.345--0.209)	<0.00001
Triglicerydes:Tot. Lipids	Tryg:L-S VLDL	0.174 (0.100-0.248)	<0.00001	0.177 (0.123-0.231)	<0.00001	0.552 (0.240-0.865)	0.001	0.218 (0.160-0.277)	<0.00001
**Extra Small VLDL Ratios (%)**									
Phospholipids:Tot. Lipids	P:L-XS VLDL	0.124 (0.035-0.213)	0.007	0.140 (0.069-0.211)	<0.0001	0.523 (0.142-0.903)	0.008	0.168 (0.099-0.237)	<0.00001
Cholesterol:Tot. Lipids	C:L-XS VLDL	-0.297 (-0.402--0.193)	<0.00001	-0.290 (-0.365--0.216)	<0.00001	-0.412 (-0.687--0.137)	0.004	-0.296 (-0.369--0.223)	<0.00001
Cholesteryl Esters:Tot. Lipids	CE:L-XS VLDL	-0.307 (-0.412--0.202)	<0.00001	-0.307 (-0.383--0.231)	<0.00001	-0.406 (-0.684--0.128)	0.005	-0.304 (-0.377--0.230)	<0.00001
Free Cholesterol:Tot. Lipids	CholC:L-XS VLDL	-0.162 (-0.265--0.059)	0.002	-0.125 (-0.195--0.056)	<0.0001	-0.306 (-0.579--0.033)	0.028	-0.172 (-0.246--0.098)	<0.0001
Triglicerydes:Tot. Lipids	Tryg:L-XS VLDL	0.264 (0.174-0.353)	<0.00001	0.263 (0.200-0.326)	<0.00001	0.521 (0.232-0.809)	0.001	0.291 (0.226-0.355)	<0.00001

Table S2 Continued

**ILDL Ratios (%)**									
Phospholipids:Tot. Lipids	P:L-IDL	-0.161 (-0.235--0.088)	<0.0001	-0.078 (-0.139--0.017)	0.012	0.055 (-0.333-0.443)	0.778	-0.048 (-0.111-0.016)	0.141
Cholesterol:Tot. Lipids	C:L-IDL	-0.154 (-0.263--0.045)	0.006	-0.188 (-0.267--0.109)	<0.00001	-0.316 (-0.597--0.036)	0.028	-0.212 (-0.289--0.135)	<0.00001
Cholesteryl Esters:Tot. Lipids	CE:L-IDL	-0.098 (-0.196-0.001)	0.051	-0.166 (-0.242--0.091)	<0.0001	-0.293 (-0.617-0.030)	0.075	-0.192 (-0.269--0.115)	<0.00001
Free Cholesterol:Tot. Lipids	CholC:L-IDL	-0.145 (-0.237--0.053)	0.002	-0.101 (-0.167--0.036)	0.002	-0.330 (-0.617--0.042)	0.025	-0.137 (-0.206--0.068)	<0.0001
Triglicerydes:Tot. Lipids	Tryg:L-IDL	0.247 (0.149-0.344)	<0.00001	0.259 (0.189-0.330)	<0.00001	0.460 (0.171-0.749)	0.002	0.282 (0.212-0.351)	<0.00001
**LDL Ratios Large (%)**									
Phospholipids:Tot. Lipids	P:L-L LDL	0.044 (-0.033-0.121)	0.262	0.056 (-0.012-0.124)	0.108	0.117 (-0.229-0.464)	0.503	0.038 (-0.033-0.110)	0.292
Cholesterol:Tot. Lipids	C:L-L LDL	-0.281 (-0.377--0.186)	<0.00001	-0.287 (-0.357--0.218)	<0.00001	-0.546 (-0.858--0.234)	0.001	-0.307 (-0.379--0.235)	<0.00001
Cholesteryl Esters:Tot. Lipids	CE:L-L LDL	-0.085 (-0.156--0.013)	0.020	-0.098 (-0.158--0.038)	0.001	-0.182 (-0.590-0.225)	0.376	-0.085 (-0.147--0.022)	0.008
Free Cholesterol:Tot. Lipids	CholC:L-L LDL	-0.304 (-0.410--0.198)	<0.00001	-0.283 (-0.355--0.211)	<0.00001	-0.455 (-0.737--0.173)	0.002	-0.310 (-0.384--0.236)	<0.00001
Triglicerydes:Tot. Lipids	Tryg:L-L LDL	0.255 (0.158-0.352)	<0.00001	0.256 (0.186-0.326)	<0.00001	0.495 (0.192-0.799)	0.002	0.281 (0.212-0.350)	<0.00001
**Medium LDL Ratios (%)**									
Phospholipids:Tot. Lipids	P:L-M LDL	-0.076 (-0.151--0.001)	0.046	-0.084 (-0.146--0.021)	0.009	-0.324 (-0.741-0.093)	0.126	-0.090 (-0.150--0.030)	0.003
Cholesterol:Tot. Lipids	C:L-M LDL	-0.155 (-0.263--0.046)	0.005	-0.144 (-0.222--0.066)	<0.0001	-0.308 (-0.601--0.014)	0.040	-0.166 (-0.240--0.093)	<0.0001
Cholesteryl Esters:Tot. Lipids	CE:L-M LDL	0.172 (0.102-0.243)	<0.00001	0.168 (0.108-0.228)	<0.00001	0.509 (0.151-0.867)	0.006	0.176 (0.120-0.231)	<0.00001
Free Cholesterol:Tot. Lipids	CholC:L-M LDL	-0.315 (-0.405--0.225)	<0.00001	-0.280 (-0.346--0.214)	<0.00001	-0.560 (-0.869--0.251)	0.001	-0.319 (-0.390--0.248)	<0.00001
Triglicerydes:Tot. Lipids	Tryg:L-M LDL	0.202 (0.108-0.296)	<0.0001	0.200 (0.131-0.268)	<0.00001	0.428 (0.134-0.721)	0.005	0.231 (0.163-0.300)	<0.00001
**Small LDL Ratios (%)**									
Phospholipids:Tot. Lipids	P:L-S LDL	-0.172 (-0.233--0.112)	<0.00001	-0.150 (-0.209--0.09)	<0.00001	-0.465 (-0.829--0.101)	0.013	-0.161 (-0.217--0.106)	<0.00001
Cholesterol:Tot. Lipids	C:L-S LDL	-0.026 (-0.111-0.059)	0.547	-0.057 (-0.129-0.016)	0.123	-0.282 (-0.598-0.033)	0.078	-0.099 (-0.166--0.032)	0.004
Cholesteryl Esters:Tot. Lipids	CE:L-S LDL	0.180 (0.117-0.243)	<0.00001	0.150 (0.091-0.209)	<0.00001	0.452 (0.087-0.816)	0.016	0.148 (0.094-0.203)	<0.00001
Free Cholesterol:Tot. Lipids	CholC:L-S LDL	-0.266 (-0.357--0.175)	<0.00001	-0.228 (-0.293--0.164)	<0.00001	-0.508 (-0.809--0.206)	0.001	-0.264 (-0.336--0.191)	<0.00001
Triglicerydes:Tot. Lipids	Tryg:L-S LDL	0.252 (0.165-0.338)	<0.00001	0.239 (0.177-0.302)	<0.00001	0.513 (0.222-0.804)	0.001	0.280 (0.213-0.347)	<0.00001
**HDL Ratios, Very Large (%)**									
Phospholipids:Tot. Lipids	P:L-XL HDL	-0.239 (-0.342--0.137)	<0.0001	-0.260 (-0.340--0.180)	<0.00001	-0.412 (-0.641--0.184)	0.001	-0.272 (-0.345--0.199)	<0.00001
Cholesterol:Tot. Lipids	C:L-XL HDL	0.095 (0.007-0.182)	0.035	0.124 (0.049-0.198)	0.001	0.131 (-0.141-0.403)	0.340	0.126 (0.054-0.198)	0.001
Cholesteryl Esters:Tot. Lipids	CE:L-XL HDL	-0.172 (-0.276--0.067)	0.001	-0.180 (-0.259--0.101)	<0.0001	-0.331 (-0.645--0.016)	0.040	-0.188 (-0.273--0.104)	<0.0001
Free Cholesterol:Tot. Lipids	CholC:L-XL HDL	0.346 (0.254-0.437)	<0.00001	0.386 (0.313-0.458)	<0.00001	0.665 (0.330-1.000)	0.000	0.388 (0.312-0.465)	<0.00001
Triglicerydes:Tot. Lipids	Tryg:L-XL HDL	0.347 (0.256-0.437)	<0.00001	0.334 (0.267-0.401)	<0.00001	0.628 (0.335-0.922)	0.000	0.357 (0.288-0.426)	<0.00001

Table S2 Continued

**Large HDL Ratios (%)**									
Phospholipids:Tot. Lipids	P:L-L HDL	0.271 (0.025-0.517)	0.031	0.315 (0.107-0.522)	0.003	0.777 (0.412-1.143)	<0.0001	0.362 (0.171-0.552)	<0.001
Cholesterol:Tot. Lipids	C:L-L HDL	-0.500 (-0.627--0.374)	<0.00001	-0.448 (-0.534--0.363)	<0.00001	-0.55 (-0.858--0.242)	0.001	-0.429 (-0.524--0.334)	<0.00001
Cholesteryl Esters:Tot. Lipids	CE:L-L HDL	-0.489 (-0.614--0.364)	<0.00001	-0.429 (-0.514--0.344)	<0.00001	-0.493 (-0.813--0.174)	0.003	-0.422 (-0.515--0.328)	<0.00001
Free Cholesterol:Tot. Lipids	CholC:L-L HDL	-0.092 (-0.219-0.036)	0.159	-0.174 (-0.332--0.016)	0.031	-0.517 (-0.779--0.254)	<0.0001	-0.219 (-0.360--0.078)	0.002
Triglicerydes:Tot. Lipids	Tryg:L-L HDL	0.301 (0.210-0.393)	<0.00001	0.319 (0.250-0.389)	<0.00001	0.699 (0.342-1.057)	<0.0001	0.336 (0.264-0.408)	<0.00001
**Medium HDL Ratios (%)**									
Phospholipids:Tot. Lipids	P:L-M HDL	0.368 (0.265-0.470)	<0.00001	0.350 (0.279-0.421)	<0.00001	0.765 (0.412-1.118)	<0.0001	0.383 (0.310-0.456)	<0.00001
Cholesterol:Tot. Lipids	C:L-M HDL	-0.349 (-0.448--0.249)	<0.00001	-0.342 (-0.414--0.269)	<0.00001	-0.696 (-1.045--0.347)	<0.0001	-0.367 (-0.440--0.293)	<0.00001
Cholesteryl Esters:Tot. Lipids	CE:L-M HDL	-0.333 (-0.432--0.234)	<0.00001	-0.318 (-0.391--0.245)	<0.00001	-0.611 (-0.935--0.287)	<0.0001	-0.338 (-0.411--0.266)	<0.00001
Free Cholesterol:Tot. Lipids	CholC:L-M HDL	-0.237 (-0.328--0.146)	<0.00001	-0.302 (-0.373--0.230)	<0.00001	-0.711 (-1.177--0.245)	0.003	-0.312 (-0.392--0.231)	<0.00001
Triglicerydes:Tot. Lipids	Tryg:L-M HDL	0.264 (0.183-0.344)	<0.00001	0.278 (0.216-0.340)	<0.00001	0.701 (0.362-1.039)	<0.0001	0.302 (0.240-0.364)	<0.00001
**Small HDL Ratios (%)**									
Phospholipids:Tot. Lipids	P:L-S HDL	0.030 (-0.053-0.113)	0.473	0.011 (-0.055-0.076)	0.747	0.240 (-0.128-0.608)	0.198	0.022 (-0.042-0.085)	0.505
Cholesterol:Tot. Lipids	C:L-S HDL	-0.222 (-0.312--0.131)	<0.00001	-0.209 (-0.275--0.143)	<0.00001	-0.541 (-0.856--0.226)	0.001	-0.239 (-0.307--0.172)	<0.00001
Cholesteryl Esters:Tot. Lipids	CE:L-S HDL	-0.178 (-0.269--0.087)	<0.0001	-0.167 (-0.236--0.097)	<0.00001	-0.412 (-0.720--0.104)	0.009	-0.187 (-0.256--0.117)	<0.00001
Free Cholesterol:Tot. Lipids	CholC:L-S HDL	-0.141 (-0.214--0.067)	<0.001	-0.168 (-0.232--0.104)	<0.00001	-0.758 (-1.260--0.256)	0.004	-0.208 (-0.275--0.141)	<0.00001
Triglicerydes:Tot. Lipids	Tryg:L-S HDL	0.269 (0.185-0.353)	<0.00001	0.285 (0.222-0.349)	<0.00001	0.680 (0.359-1.00)	<0.0001	0.316 (0.253-0.379)	<0.00001

Table S3: Absolute differences in the associations of log-levels of NMR metabolites with log- levels of liver fat (per 1 SD) , by different characteristics

		Sex		Age		Smoking		BMI		Diabetes		CVDs		Chronic Disease	
**Metabolites**		**Men vs Women**		**<55 vs 55+ yrs.**		**No vs Yes**		**<25 vs 25+ kg/m^2**		**No vs Yes**		**No vs Yes**		**No vs Yes**	
**Cholesterol (mmol/L)**	**Label**	**Δ β-coef.**	**Δ SE**	**Δ β-coef.**	**Δ SE**	**Δ β-coef.**	**Δ SE**	**Δ β-coef.**	**Δ SE**	**Δ β-coef.**	**Δ SE**	**Δ β-coef.**	**Δ SE**	**Δ β-coef.**	**Δ SE**
Total Cholesterol	TC-C	-0.03	0.00	0.12	0.01	0.02	-0.02	0.13	0.02	0.20	-0.16	0.18	-0.03	0.19	-0.01
Non HDL-C	nHDL-C	-0.08	0.00	0.14	0.02	0.04	-0.02	0.10	0.03	0.16	-0.15	0.16	-0.03	0.18	-0.01
Remnant Cholesterol	RC-C	-0.09	-0.01	0.14	0.02	0.06	-0.01	0.09	0.03	0.13	-0.14	0.17	-0.03	0.20	-0.01
VLDL Cholesterol	VLDL-C	-0.10	-0.01	0.12	0.02	0.05	-0.01	0.02	0.03	-0.06	-0.11	0.07	-0.04	0.10	-0.01
Clinical LDL Cholesterol	cLDL-C	-0.08	0.00	0.14	0.02	0.03	-0.02	0.13	0.02	0.20	-0.15	0.16	-0.03	0.18	-0.01
LDL Cholesterol	LDL-C	-0.07	0.00	0.14	0.02	0.01	-0.02	0.11	0.02	0.18	-0.16	0.15	-0.04	0.16	-0.01
HDL Cholesterol	HDL-C	0.06	0.01	-0.09	0.00	-0.04	-0.02	0.15	-0.01	0.36	-0.19	0.22	-0.04	0.12	-0.02
**Triglycerides (mmol/L)**															
Total Triglycerides	Try-T	-0.07	0.00	0.04	0.01	0.00	-0.01	-0.10	0.02	-0.28	-0.12	-0.08	-0.04	0.00	-0.01
Triglycerides in VLDL	VLDL-T	-0.06	0.00	0.04	0.01	-0.01	-0.01	-0.10	0.02	-0.31	-0.12	-0.11	-0.03	-0.02	-0.01
Triglycerides in LDL	LDL-T	-0.08	-0.01	0.06	0.02	-0.01	-0.01	-0.12	0.04	-0.15	-0.12	0.04	-0.05	0.07	-0.01
Triglycerides in HDL	HDL-T	-0.02	-0.01	-0.03	0.00	0.02	-0.02	-0.14	0.00	-0.26	-0.14	-0.03	-0.03	0.01	-0.01
**Phospholipids (mmol/L)**															
Total Phospholipids in Lipoproteins	Phos-P	-0.02	-0.01	0.09	0.00	0.02	-0.03	0.06	0.01	0.10	-0.18	0.18	-0.04	0.21	-0.01
Phospholipids in VLDL	VLDL-P	-0.09	-0.01	0.09	0.02	0.03	-0.01	-0.05	0.02	-0.19	-0.11	0.00	-0.04	0.05	-0.01
Phospholipids in LDL	LDL-P	-0.09	0.00	0.15	0.02	0.02	-0.02	0.11	0.02	0.17	-0.16	0.15	-0.04	0.17	-0.01
Phospholipids in HDL	HDL-P	0.08	0.01	-0.05	0.00	-0.02	-0.02	0.04	-0.02	0.18	-0.22	0.19	-0.05	0.15	-0.03
**Cholesteryl esters (mmol/L)**															
Total Esterified Cholesterol	Es-CE	-0.03	0.00	0.12	0.01	0.02	-0.02	0.13	0.01	0.21	-0.16	0.18	-0.03	0.19	-0.01
Cholesteryl Esters in VLDL	VLDL-CE	-0.10	0.00	0.13	0.03	0.06	-0.01	0.05	0.03	0.03	-0.11	0.11	-0.03	0.13	-0.01
Cholesteryl Esters in LDL	LDL-CE	-0.08	0.00	0.13	0.02	0.01	-0.02	0.10	0.02	0.15	-0.16	0.14	-0.04	0.16	-0.01
Cholesteryl Esters in HDL	HDL-CE	0.05	0.01	-0.10	0.00	-0.05	-0.02	0.15	-0.01	0.36	-0.18	0.22	-0.04	0.11	-0.01
**Free cholesterol (mmol/L)**															
Total Free Cholesterol	Chol-FC	-0.05	-0.01	0.13	0.02	0.03	-0.02	0.11	0.02	0.18	-0.15	0.17	-0.03	0.19	-0.01
Free Cholesterol in VLDL	VLDL-FC	-0.10	-0.01	0.11	0.02	0.04	-0.01	-0.02	0.03	-0.15	-0.11	0.02	-0.04	0.06	-0.01
Free Cholesterol in LDL	LDL-FC	-0.06	0.01	0.13	0.02	0.02	-0.02	0.15	0.02	0.25	-0.16	0.16	-0.03	0.16	-0.01
Free Cholesterol in HDL	HDL-FC	0.09	0.01	-0.05	0.00	-0.01	-0.03	0.10	-0.01	0.34	-0.21	0.20	-0.04	0.15	-0.02
**Total lipids (mmol/L)**															
Total Lipids in Lipoprotein Particles	Lp-L	-0.06	-0.01	0.11	0.01	0.02	-0.02	0.05	0.02	0.06	-0.15	0.15	-0.04	0.19	-0.01
Total Lipids in VLDL	VLDL-L	-0.08	-0.01	0.07	0.02	0.02	-0.01	-0.06	0.02	-0.22	-0.11	-0.04	-0.04	0.02	-0.01
Total Lipids in LDL	LDL-L	-0.08	0.00	0.14	0.02	0.01	-0.02	0.10	0.02	0.16	-0.16	0.15	-0.04	0.16	-0.01
Total Lipids in HDL	HDL-L	0.07	0.01	-0.06	0.00	-0.03	-0.02	0.08	-0.02	0.25	-0.21	0.20	-0.04	0.14	-0.02

Table S3 Continued

**Lipoprotein Conc. (mmol/L)**															
Total Conc. of Lipoprotein Particles	c-LPp	-0.02	0.01	0.03	0.00	-0.03	-0.02	0.11	-0.02	0.15	-0.20	0.18	-0.05	0.15	-0.02
Conc. of VLDL Particles	cVLDL-LPp	-0.10	-0.01	0.11	0.02	0.05	-0.01	-0.01	0.03	-0.12	-0.11	0.05	-0.04	0.07	-0.01
Conc. of LDL Particles	cLDL-LPp	-0.08	0.00	0.15	0.02	0.03	-0.01	0.11	0.03	0.15	-0.14	0.12	-0.04	0.14	-0.01
Conc. of HDL Particles	cHDL-LPp	-0.01	0.01	0.00	0.00	-0.05	-0.02	0.10	-0.02	0.14	-0.21	0.17	-0.04	0.14	-0.02
Average Diameter for VLDL Particles	dVLDL-LPp	-0.03	0.01	0.04	0.01	-0.05	-0.01	-0.12	0.00	-0.39	-0.13	-0.15	-0.03	-0.06	-0.01
Average Diameter for LDL Particles	dLDL-LPp	0.07	0.00	0.00	0.01	0.01	-0.01	0.22	-0.01	0.42	-0.14	0.14	-0.02	0.17	-0.01
Average Diameter for HDL Particles	dHDL-LPp	0.03	0.01	-0.14	0.02	0.01	-0.01	0.12	0.01	0.44	-0.16	0.18	-0.03	0.13	-0.01
**Other lipids (mmol/L)**															
Phosphoglycerides	Pglyc-OL	-0.02	-0.01	0.07	0.00	0.01	-0.03	0.04	0.00	0.05	-0.20	0.17	-0.05	0.20	-0.02
Phosphoglycerides:Tryglicerides	Pglyc:Tryg	-0.03	0.00	0.05	0.01	0.00	-0.02	-0.15	0.01	-0.40	-0.14	-0.18	-0.03	-0.09	-0.01
Total Cholines	Chol-OL	-0.01	0.00	0.08	0.00	0.00	-0.03	0.08	0.00	0.10	-0.20	0.19	-0.04	0.19	-0.02
Phosphatidylcholines	Pdchol-OL	0.00	0.00	0.07	0.00	0.01	-0.03	0.06	0.00	0.07	-0.21	0.16	-0.05	0.18	-0.02
Sphingomyelins	Spyng-OL	-0.03	0.01	0.13	0.01	0.01	-0.02	0.13	0.00	0.31	-0.18	0.22	-0.04	0.20	-0.02
**Apolipoproteins (gl/L)**															
Apolipoprotein B	Apo-B	-0.08	0.00	0.15	0.02	0.04	-0.01	0.10	0.03	0.13	-0.14	0.14	-0.03	0.16	-0.01
Apolipoprotein A1	Apo-A1	0.04	0.01	-0.05	0.00	-0.04	-0.02	0.07	-0.02	0.18	-0.21	0.19	-0.04	0.14	-0.02
ApoB:ApoA Ratio	ApoB:ApoA	-0.08	0.01	0.16	0.03	0.06	-0.01	0.06	0.02	0.01	-0.14	0.02	-0.03	0.04	-0.01
**Fatty acids (mmol/L)**															
Total Fatty Acids	TFA-FA	-0.10	-0.01	0.06	0.01	-0.01	-0.01	-0.03	0.02	-0.12	-0.14	0.08	-0.04	0.13	-0.01
Degree of Unsaturation	UnsatD-FA	-0.03	0.00	0.09	0.02	-0.02	-0.02	0.26	-0.01	0.27	-0.12	0.09	-0.01	0.08	0.00
Omega-3 FA	O3-FA	-0.03	-0.02	0.20	0.02	0.00	-0.01	0.07	0.00	0.17	-0.16	0.07	-0.05	0.14	-0.01
Omega-6 FA	O6-FA	-0.05	-0.01	0.10	0.01	-0.03	-0.02	0.13	0.01	-0.01	-0.16	0.15	-0.03	0.18	-0.01
Polyunsaturated FA	PolyU-FA	-0.06	-0.02	0.15	0.01	-0.02	-0.02	0.14	0.02	0.02	-0.17	0.15	-0.04	0.20	0.00
Monounsaturated FA	MomoS-FA	-0.10	0.00	0.02	0.01	-0.02	-0.01	-0.09	0.02	-0.23	-0.13	0.01	-0.03	0.07	-0.01
Saturated FA	Sat-FA	-0.11	-0.01	0.03	0.01	0.02	-0.01	-0.07	0.02	-0.07	-0.13	0.09	-0.04	0.13	-0.01
Linoleic FA	LA-FA	-0.01	-0.01	0.07	0.02	-0.04	-0.01	0.13	0.02	-0.05	-0.14	0.11	-0.03	0.14	0.00
Docosahexaenoic FA	DA-FA	-0.04	-0.02	0.24	0.03	-0.02	-0.01	0.17	0.01	0.33	-0.12	0.09	-0.03	0.15	0.00
Omega-3: Total FA %	O3:Tot-FA%	0.00	-0.02	0.20	0.02	0.01	-0.01	0.13	-0.01	0.30	-0.10	0.04	-0.03	0.11	-0.01
Omega-6: Total FA %	O6:Tot-FA%	0.13	0.00	-0.08	0.01	-0.09	-0.01	0.13	0.03	0.10	-0.11	0.04	-0.02	-0.05	0.00
Polyunsaturated:Total FA %	PU:Tot-FA%	0.10	0.01	-0.01	0.01	-0.09	-0.02	0.15	0.01	0.12	-0.11	0.02	-0.01	-0.06	0.00
Monounsaturated: Total FA %	MS:Tot-FA%	-0.05	0.00	0.01	0.01	0.00	-0.01	-0.20	0.00	-0.39	-0.13	-0.09	-0.02	-0.03	-0.01
Saturated : Total FA%	Sat: Tot-FA%	-0.06	0.00	-0.01	0.01	0.13	-0.01	-0.20	-0.01	-0.06	-0.13	-0.01	-0.02	0.05	-0.01
Linoleic: Total FA%	LA: Tot-FA%	0.10	0.00	-0.13	0.02	-0.11	-0.01	0.17	0.01	0.07	-0.12	0.02	-0.01	-0.04	0.00

Table S3 Continued

Docosahexaenoic : Total FA%	DA: Tot-FA%	0.00	-0.01	0.15	0.03	-0.02	-0.01	0.18	0.00	0.32	-0.11	0.01	-0.01	0.07	0.00
Polyunsaturated: Monounsaturated FA %	PU: MS-FA%	0.07	0.00	-0.01	0.01	-0.04	-0.01	0.18	0.00	0.25	-0.12	0.05	-0.01	-0.01	0.00
Omega-6:Omega-3 FA %	O6: O3-FA%	0.01	-0.02	-0.19	0.02	-0.01	-0.01	-0.02	-0.01	-0.18	-0.14	-0.01	-0.03	-0.08	-0.01
**Amino acids (mmol/L)**															
Alanine	Ala-AA	0.05	0.01	-0.03	0.00	-0.09	-0.02	-0.17	-0.02	-0.32	-0.19	-0.09	-0.04	-0.08	-0.03
Glutamine	Glut-AA	0.04	-0.01	-0.04	0.01	0.01	0.00	-0.05	0.00	0.23	-0.09	0.00	-0.02	0.05	-0.01
Glycine	Glyc-AA	0.01	-0.03	0.08	-0.02	-0.03	0.00	0.00	-0.01	-0.06	-0.07	0.07	-0.04	0.13	-0.04
Histidine	Hist-AA	0.02	0.00	-0.01	0.00	-0.06	-0.01	-0.07	-0.01	-0.21	-0.12	-0.12	-0.03	-0.04	-0.01
Tot. Conc. Branched-Chain AA	C-BC-AA	-0.03	0.01	-0.01	0.01	0.08	0.00	-0.22	-0.01	-0.42	-0.12	-0.03	-0.03	-0.02	-0.02
Isoleucine	Isol-AA	0.01	0.00	0.00	0.00	0.07	-0.01	-0.20	-0.02	-0.39	-0.14	0.04	-0.03	-0.01	-0.02
Leucine	Leuc-AA	-0.02	0.01	-0.01	0.01	0.06	-0.01	-0.23	-0.01	-0.39	-0.13	-0.02	-0.03	-0.03	-0.02
Valine	Val-AA	-0.05	0.01	-0.02	0.01	0.08	0.00	-0.21	-0.01	-0.45	-0.11	-0.07	-0.03	-0.02	-0.02
Phenylalanine	Phenyl-AA	-0.03	0.00	-0.04	0.01	0.09	-0.01	-0.18	-0.02	-0.19	-0.11	0.00	-0.02	-0.02	-0.03
Tyrosine	Tyr-AA	0.13	0.00	-0.03	0.00	-0.01	-0.01	-0.23	-0.02	-0.41	-0.10	-0.09	-0.03	-0.02	-0.01
**Glycolysis (mmol/L)**															
Glucose	Gluc-Glyc	0.05	0.00	0.02	0.01	0.05	-0.01	-0.02	-0.02	-0.05	-0.10	0.00	-0.04	0.01	-0.02
Lactate	Lact-Glyc	-0.12	0.01	0.07	0.00	0.01	-0.01	-0.13	-0.01	-0.23	-0.18	-0.03	-0.04	0.06	-0.01
Pyruvate	Pyrv-Glyc	-0.11	0.01	0.03	0.02	-0.02	0.00	-0.11	0.01	-0.26	-0.13	0.07	-0.03	0.11	-0.01
Citrate	Citr-Glyc	0.01	0.00	0.01	0.00	-0.08	-0.01	-0.13	-0.02	-0.01	-0.11	0.07	-0.03	0.01	-0.01
**Ketone bodies (mmol/L)**															
3-Hydroxybutyrate	3Hydx-KB	-0.07	0.03	0.08	-0.01	-0.03	-0.01	-0.02	-0.02	0.09	-0.08	0.07	-0.04	0.02	-0.02
Acetate	Act-KB	-0.06	0.02	-0.05	-0.04	-0.08	-0.03	0.01	-0.04	0.71	-0.27	0.02	-0.06	0.00	-0.04
Acetoacetate	AcetA-KB	-0.08	0.03	0.01	-0.02	-0.02	0.00	0.02	-0.02	-0.03	-0.06	0.02	-0.02	0.01	-0.01
Acetone	Acet-KB	-0.09	0.01	0.03	0.01	-0.06	-0.02	0.07	-0.03	0.21	-0.15	0.13	-0.05	0.07	-0.02
**Fluid balance (mmol/L)**															
Creatinine	Creat-FB	-0.09	0.00	0.04	0.01	-0.10	-0.01	-0.11	0.00	-0.39	-0.16	-0.10	-0.04	-0.05	-0.02
Albumin	Alb-FB	-0.02	0.00	0.04	0.01	-0.04	-0.01	0.02	-0.02	0.09	-0.14	0.04	-0.03	0.02	-0.02
**Inflammation (mmol/L)**															
Glycoprotein Acetyls	GlycA-LP	-0.12	0.00	0.07	0.01	-0.06	0.00	-0.08	0.00	-0.22	-0.12	0.00	-0.04	0.08	-0.01

Table S3 Continued

**Lipoproteins Conc.**															
**VLDL particles, Extremely Large**															
Total Conc. in XXL VLDL	XXL-VLDL	0.02	0.00	-0.02	0.01	-0.01	-0.01	-0.19	-0.01	-0.36	-0.12	-0.16	-0.03	-0.09	-0.02
Total Lipids in XXL VLDL	XXL-L	-0.02	0.01	-0.01	0.01	0.00	-0.01	-0.16	0.00	-0.37	-0.12	-0.15	-0.02	-0.06	-0.01
Phospholipids in XXL VLDL	XXL-PL	0.07	0.01	-0.01	0.01	0.01	-0.01	-0.22	-0.02	-0.33	-0.13	-0.19	-0.03	-0.10	-0.02
Cholesterol in XXL VLDL	XXL-C	-0.02	0.00	0.00	0.01	-0.01	-0.01	-0.16	0.00	-0.34	-0.12	-0.14	-0.03	-0.07	-0.01
Cholesteryl Esters in XXL VLDL	XXL-Este-E	-0.03	0.00	0.00	0.02	-0.01	-0.01	-0.15	0.00	-0.28	-0.12	-0.12	-0.03	-0.05	-0.02
Free Cholesterol in XXL VLDL	XXL-Chol-C	-0.02	0.00	0.00	0.01	0.00	-0.01	-0.17	0.00	-0.38	-0.12	-0.16	-0.03	-0.08	-0.01
Triglycerides in XXL VLDL	XXL-Try	0.00	0.01	-0.04	0.01	-0.06	-0.01	-0.15	0.00	-0.44	-0.13	-0.10	-0.02	-0.02	0.00
**Very Large VLDL**															
Total Conc. in XL VLDL	XL-VLDL	-0.02	0.00	0.04	0.01	-0.01	-0.01	-0.15	0.00	-0.32	-0.12	-0.13	-0.03	-0.06	-0.01
Total Lipids in XL VLDL	XL-L	-0.02	0.00	0.04	0.01	0.00	-0.01	-0.14	0.01	-0.32	-0.11	-0.13	-0.03	-0.05	-0.01
Phospholipids in XL VLDL	XL-Phos	-0.01	0.00	0.03	0.01	0.01	-0.01	-0.15	0.00	-0.29	-0.11	-0.13	-0.03	-0.06	-0.01
Cholesterol in XL VLDL	XL-C	-0.07	0.00	0.07	0.02	0.02	-0.01	-0.09	0.01	-0.27	-0.11	-0.07	-0.03	-0.01	-0.01
Cholesteryl Esters in XL VLDL	XL-Este-E	-0.09	0.00	0.09	0.02	0.02	0.00	-0.05	0.01	-0.23	-0.11	-0.04	-0.04	0.01	-0.01
Free Cholesterol in XL VLDL	XL-Chol-C	-0.04	0.00	0.05	0.01	0.01	-0.01	-0.12	0.01	-0.30	-0.11	-0.11	-0.03	-0.04	-0.01
Triglycerides in XL VLDL	XL-Try	0.00	0.01	0.02	0.01	-0.02	-0.01	-0.16	0.00	-0.33	-0.12	-0.15	-0.03	-0.06	-0.01
**Large VLDL**															
Total Conc. in L VLDL	L-VLDL	-0.06	0.00	0.06	0.01	0.00	-0.01	-0.10	0.01	-0.31	-0.12	-0.11	-0.04	-0.03	-0.01
Total Lipids in L VLDL	L-L	-0.05	0.00	0.06	0.01	-0.01	-0.01	-0.09	0.01	-0.31	-0.12	-0.11	-0.03	-0.03	-0.01
Phospholipids in L VLDL	L-P	-0.03	0.00	0.06	0.02	0.00	-0.01	-0.13	0.00	-0.33	-0.12	-0.12	-0.03	-0.05	-0.01
Cholesterol in L VLDL	L-C	-0.07	0.00	0.09	0.02	0.02	-0.01	-0.07	0.01	-0.28	-0.12	-0.07	-0.04	-0.01	-0.01
Cholesteryl Esters in L VLDL	L-Este-E	-0.09	0.00	0.10	0.02	0.04	-0.01	-0.02	0.01	-0.22	-0.11	-0.02	-0.04	0.02	-0.01
Free Cholesterol in L VLDL	L-Chol-C	-0.06	0.00	0.07	0.02	0.00	-0.01	-0.11	0.01	-0.32	-0.12	-0.11	-0.03	-0.04	-0.01
Triglycerides in L VLDL	L-Tryg	-0.05	0.00	0.05	0.01	-0.03	-0.01	-0.08	0.01	-0.32	-0.12	-0.12	-0.03	-0.03	-0.01
**Medium VLDL**															
Total Conc. in M VLDL	M-VLDL	-0.11	0.00	0.13	0.02	0.03	-0.01	0.05	0.03	-0.05	-0.11	0.07	-0.04	0.10	-0.01
Total Lipids in M VLDL	M-L	-0.10	0.00	0.11	0.02	0.02	-0.01	0.02	0.03	-0.11	-0.11	0.04	-0.04	0.08	-0.01
Phospholipids in M VLDL	M-P	-0.10	0.00	0.12	0.03	0.04	-0.01	0.04	0.04	-0.03	-0.11	0.10	-0.04	0.12	-0.01
Cholesterol in M VLDL	M-C	-0.08	0.00	0.14	0.03	0.05	-0.01	0.12	0.04	0.13	-0.12	0.15	-0.03	0.16	-0.01
Cholesteryl Esters in M VLDL	M-Este-E	-0.04	0.00	0.09	0.04	0.01	-0.01	0.11	0.05	0.07	-0.08	0.13	-0.01	0.13	0.00
Free Cholesterol in M VLDL	M-Chol-C	-0.10	0.00	0.14	0.03	0.05	-0.01	0.07	0.04	0.03	-0.12	0.12	-0.04	0.13	-0.01
Triglycerides in M VLDL	M-Tryg	-0.07	0.00	0.06	0.01	-0.02	-0.01	-0.05	0.02	-0.28	-0.12	-0.07	-0.04	0.00	-0.01

Table S3 Continued

**Small VLDL**																	
Conc. of S VLDL Particles	S-VLDL	-0.09	-0.01	0.11	0.02	0.03	-0.01	-0.02	0.03	-0.19	-0.12	-0.01	-0.05	0.02	-0.01
Total Lipids in S VLDL	S-L	-0.09	-0.01	0.11	0.02	0.03	-0.01	-0.02	0.03	-0.17	-0.12	0.01	-0.05	0.04	-0.01
Phospholipids in S VLDL	S-P	-0.11	0.00	0.13	0.03	0.04	-0.01	0.04	0.03	-0.05	-0.13	0.08	-0.05	0.10	-0.01
Cholesterol in S VLDL	S-C	-0.11	0.00	0.14	0.03	0.06	0.00	0.05	0.03	0.00	-0.13	0.08	-0.04	0.10	-0.01
Cholesteryl Esters in S VLDL	S-Este-E	-0.11	0.00	0.14	0.03	0.06	0.00	0.02	0.02	-0.05	-0.13	0.06	-0.04	0.07	-0.01
Free Cholesterol in S VLDL	S-Chol-C	-0.11	0.00	0.15	0.03	0.05	-0.01	0.09	0.03	0.06	-0.14	0.12	-0.04	0.13	-0.01
Triglycerides in S VLDL	S-Tryg	-0.05	0.00	0.04	0.01	0.00	-0.02	-0.09	0.02	-0.30	-0.12	-0.10	-0.04	-0.03	-0.01
**Very Small VLDL**																	
Conc. of XS VLDL Particles	XS-VLDL	-0.08	-0.01	0.13	0.02	0.09	-0.01	0.02	0.02	0.06	-0.14	0.14	-0.03	0.14	-0.01
Total Lipids in XS VLDL	XS-L	-0.06	-0.01	0.12	0.02	0.10	-0.01	0.03	0.02	0.06	-0.14	0.14	-0.03	0.15	-0.01
Phospholipids in XS VLDL	XS-P	-0.06	-0.01	0.12	0.02	0.10	-0.01	0.01	0.02	0.01	-0.13	0.13	-0.03	0.13	-0.01
Cholesterol in XS VLDL	XS-C	-0.06	-0.01	0.11	0.03	0.10	-0.01	0.10	0.02	0.19	-0.14	0.17	-0.02	0.18	-0.01
Cholesteryl Esters in XS VLDL	XS-Este-E	-0.05	-0.01	0.09	0.03	0.10	-0.01	0.11	0.01	0.21	-0.14	0.17	-0.02	0.18	-0.01
Free Cholesterol in XS VLDL	XS-Chol-C	-0.08	-0.01	0.14	0.03	0.10	-0.01	0.06	0.02	0.11	-0.14	0.15	-0.03	0.16	-0.01
Triglycerides in XS VLDL	XS-Tryg	-0.04	-0.01	0.04	0.01	0.03	-0.01	-0.12	0.02	-0.22	-0.12	-0.03	-0.04	0.01	-0.01
**IDL Particles**																	
Conc. of IDL Particles	IDL-C	-0.07	0.00	0.15	0.03	0.07	-0.01	0.11	0.02	0.15	-0.15	0.20	-0.03	0.21	-0.01
Total Lipids in IDL	IDL-L	-0.04	0.00	0.12	0.02	0.06	-0.02	0.12	0.02	0.22	-0.15	0.20	-0.03	0.22	-0.01
Phospholipids in IDL	IDL-P	-0.03	0.00	0.11	0.02	0.06	-0.02	0.12	0.02	0.22	-0.15	0.18	-0.03	0.19	-0.01
Cholesterol in IDL	IDL-C	-0.04	0.00	0.10	0.02	0.05	-0.02	0.14	0.01	0.22	-0.15	0.19	-0.03	0.21	-0.01
Cholesteryl Esters in IDL	IDL-Este-E	-0.04	0.00	0.10	0.02	0.05	-0.02	0.14	0.01	0.21	-0.15	0.20	-0.03	0.22	-0.01
Free Cholesterol in IDL	IDL-Chol-C	-0.06	0.00	0.11	0.02	0.05	-0.02	0.13	0.01	0.26	-0.14	0.18	-0.02	0.18	-0.01
Triglycerides in IDL	IDL-Tryg	-0.05	-0.01	0.05	0.02	0.02	-0.01	-0.12	0.03	-0.14	-0.13	0.05	-0.05	0.07	-0.01
**LDL Particles, Large**																	
Conc. of L LDL Particles	L-LDL	-0.06	0.00	0.14	0.02	0.03	-0.01	0.14	0.04	0.20	-0.14	0.14	-0.03	0.17	-0.01
Total Lipids in L LDL	L-L	-0.07	0.00	0.13	0.02	0.02	-0.02	0.12	0.02	0.21	-0.17	0.16	-0.04	0.17	-0.01
Phospholipids in L LDL	L-P	-0.09	0.00	0.14	0.02	0.03	-0.02	0.13	0.02	0.20	-0.16	0.17	-0.03	0.18	-0.01
Cholesterol in L LDL	L-C	-0.06	0.00	0.13	0.02	0.02	-0.02	0.13	0.02	0.22	-0.16	0.16	-0.04	0.16	-0.01
Cholesteryl Esters in L LDL	L-Este-E	-0.06	0.00	0.12	0.01	0.02	-0.02	0.12	0.02	0.20	-0.17	0.15	-0.04	0.16	-0.01
Free Cholesterol in L LDL	L-Chol-C	-0.05	0.00	0.12	0.02	0.02	-0.02	0.15	0.01	0.26	-0.15	0.17	-0.03	0.16	-0.01
Triglycerides in L LDL	L-Tryg	-0.08	0.00	0.06	0.02	-0.01	-0.01	-0.12	0.04	-0.13	-0.13	0.07	-0.05	0.09	-0.02

Table S3 Continued

**Medium LDL**															
Conc. of Medium LDL Particles	M-LDL	-0.11	0.00	0.17	0.02	0.02	-0.02	0.05	0.02	0.05	-0.15	0.08	-0.03	0.10	-0.01
Total Lipids in M LDL	M-L	-0.10	0.00	0.14	0.02	0.00	-0.01	0.07	0.03	0.08	-0.15	0.11	-0.04	0.14	-0.01
Phospholipids in M LDL	M-P	-0.09	0.00	0.14	0.02	0.00	-0.01	0.08	0.03	0.09	-0.16	0.12	-0.04	0.14	-0.01
Cholesterol in M LDL	M-C	-0.10	0.00	0.14	0.02	0.01	-0.01	0.08	0.03	0.10	-0.15	0.12	-0.04	0.14	-0.01
Cholesteryl Esters in M LDL	M-Este-E	-0.11	0.00	0.14	0.02	0.00	-0.01	0.05	0.03	0.05	-0.15	0.10	-0.04	0.13	-0.01
Free Cholesterol in M LDL	M-Chol-C	-0.08	0.01	0.15	0.02	0.01	-0.02	0.14	0.02	0.21	-0.16	0.14	-0.03	0.14	-0.01
Triglycerides in M LDL	M-Tryg	-0.09	0.00	0.06	0.02	-0.03	-0.01	-0.11	0.04	-0.18	-0.12	0.02	-0.05	0.06	-0.01
**Small LDL**															
Conc. of S LDL Particles	S-LDL	-0.08	0.00	0.15	0.02	0.03	-0.01	0.04	0.03	0.04	-0.13	0.09	-0.04	0.10	-0.01
Total Lipids in S LDL	S-L	-0.07	0.00	0.16	0.02	0.01	-0.01	0.07	0.03	0.08	-0.15	0.12	-0.04	0.14	-0.01
Phospholipids in S LDL	S-P	-0.03	0.01	0.18	0.02	0.03	-0.02	0.09	0.03	0.12	-0.16	0.12	-0.04	0.14	-0.01
Cholesterol in S LDL	S-C	-0.08	0.00	0.15	0.02	0.00	-0.01	0.09	0.03	0.11	-0.15	0.12	-0.04	0.14	-0.01
Cholesteryl Esters in S LDL	S-Este-E	-0.09	0.00	0.14	0.02	-0.01	-0.01	0.07	0.03	0.05	-0.14	0.11	-0.04	0.14	-0.01
Free Cholesterol in S LDL	S-Chol-C	-0.07	0.01	0.17	0.02	0.02	-0.02	0.14	0.02	0.22	-0.15	0.13	-0.03	0.12	-0.01
Triglycerides in S LDL	S-Tryg	-0.08	-0.01	0.04	0.01	-0.02	-0.01	-0.09	0.03	-0.21	-0.11	-0.04	-0.04	0.03	-0.01
**HDL Particles, Extra Large**															
Conc. of XL HDL Particles	XL-HDL	0.06	0.02	-0.11	0.02	-0.01	-0.01	0.10	0.00	0.40	-0.15	0.13	-0.02	0.10	-0.01
Total Lipids in XL HDL	XL-L	0.08	0.01	-0.13	0.02	-0.01	-0.01	0.10	0.00	0.38	-0.14	0.12	-0.02	0.10	-0.01
Phospholipids in XL HDL	XL-P	0.12	0.01	-0.14	0.02	-0.01	0.00	0.06	0.01	0.31	-0.12	0.11	-0.02	0.09	-0.01
Cholesterol in XL HDL	XL-C	0.05	0.02	-0.13	0.02	-0.01	-0.01	0.13	0.00	0.39	-0.14	0.11	-0.02	0.09	-0.01
Cholesteryl Esters in XL HDL	XL-Este-E	0.06	0.02	-0.14	0.02	-0.02	-0.01	0.13	0.00	0.36	-0.14	0.11	-0.02	0.07	-0.01
Free Cholesterol in XL HDL	XL-Chol-C	0.05	0.02	-0.08	0.02	0.01	-0.01	0.09	-0.01	0.39	-0.14	0.11	-0.02	0.11	-0.01
Triglycerides in XL HDL	XL-Tryg	0.06	-0.01	0.01	0.00	0.01	-0.02	-0.12	0.02	-0.11	-0.10	0.06	-0.04	0.07	-0.02
**Large HDL**															
Conc. of Large HDL Particles	L-HDL	0.13	0.01	-0.09	0.01	0.03	-0.03	0.10	0.00	0.36	-0.15	0.18	-0.04	0.10	-0.01
Total Lipids in L HDL	L-L	0.13	0.01	-0.09	0.01	0.03	-0.02	0.08	0.00	0.41	-0.17	0.20	-0.04	0.13	-0.02
Phospholipids in L HDL	L-P	0.17	0.01	-0.04	0.03	0.05	-0.01	0.04	0.00	0.44	-0.18	0.23	-0.04	0.17	-0.01
Cholesterol in L HDL	L-C	0.12	0.01	-0.11	0.01	0.03	-0.02	0.10	0.01	0.35	-0.15	0.18	-0.04	0.09	-0.01
Cholesteryl Esters in L HDL	L-Este-E	0.11	0.01	-0.12	0.01	0.02	-0.02	0.11	0.01	0.33	-0.15	0.17	-0.04	0.07	-0.01
Free Cholesterol in L HDL	L-Chol-C	0.17	0.02	-0.04	0.02	0.08	-0.02	0.05	-0.01	0.40	-0.14	0.19	-0.04	0.15	-0.01
Triglycerides in L HDL	L-Tryg	0.10	-0.01	-0.02	-0.01	0.03	-0.02	-0.10	-0.01	-0.06	-0.14	0.07	-0.04	0.07	-0.02

Table S3 Continued

**Medium HDL**															
Conc. of M HDL Particles	M-HDL	0.05	0.01	-0.06	0.00	-0.04	-0.02	0.06	-0.02	0.14	-0.22	0.16	-0.04	0.13	-0.03
Total Lipids in M HDL	M-L	0.03	0.01	-0.06	0.00	-0.03	-0.03	0.03	-0.02	0.09	-0.22	0.15	-0.04	0.13	-0.03
Phospholipids in M HDL	M-P	0.02	0.01	-0.06	0.00	-0.01	-0.03	0.00	-0.02	0.02	-0.22	0.13	-0.04	0.14	-0.03
Cholesterol in M HDL	M-C	0.04	0.01	-0.07	0.00	-0.04	-0.02	0.09	-0.01	0.22	-0.21	0.18	-0.04	0.12	-0.02
Cholesteryl Esters in M HDL	M-Este-E	0.03	0.01	-0.08	0.00	-0.05	-0.02	0.09	-0.01	0.22	-0.20	0.18	-0.04	0.12	-0.02
Free Cholesterol in M HDL	M-Chol-C	0.07	0.01	-0.03	-0.01	-0.02	-0.03	0.07	-0.02	0.21	-0.23	0.17	-0.05	0.14	-0.03
Triglycerides in M HDL	M-Tryg	-0.02	0.00	-0.04	0.00	0.02	-0.02	-0.15	0.00	-0.30	-0.14	-0.04	-0.03	0.01	-0.01
**Small HDL**															
Conc. of S HDL Particles	S-HDL	-0.12	0.01	0.04	0.01	-0.02	-0.01	0.06	-0.01	-0.04	-0.18	0.06	-0.04	0.09	-0.03
Total Lipids in S HDL	S-L	-0.11	0.01	0.00	0.01	-0.01	-0.02	0.00	-0.01	-0.10	-0.18	0.04	-0.04	0.10	-0.03
Phospholipids in S HDL	S-P	-0.10	0.01	-0.01	0.01	0.00	-0.02	-0.02	-0.02	-0.12	-0.18	0.05	-0.04	0.11	-0.03
Cholesterol in S HDL	S-C	-0.13	0.01	0.03	0.01	-0.03	-0.01	0.08	-0.02	0.00	-0.19	0.07	-0.04	0.10	-0.03
Cholesteryl Esters in S HDL	S-Este-E	-0.14	0.01	0.02	0.01	-0.03	-0.01	0.09	-0.02	-0.01	-0.17	0.05	-0.04	0.08	-0.03
Free Cholesterol in S HDL	S-Chol-C	-0.08	0.00	0.04	0.00	-0.02	-0.02	0.03	0.00	0.02	-0.21	0.12	-0.05	0.15	-0.02
Triglycerides in S HDL	S-Tryg	-0.03	0.00	0.05	0.01	0.01	-0.01	-0.17	0.01	-0.36	-0.13	-0.10	-0.03	-0.04	-0.01
**VLDL Ratios, Extra Large (%)**															
Phospholipids:Tot. Lipids	P:L-XXL	0.05	0.01	0.01	0.01	0.04	-0.01	-0.08	-0.02	-0.16	-0.10	-0.09	-0.02	-0.03	-0.01
Cholesterol:Tot. Lipids	C:L-XXL	-0.03	0.02	0.06	0.01	0.03	-0.01	0.19	-0.02	0.42	-0.12	0.14	-0.02	0.09	-0.01
Cholesteryl Esters:Tot. Lipids	CE:L-XXL	-0.05	0.02	0.05	0.01	0.00	-0.01	0.18	-0.02	0.31	-0.13	0.14	-0.03	0.09	-0.02
Free Cholesterol:Tot. Lipids	CholC:L-XXL	0.02	0.02	0.06	0.01	0.05	-0.01	0.18	-0.02	0.34	-0.12	0.11	-0.02	0.06	-0.01
Triglicerydes:Tot. Lipids	Tryg:L-XXL	0.04	0.02	-0.07	-0.01	-0.15	-0.04	-0.11	-0.02	-0.54	-0.23	-0.05	0.00	-0.02	0.01
**Very Large VLDL Ratios (%)**															
Phospholipids:Tot. Lipids	P:L-XL	0.00	0.01	0.05	0.00	0.04	0.00	0.04	-0.03	-0.12	-0.05	0.00	-0.04	0.00	-0.03
Cholesterol:Tot. Lipids	C:L-XL	-0.06	0.02	0.07	0.01	0.03	-0.02	0.20	-0.01	0.37	-0.12	0.16	-0.02	0.07	-0.01
Cholesteryl Esters:Tot. Lipids	CE:L-XL	-0.05	0.02	0.06	0.01	0.03	-0.02	0.19	0.00	0.33	-0.11	0.16	-0.02	0.06	-0.01
Free Cholesterol:Tot. Lipids	CholC:L-XL	-0.07	0.02	0.06	0.01	0.02	-0.01	0.20	-0.01	0.44	-0.15	0.16	-0.02	0.10	-0.01
Triglicerydes:Tot. Lipids	Tryg:L-XL	0.10	0.03	-0.10	0.01	-0.05	-0.02	-0.23	-0.01	-0.45	-0.15	-0.15	-0.02	-0.09	-0.01
**Large VLDL Ratios (%)**															
Phospholipids:Tot. Lipids	P:L-L	0.03	0.01	0.02	0.01	0.02	-0.01	-0.22	-0.03	-0.27	-0.16	-0.12	-0.03	-0.11	-0.02
Cholesterol:Tot. Lipids	C:L-L	-0.06	0.02	0.06	0.01	0.05	-0.01	0.18	-0.01	0.47	-0.15	0.14	-0.02	0.07	-0.01
Cholesteryl Esters:Tot. Lipids	CE:L-L	-0.03	0.02	0.03	0.01	0.04	-0.01	0.18	0.00	0.39	-0.13	0.15	-0.02	0.06	-0.01
Free Cholesterol:Tot. Lipids	CholC:L-L	-0.08	0.00	0.05	0.01	0.01	-0.01	0.03	-0.02	-0.02	-0.19	0.01	-0.02	-0.02	-0.02
Triglicerydes:Tot. Lipids	Tryg:L-L	0.08	0.01	-0.06	0.01	-0.05	-0.01	-0.14	-0.02	-0.30	-0.17	-0.10	-0.03	-0.06	-0.02

Table S3 Continued

**Medium VLDL Ratios (%)**															
Phospholipids:Tot. Lipids	P:L-M	-0.02	0.01	0.03	0.02	0.04	-0.02	0.08	0.02	0.24	-0.13	0.13	-0.02	0.07	-0.01
Cholesterol:Tot. Lipids	C:L-M	0.00	0.02	-0.02	0.02	0.00	-0.02	0.06	0.02	0.15	-0.11	0.08	-0.02	0.00	0.00
Cholesteryl Esters:Tot. Lipids	CE:L-M	0.07	0.03	-0.11	0.01	-0.09	-0.04	-0.02	0.02	-0.08	-0.10	-0.02	-0.03	-0.09	-0.01
Free Cholesterol:Tot. Lipids	CholC:L-M	-0.03	0.01	0.02	0.02	0.03	-0.02	0.09	0.01	0.25	-0.12	0.11	-0.02	0.03	-0.01
Triglicerydes:Tot. Lipids	Tryg:L-M	0.03	0.02	-0.04	0.01	-0.04	-0.02	-0.14	0.00	-0.38	-0.13	-0.14	-0.02	-0.07	-0.01
**Small VLDL Ratios (%)**															
Phospholipids:Tot. Lipids	P:L-S	-0.03	0.01	0.06	0.01	0.01	-0.02	0.14	0.00	0.25	-0.11	0.13	-0.02	0.07	0.00
Cholesterol:Tot. Lipids	C:L-S	-0.07	0.01	0.06	0.02	0.03	-0.02	0.13	0.00	0.27	-0.12	0.12	-0.02	0.06	-0.01
Cholesteryl Esters:Tot. Lipids	CE:L-S	-0.10	0.01	0.07	0.02	0.04	-0.01	0.12	-0.01	0.31	-0.14	0.10	-0.02	0.06	-0.01
Free Cholesterol:Tot. Lipids	CholC:L-S	-0.03	0.02	0.04	0.01	0.01	-0.02	0.13	0.00	0.21	-0.11	0.11	-0.02	0.04	0.00
Triglicerydes:Tot. Lipids	Tryg:L-S	0.06	0.01	-0.08	0.01	-0.03	-0.02	-0.16	-0.01	-0.38	-0.13	-0.13	-0.02	-0.08	-0.01
**Extra Small VLDL Ratios (%)**															
Phospholipids:Tot. Lipids	P:L-XS	0.01	0.00	0.09	0.01	0.03	-0.02	-0.08	0.00	-0.38	-0.16	-0.06	-0.03	-0.04	-0.01
Cholesterol:Tot. Lipids	C:L-XS	0.02	0.01	-0.06	0.01	0.02	-0.02	0.10	0.02	0.12	-0.10	0.06	-0.02	-0.01	0.00
Cholesteryl Esters:Tot. Lipids	CE:L-XS	0.03	0.01	-0.09	0.01	0.03	-0.02	0.08	0.02	0.10	-0.10	0.05	-0.02	-0.01	0.00
Free Cholesterol:Tot. Lipids	CholC:L-XS	-0.08	0.00	0.11	0.01	0.04	-0.02	0.16	-0.01	0.18	-0.10	0.07	-0.01	0.00	0.00
Triglicerydes:Tot. Lipids	Tryg:L-XS	0.00	0.01	0.00	0.01	-0.04	-0.02	-0.15	0.00	-0.26	-0.11	-0.09	-0.02	-0.05	0.00
**ILDL Ratios (%)**															
Phospholipids:Tot. Lipids	P:L	0.08	0.01	-0.04	0.02	0.00	-0.01	-0.03	-0.02	-0.13	-0.16	-0.13	-0.02	-0.19	-0.01
Cholesterol:Tot. Lipids	C:L	-0.03	0.01	-0.03	0.02	0.02	-0.03	0.14	0.01	0.13	-0.10	0.09	-0.02	0.06	0.00
Cholesteryl Esters:Tot. Lipids	CE:L	-0.01	0.00	-0.04	0.02	0.02	-0.03	0.15	0.00	0.13	-0.12	0.11	-0.02	0.12	0.00
Free Cholesterol:Tot. Lipids	CholC:L	-0.07	0.00	0.02	0.01	0.01	-0.02	0.09	0.00	0.23	-0.11	0.01	-0.02	-0.04	0.00
Triglicerydes:Tot. Lipids	Tryg:L	0.00	0.01	0.00	0.01	-0.02	-0.02	-0.16	0.01	-0.20	-0.11	-0.09	-0.02	-0.04	0.00
**LDL Ratios Large (%)**															
Phospholipids:Tot. Lipids	P:L-L	-0.13	0.02	0.04	0.01	0.05	-0.02	0.03	0.01	-0.06	-0.14	-0.02	-0.04	0.01	-0.02
Cholesterol:Tot. Lipids	C:L-L	0.04	0.01	0.01	0.01	-0.02	-0.02	0.17	-0.01	0.26	-0.12	0.07	-0.02	0.01	-0.01
Cholesteryl Esters:Tot. Lipids	CE:L-L	0.03	0.01	0.01	0.01	-0.01	-0.02	0.09	0.00	0.08	-0.17	-0.02	-0.04	-0.02	-0.02
Free Cholesterol:Tot. Lipids	CholC:L-L	0.01	0.01	-0.08	0.02	0.01	-0.03	0.09	0.02	0.17	-0.10	0.07	-0.02	-0.01	0.00
Triglicerydes:Tot. Lipids	Tryg:L-L	0.01	0.01	-0.02	0.01	-0.02	-0.02	-0.19	0.00	-0.24	-0.12	-0.07	-0.02	-0.02	0.00
**Medium LDL Ratios (%)**															
Phospholipids:Tot. Lipids	P:L-M	0.03	0.01	-0.05	0.01	-0.01	-0.01	0.13	-0.02	0.24	-0.18	0.04	-0.02	0.04	-0.01
Cholesterol:Tot. Lipids	C:L-M	-0.07	0.01	0.04	0.02	0.03	-0.03	0.10	0.00	0.16	-0.11	0.03	-0.01	0.00	0.00
Cholesteryl Esters:Tot. Lipids	CE:L-M	-0.07	0.00	0.07	0.01	-0.01	-0.01	-0.11	-0.01	-0.34	-0.15	-0.06	-0.02	-0.02	-0.01

Table S3 Continued

Free Cholesterol:Tot. Lipids	CholC:L-M	0.04	0.01	-0.03	0.01	0.03	-0.03	0.15	0.01	0.28	-0.12	0.07	-0.02	-0.02	-0.01
Triglicerydes:Tot. Lipids	Tryg:L-M	0.06	0.01	-0.05	0.01	-0.03	-0.02	-0.18	0.00	-0.23	-0.11	-0.08	-0.02	-0.03	0.00
**Small LDL Ratios (%)**															
Phospholipids:Tot. Lipids	P:L-S	0.11	0.00	0.02	0.01	0.08	-0.01	0.05	-0.01	0.32	-0.15	-0.02	-0.02	0.00	-0.02
Cholesterol:Tot. Lipids	C:L-S	-0.15	0.00	0.02	0.01	-0.04	-0.01	0.17	-0.01	0.23	-0.12	0.11	-0.02	0.10	-0.01
Cholesteryl Esters:Tot. Lipids	CE:L-S	-0.10	0.00	-0.01	0.01	-0.07	-0.01	-0.03	-0.01	-0.30	-0.15	0.03	-0.02	0.04	-0.02
Free Cholesterol:Tot. Lipids	CholC:L-S	0.00	0.01	0.02	0.01	0.04	-0.03	0.11	0.01	0.28	-0.12	0.04	-0.02	-0.03	-0.01
Triglicerydes:Tot. Lipids	Tryg:L-S	0.02	0.01	-0.06	0.01	-0.01	-0.02	-0.16	0.00	-0.27	-0.11	-0.11	-0.03	-0.04	-0.01
**HDL Ratios, Very Large (%)**															
Phospholipids:Tot. Lipids	P:L-XL	0.22	-0.02	-0.11	0.03	-0.02	0.01	-0.03	0.04	0.15	-0.07	0.08	-0.02	0.06	0.00
Cholesterol:Tot. Lipids	C:L-XL	-0.17	-0.02	0.09	0.01	-0.03	-0.01	0.11	0.01	-0.01	-0.10	-0.08	-0.02	-0.07	-0.01
Cholesteryl Esters:Tot. Lipids	CE:L-XL	-0.04	0.00	0.04	-0.01	-0.08	-0.02	0.11	0.01	0.15	-0.12	0.02	-0.03	-0.01	-0.01
Free Cholesterol:Tot. Lipids	CholC:L-XL	-0.16	0.00	0.15	0.01	0.02	-0.01	-0.07	0.01	-0.28	-0.13	-0.13	-0.03	-0.07	-0.01
Triglicerydes:Tot. Lipids	Tryg:L-XL	-0.10	0.00	0.04	0.01	0.03	-0.01	-0.13	0.02	-0.29	-0.11	-0.07	-0.03	-0.03	-0.01
**Large HDL Ratios (%)**															
Phospholipids:Tot. Lipids	P:L-L	-0.28	0.04	-0.21	0.06	-0.10	0.06	0.04	-0.02	-0.46	-0.08	-0.22	0.03	-0.19	0.06
Cholesterol:Tot. Lipids	C:L-L	0.13	0.00	-0.04	-0.01	-0.04	-0.02	0.00	0.02	0.10	-0.11	0.01	-0.03	-0.12	0.00
Cholesteryl Esters:Tot. Lipids	CE:L-L	0.12	0.00	-0.03	-0.01	-0.08	-0.02	0.01	0.03	0.06	-0.12	0.02	-0.03	-0.13	0.00
Free Cholesterol:Tot. Lipids	CholC:L-L	0.29	0.01	0.14	0.01	0.22	-0.03	-0.09	0.00	0.34	-0.05	0.18	-0.04	0.22	-0.02
Triglicerydes:Tot. Lipids	Tryg:L-L	-0.09	-0.01	-0.01	0.00	0.01	-0.02	-0.13	0.01	-0.38	-0.14	-0.12	-0.04	-0.08	-0.01
**Medium HDL Ratios (%)**															
Phospholipids:Tot. Lipids	P:L-M	-0.05	0.00	0.04	0.00	0.08	-0.02	-0.17	0.01	-0.42	-0.14	-0.15	-0.02	0.01	0.00
Cholesterol:Tot. Lipids	C:L-M	0.05	0.00	-0.04	0.01	-0.06	-0.02	0.14	0.02	0.35	-0.14	0.14	-0.03	0.02	-0.01
Cholesteryl Esters:Tot. Lipids	CE:L-M	0.05	-0.01	-0.03	0.01	-0.06	-0.02	0.12	0.02	0.29	-0.13	0.10	-0.03	-0.01	0.00
Free Cholesterol:Tot. Lipids	CholC:L-M	0.16	0.01	0.03	-0.01	0.01	-0.04	0.13	-0.01	0.41	-0.20	0.14	-0.03	0.11	-0.02
Triglicerydes:Tot. Lipids	Tryg:L-M	0.00	0.00	0.02	0.01	0.04	-0.02	-0.18	0.00	-0.42	-0.14	-0.13	-0.03	-0.08	-0.01
**Small HDL Ratios (%)**															
Phospholipids:Tot. Lipids	P:L-S	0.11	0.00	-0.12	0.01	0.06	-0.01	-0.14	0.00	-0.23	-0.15	0.01	-0.03	0.04	-0.01
Cholesterol:Tot. Lipids	C:L-S	-0.05	0.01	0.07	0.00	-0.04	-0.02	0.17	0.00	0.33	-0.12	0.08	-0.02	0.01	0.00
Cholesteryl Esters:Tot. Lipids	CE:L-S	-0.09	0.00	0.04	0.00	-0.03	-0.02	0.16	0.00	0.25	-0.12	0.04	-0.03	0.00	-0.01
Free Cholesterol:Tot. Lipids	CholC:L-S	0.03	0.03	0.07	0.02	-0.02	-0.03	0.10	-0.01	0.59	-0.22	0.13	-0.02	0.10	-0.02
Triglicerydes:Tot. Lipids	Tryg:L-S	0.02	0.00	0.06	0.01	0.04	-0.02	-0.19	0.00	-0.39	-0.13	-0.15	-0.03	-0.09	-0.01

Table S4: Baseline characteristics of study population sub-groups with and without NMR-metabolites and MRI profiles

	All (n=487,577)		with NMR* (n=96,947)		with MRI** (n=24,639)		with NMR and PDFF*** (n=1088)	
	**No.**	**%**	**No.**	**%**	**No.**	**%**	**No.**	**%**
**Age (yrs. mean, SD)**	57	8.1	57	8.1	55	7.5	56	7.7
<55 yrs.	188,809	39%	37,275	38%	11,110	45%	460	42%
55+yrs.	298,768	61%	59,672	62%	13,529	55%	628	58%
**Ethnicity**								
White	460,275	94%	91,948	95%	23,931	97%	1057	97%
**Smoking**								
Never	267,260	55%	53,190	55%	14,940	61%	639	59%
Previous	169,090	35%	33,592	35%	8,125	33%	399	37%
Current	51,227	11%	10,165	10%	1,574	6%	50	5%
**Drinking**								
Never	38,749	8%	7,716	8%	1,199	5%	61	6%
Previous	110,504	23%	22,277	23%	4,734	19%	209	19%
Current	338,324	69%	66,954	69%	18,706	76%	818	75%
**Biological Measures (mmol/L, mean, SD)**[±]								
BMI (kg/m2)	27	4.8	28	4.7	27	4.2	27	4.3
FMI (kg/m2)	9	3.6	9	3.6	8	3.2	8	3.3
LMI (kg/m2)	19	2.6	19	2.6	19	2.5	18	2.5
**Medical historiesł**								
BMI ≥25 kg/m2	326,679	67%	66,295	68%	15,159	62%	677	62%
Any chronic disease	178,765	37%	35,092	36%	7,095	29%	486	45%
Diabetes	76,680	16%	12,401	13%	3,283	13%	101	9%
Vascular disease	200,257	41%	39,613	41%	8,295	34%	399	37%
Lipid-lowering medication	83,395	17%	15,901	16%	2,968	12%	164	15%

Exclusions as per footnote of Table 1, now in sub-sets with complete data on main confounders listed in in the table. *Restricted in addition to those with complete NMR-metabolite values. ** Without restriction to those with complete NMR-metabolites values (only 5,234 participants also had complete NMR data). ***As per main Table 1 in the manuscript.

Table S5: Predicted liver fat concentrations (PDFF) comparing conventional risk factors and NMR-metabolites

Predictive Models*	R^2 (95% CIs)***	change in R^2	dof	Chi^2	p-value for Chi^2
**Conventional Models (M)**					
M0: Age, sex, smoking, drinking	0.100 (-0.008-0.028)		9	14.0	0.051
M1: M0 + BMI only	0.135 (0.082-0.188)	12%	12	129.8	<0.001
M2: M1 + HDL-c + Tryg	0.203 (0.153-0.253)	7%	15	82.3	<0.001
M3: M2 + Diabetes	0.211 (0.159-0.262)	1%	16	10.1	0.002
M4: M3 + ALT	0.236 (0.186-0.287)	3%	17	32.1	<0.001
**Conventional+NMR Models****					
M5: M1 + PC1-PC10	0.278 (0.220-0.335)	15%	24	183.0	<0.001
M7: M2 + PC1-PC10	0.277 (0.226-0.327)	8%	27	107.3	<0.001
M6: M3 + PC1-PC11	0.280 (0.228-0.331)	7%	28	102.1	<0.001
M8: M4 + PC1-PC10	0.296 (0.238-0.353)	6%	29	92.7	<0.001

*All models are further restricted to 957 participants with complete information on biochemsitry of interest. Analyses of biochemistry are also adjusted for batch. BMI, HDL-c and Tryglicerides were considered as continuous increase per one SD, with biochemistry-measured biomarkers log-normalised (of credible values within 4 SDs).

**PC=NMR-predicted principal-components (see Table S6 for details). The first 10 PCs explained 90% of the initial variation of PDFF levels due to NMR-metabolites. PC1 was mostly composed by VLDL particles and explained 35% of the variation; PC2 was mostly composed by ratios of various lipids classes to total lipids in VLDL particles and explained an additional 26% of the variation; PC3 was mostly composed by HDL particles and explained an additional 12% of the variation; PC4 was composed of a mixture of VLDL, HDL and ratios; PCs 5 and 6 were composed of ratios of lipids in XL or small VLDLs and IDLs; PC7 was mostly composed from amino-acids; PCs 8-9 were mostly composed by fatty acids and some ratios of lipids in LDL and VLDL particles; PC10 was composed of renal function and amino-acids. PC4-PC10, together, explained 18% of the variation.

***Bootstrap estimates with 100 replications, shows the proportion of the variance in the outcome explained by the model, with higher R^2 values suggesting a better predicting ability. (Estimates were similar when the PCs were derived from the absolute measures without ratios.) The proprotion of change explained by various predicting models was calculated as the difference in R-squared statistic relative to the model for comparison. dof=degrees of freedom; BMI=Body Mass Index; HDL-c=High density lipoprotein choletserol; Tryg=Tryglicerides.

Table S6: Characterization of the variation in NMR-metabolites explained by the first 10 NMR-metabolic principal components (PC)

NMR-metabolites	PC1	PC2	PC3	PC4	PC5	PC6	PC7	PC8	PC9	PC10	Unexplained
**Cholesterol**											
Total Cholesterol	0.06	0.10	0.03	-0.01	-0.01	0.02	0.00	-0.02	0.00	0.00	0.00
Non HDL-C	0.08	0.08	-0.02	-0.01	-0.01	0.02	0.00	-0.03	-0.01	-0.01	0.00
Remnant Cholesterol	0.09	0.07	-0.02	0.03	-0.03	-0.01	0.02	-0.02	-0.03	-0.02	0.00
VLDL Cholesterol	0.10	0.03	-0.03	0.04	-0.02	-0.01	-0.01	0.02	0.00	0.00	0.01
Clinical LDL Cholesterol	0.07	0.09	-0.04	-0.03	-0.01	0.02	0.01	-0.03	-0.02	0.00	0.00
LDL Cholesterol	0.08	0.08	-0.02	-0.04	0.01	0.04	-0.01	-0.04	0.01	0.01	0.00
HDL Cholesterol	-0.03	0.08	0.12	-0.03	0.00	-0.03	-0.02	0.02	0.03	0.02	0.01
**Triglycerides**											
Total Triglycerides	0.10	-0.04	0.03	0.00	-0.03	0.02	-0.02	0.01	0.00	-0.01	0.01
Triglycerides in VLDL	0.10	-0.05	0.02	-0.01	-0.06	0.03	-0.03	0.03	0.00	0.00	0.01
Triglycerides in LDL	0.09	-0.01	0.05	0.04	0.09	0.03	0.01	-0.10	0.02	0.03	0.03
Triglycerides in HDL	0.08	-0.03	0.09	0.04	0.04	-0.08	0.02	0.00	-0.02	-0.06	0.04
**Phospholipids**											
Total Phospholipids in Lipoprotein Particles	0.06	0.08	0.09	-0.01	-0.01	-0.01	0.00	-0.03	-0.01	0.00	0.01
Phospholipids in VLDL	0.11	-0.01	-0.01	0.03	-0.02	0.00	-0.02	0.02	0.01	0.00	0.01
Phospholipids in LDL	0.08	0.08	-0.03	-0.04	0.01	0.03	0.01	-0.05	-0.01	0.00	0.00
Phospholipids in HDL	-0.01	0.06	0.16	-0.02	0.00	-0.05	0.00	0.00	0.00	0.00	0.01
**Cholesteryl esters**											
Total Esterified Cholesterol	0.06	0.10	0.03	-0.02	-0.01	0.02	0.00	-0.03	0.00	0.00	0.00
Cholesteryl Esters in VLDL	0.10	0.04	-0.04	0.05	-0.01	-0.02	0.00	0.02	0.00	0.00	0.01
Cholesteryl Esters in LDL	0.08	0.08	-0.02	-0.04	0.01	0.04	-0.01	-0.03	0.01	0.01	0.01
Cholesteryl Esters in HDL	-0.03	0.08	0.12	-0.04	0.00	-0.03	-0.02	0.03	0.03	0.02	0.01
**Free cholesterol**											
Total Free Cholesterol	0.07	0.09	0.02	0.01	-0.02	0.02	0.00	-0.01	0.00	0.00	0.00
Free Cholesterol in VLDL	0.11	0.01	-0.02	0.03	-0.02	0.00	-0.02	0.02	0.00	0.01	0.01
Free Cholesterol in LDL	0.06	0.10	-0.03	-0.04	0.00	0.04	0.01	-0.04	-0.01	-0.01	0.01
Free Cholesterol in HDL	-0.01	0.08	0.14	0.01	-0.01	-0.01	-0.01	-0.01	0.01	0.01	0.01
**Total lipids**											
Total Lipids in Lipoprotein Particles	0.09	0.06	0.05	-0.01	-0.02	0.02	-0.01	-0.03	0.00	0.01	0.01
Total Lipids in VLDL	0.11	-0.02	0.00	0.01	-0.04	0.02	-0.02	0.02	0.00	0.00	0.01
Total Lipids in LDL	0.08	0.08	-0.02	-0.03	0.01	0.04	0.00	-0.05	0.00	0.01	0.00
Total Lipids in HDL	-0.01	0.07	0.15	-0.02	0.00	-0.04	-0.01	0.00	0.01	0.01	0.01
**Lipoprotein concentrations**											
Total Concentration of Lipoprotein Particles	0.02	0.08	0.11	-0.10	0.03	-0.04	-0.01	0.00	0.01	0.01	0.01
Concentration of VLDL Particles	0.11	0.01	-0.01	0.04	0.00	0.01	-0.01	0.01	0.00	-0.01	0.01
Concentration of LDL Particles	0.09	0.07	-0.04	0.01	0.00	0.05	-0.01	-0.02	0.01	0.02	0.00
Concentration of HDL Particles	0.01	0.07	0.12	-0.11	0.03	-0.05	-0.01	0.00	0.01	0.01	0.01
Average Diameter for VLDL Particles	0.08	-0.07	-0.01	-0.07	-0.08	0.03	-0.05	0.05	0.03	0.04	0.04
Average Diameter for LDL Particles	-0.02	0.06	-0.01	0.02	0.06	0.12	-0.05	-0.01	0.16	-0.12	0.45
Average Diameter for HDL Particles	-0.05	0.06	0.11	0.10	-0.04	0.01	-0.01	0.02	0.03	0.03	0.02

Table S6: Continued

**Other lipids**											
Phosphoglycerides	0.06	0.07	0.12	-0.02	0.01	-0.02	0.00	-0.01	-0.01	-0.01	0.01
Phosphoglycerides:Tryglicerides Ratio	0.08	-0.08	-0.02	0.01	-0.04	0.04	-0.02	0.02	0.00	0.00	0.01
Total Cholines	0.05	0.08	0.11	-0.02	0.01	-0.02	0.00	-0.01	0.01	-0.01	0.01
Phosphatidylcholines	0.05	0.07	0.12	-0.01	0.01	-0.02	-0.01	-0.01	0.00	-0.05	0.02
Sphingomyelins	0.04	0.10	0.06	-0.02	0.02	0.02	0.02	-0.03	-0.01	0.05	0.04
**Apolipoproteins**											
Apolipoprotein B	0.09	0.07	-0.04	0.02	-0.01	0.03	0.00	-0.02	0.00	0.00	0.00
Apolipoprotein A1	0.00	0.07	0.15	-0.06	0.01	-0.06	-0.01	0.00	0.01	0.01	0.01
ApoB:ApoA Ratio	0.08	0.01	-0.12	0.05	-0.01	0.06	0.01	-0.02	-0.01	0.00	0.00
**Fatty acids**											
Total Fatty Acids	0.09	0.02	0.07	-0.01	0.00	0.02	-0.01	-0.01	0.01	0.04	0.03
Degree of Unsaturation	-0.04	0.07	0.00	0.02	0.13	0.10	-0.01	0.25	-0.11	-0.05	0.09
Omega-3 FA	0.04	0.02	0.07	0.03	0.16	0.15	0.00	0.17	-0.21	0.02	0.04
Omega-6 FA	0.08	0.07	0.05	-0.01	-0.02	0.01	-0.04	0.05	0.09	-0.03	0.08
Polyunsaturated FA	0.08	0.06	0.06	0.00	0.02	0.05	-0.04	0.09	0.02	-0.01	0.07
Monounsaturated FA	0.10	-0.01	0.06	-0.01	-0.02	0.00	-0.01	-0.04	0.05	0.06	0.05
Saturated FA	0.09	0.01	0.08	-0.01	-0.01	-0.01	0.01	-0.07	-0.04	0.06	0.02
Linoleic FA	0.07	0.06	0.04	0.00	-0.05	0.01	-0.05	0.04	0.14	-0.07	0.10
Docosahexaenoic FA	0.01	0.05	0.06	0.02	0.18	0.16	0.00	0.18	-0.19	-0.01	0.06
Omega-3: Total FA %	0.00	0.01	0.04	0.04	0.19	0.17	0.00	0.21	-0.26	0.01	0.04
Omega-6: Total FA %	-0.07	0.06	-0.07	-0.01	-0.03	-0.02	-0.04	0.10	0.13	-0.12	0.08
Polyunsaturated:Total FA %	-0.07	0.06	-0.05	0.01	0.05	0.06	-0.04	0.18	0.03	-0.11	0.06
Monounsaturated: Total FA %	0.07	-0.07	0.01	-0.01	-0.04	-0.04	0.00	-0.07	0.11	0.07	0.12
Saturated : Total FA%	0.04	-0.02	0.07	-0.01	-0.04	-0.08	0.08	-0.22	-0.19	0.11	0.25
Linoleic: Total FA%	-0.03	0.07	-0.05	0.01	-0.08	-0.01	-0.07	0.08	0.22	-0.17	0.19
Docosahexaenoic : Total FA%	-0.04	0.04	0.02	0.02	0.19	0.15	0.00	0.19	-0.20	-0.03	0.07
Polyunsaturated: Monounsaturated FA %	-0.07	0.07	-0.03	0.01	0.04	0.05	-0.02	0.12	-0.05	-0.10	0.07
Omega-6:Omega-3 FA 5	-0.02	0.00	-0.06	-0.04	-0.19	-0.16	-0.01	-0.17	0.27	-0.04	0.05
**Amino acids**											
Alanine	0.02	-0.02	0.03	-0.02	0.03	0.06	0.17	0.02	0.02	-0.07	0.74
Glutamine	0.00	0.02	0.01	-0.02	-0.01	0.07	0.17	0.07	0.16	-0.05	0.68
Glycine	-0.01	0.00	0.01	0.02	0.01	0.03	0.04	0.00	0.04	0.00	0.97
Histidine	0.01	0.00	0.02	-0.03	-0.04	0.06	0.21	0.06	0.10	-0.02	0.67
Tot. Conc. Branched-Chain AA	0.03	-0.03	0.02	-0.02	-0.01	0.08	0.36	0.08	0.08	-0.01	0.12
Isoleucine	0.03	-0.03	0.02	0.00	-0.03	0.07	0.34	0.09	0.09	0.00	0.21
Leucine	0.03	-0.03	0.01	-0.04	-0.03	0.07	0.35	0.08	0.09	-0.01	0.17
Valine	0.04	-0.03	0.03	-0.02	0.01	0.08	0.34	0.07	0.06	-0.01	0.18
Phenylalanine	0.01	-0.01	0.03	0.01	-0.03	0.07	0.25	0.02	0.11	-0.11	0.53
Tyrosine	0.02	-0.01	0.04	-0.02	0.00	0.06	0.27	0.03	0.05	-0.09	0.49

Table S6: Continued

**Glycolysis**											
Glucose	0.01	0.00	0.05	-0.03	0.01	0.00	0.11	0.05	0.04	0.05	0.82
Lactate	0.02	0.00	0.01	-0.06	0.07	0.01	0.04	-0.08	0.03	0.09	0.82
Pyruvate	0.02	0.01	0.02	-0.02	0.07	-0.01	0.07	-0.03	0.04	0.16	0.83
Citrate	0.01	0.01	0.03	0.00	0.02	0.00	0.11	0.04	0.17	0.18	0.69
**Ketone bodies**											
3-Hydroxybutyrate	0.01	0.01	-0.02	-0.03	0.02	0.00	0.03	0.03	0.05	0.43	0.48
Acetate	0.00	0.01	0.01	0.00	-0.04	-0.01	0.01	0.03	0.10	0.08	0.92
Acetoacetate	0.01	0.00	0.02	0.00	-0.02	-0.01	0.02	0.07	0.00	0.25	0.79
Acetone	-0.02	0.01	0.01	-0.02	-0.01	0.00	0.06	0.06	0.07	0.34	0.61
**Fluid balance**											
Creatinine	0.03	-0.03	-0.02	-0.04	0.03	0.05	0.17	0.05	0.06	0.11	0.59
Albumin	0.01	0.03	0.03	-0.08	0.07	0.00	0.09	0.12	0.00	0.05	0.69
**Inflammation**											
Glycoprotein Acetyls	0.07	-0.01	0.02	-0.04	0.05	0.02	0.05	0.03	0.03	0.10	0.51
**Lipoprotein concentrations**											
**VLDL particles, Extremely Large**											
Concentration of Extremely Large VLDL Particles	0.08	-0.06	0.01	0.02	-0.08	-0.06	-0.01	0.10	0.00	-0.02	0.07
Total Lipids in Extremely Large VLDL	0.08	-0.06	0.01	0.01	-0.12	-0.03	-0.01	0.07	-0.02	0.03	0.04
Phospholipids in Extremely Large VLDL	0.07	-0.06	0.01	0.02	-0.05	-0.08	-0.02	0.13	0.03	-0.05	0.15
Cholesterol in Extremely Large VLDL	0.09	-0.05	0.00	0.01	-0.06	-0.07	-0.02	0.09	0.03	0.02	0.04
Cholesteryl Esters in Extremely Large VLDL	0.09	-0.05	-0.01	0.01	-0.05	-0.07	-0.03	0.08	0.05	0.03	0.07
Free Cholesterol in Extremely Large VLDL	0.08	-0.06	0.01	0.02	-0.06	-0.06	-0.02	0.10	0.01	0.01	0.05
Triglycerides in Extremely Large VLDL	0.07	-0.06	0.02	0.00	-0.18	0.00	-0.01	0.07	-0.05	0.06	0.12
**VLDL particles, Very Large**											
Concentration of Very Large VLDL Particles	0.10	-0.05	-0.01	-0.01	-0.04	-0.01	-0.03	0.05	0.01	-0.02	0.02
Total Lipids in Very Large VLDL	0.10	-0.05	-0.01	-0.01	-0.06	0.00	-0.03	0.05	0.00	-0.02	0.01
Phospholipids in Very Large VLDL	0.10	-0.04	-0.02	0.01	-0.05	-0.02	-0.02	0.06	0.01	-0.02	0.03
Cholesterol in Very Large VLDL	0.10	-0.02	-0.03	0.01	-0.04	-0.02	-0.02	0.05	0.02	0.00	0.02
Cholesteryl Esters in Very Large VLDL	0.10	-0.01	-0.05	0.00	-0.02	-0.02	-0.02	0.05	0.03	0.01	0.03
Free Cholesterol in Very Large VLDL	0.10	-0.04	-0.02	0.01	-0.05	-0.01	-0.02	0.06	0.01	0.00	0.02
Triglycerides in Very Large VLDL	0.09	-0.06	0.01	-0.02	-0.07	0.01	-0.03	0.05	0.00	-0.03	0.02
**VLDL particles, Large**											
Concentration of Large VLDL Particles	0.10	-0.04	-0.01	-0.01	-0.04	0.02	-0.03	0.04	0.01	-0.01	0.01
Total Lipids in Large VLDL	0.10	-0.04	-0.01	-0.02	-0.05	0.03	-0.04	0.05	0.00	-0.02	0.01
Phospholipids in Large VLDL	0.10	-0.04	-0.02	-0.01	-0.03	-0.01	-0.03	0.06	0.02	-0.04	0.02
Cholesterol in Large VLDL	0.10	-0.02	-0.03	0.00	-0.02	0.00	-0.03	0.06	0.01	-0.01	0.01
Cholesteryl Esters in Large VLDL	0.10	-0.01	-0.04	0.02	-0.02	-0.01	-0.03	0.07	0.02	-0.02	0.02
Free Cholesterol in Large VLDL	0.10	-0.03	-0.02	-0.01	-0.03	0.00	-0.03	0.05	0.01	-0.01	0.01
Triglycerides in Large VLDL	0.09	-0.04	0.01	-0.04	-0.08	0.06	-0.04	0.04	-0.01	-0.01	0.01

Table S6: Continued

**VLDL particles, Medium**											
Concentration of Medium VLDL Particles	0.10	0.03	-0.03	0.00	-0.02	0.03	-0.03	0.03	0.01	0.01	0.01
Total Lipids in Medium VLDL	0.10	0.02	-0.02	0.00	-0.03	0.04	-0.04	0.03	0.02	-0.01	0.01
Phospholipids in Medium VLDL	0.10	0.04	-0.03	0.02	-0.02	0.02	-0.02	0.02	0.02	-0.01	0.01
Cholesterol in Medium VLDL	0.08	0.07	-0.05	0.03	-0.02	0.01	-0.01	0.03	0.01	-0.01	0.02
Cholesteryl Esters in Medium VLDL	0.06	0.09	-0.06	0.04	-0.02	0.00	0.00	0.03	0.01	-0.01	0.03
Free Cholesterol in Medium VLDL	0.10	0.05	-0.04	0.02	-0.02	0.02	-0.02	0.03	0.01	0.00	0.01
Triglycerides in Medium VLDL	0.10	-0.03	0.01	-0.03	-0.03	0.06	-0.05	0.04	0.02	-0.02	0.01
**VLDL particles, Small**											
Concentration of Small VLDL Particles	0.10	0.00	-0.01	0.02	0.04	0.03	-0.03	0.03	0.03	-0.02	0.01
Total Lipids in Small VLDL	0.11	0.00	-0.01	0.02	0.03	0.02	-0.03	0.03	0.02	-0.02	0.01
Phospholipids in Small VLDL	0.10	0.03	-0.03	0.02	0.02	0.02	-0.02	0.02	0.02	-0.01	0.01
Cholesterol in Small VLDL	0.10	0.04	-0.05	0.03	0.04	-0.01	-0.01	0.01	0.01	-0.01	0.01
Cholesteryl Esters in Small VLDL	0.10	0.03	-0.05	0.04	0.05	-0.03	0.00	0.01	0.01	-0.01	0.01
Free Cholesterol in Small VLDL	0.09	0.05	-0.04	0.02	0.02	0.01	-0.01	0.01	0.01	-0.01	0.01
Triglycerides in Small VLDL	0.10	-0.04	0.03	0.01	0.03	0.04	-0.04	0.05	0.03	-0.04	0.03
**VLDL particles, Very Small**											
Concentration of Very Small VLDL Particles	0.09	0.05	0.00	0.10	0.03	-0.03	0.03	-0.03	-0.04	-0.03	0.02
Total Lipids in Very Small VLDL	0.08	0.05	0.01	0.11	0.03	-0.03	0.03	-0.02	-0.03	-0.03	0.02
Phospholipids in Very Small VLDL	0.09	0.04	0.01	0.13	0.03	-0.02	0.02	-0.02	-0.03	-0.02	0.03
Cholesterol in Very Small VLDL	0.07	0.08	0.00	0.11	-0.01	-0.05	0.04	-0.01	-0.06	-0.04	0.02
Cholesteryl Esters in Very Small VLDL	0.06	0.08	0.00	0.10	-0.02	-0.05	0.04	-0.01	-0.06	-0.05	0.02
Free Cholesterol in Very Small VLDL	0.08	0.06	0.00	0.11	0.02	-0.03	0.03	-0.01	-0.05	-0.02	0.03
Triglycerides in Very Small VLDL	0.09	-0.03	0.05	0.07	0.09	0.00	0.00	-0.02	0.01	-0.03	0.03
**IDL Particles**											
Concentration of IDL Particles	0.07	0.08	-0.03	0.04	-0.03	-0.03	0.06	-0.06	-0.06	-0.05	0.04
Total Lipids in IDL	0.06	0.10	0.01	0.03	-0.02	-0.01	0.04	-0.05	-0.04	-0.05	0.01
Phospholipids in IDL	0.06	0.10	0.00	0.06	0.00	0.00	0.02	-0.03	-0.02	-0.03	0.01
Cholesterol in IDL	0.05	0.10	0.00	0.01	-0.03	-0.01	0.04	-0.04	-0.05	-0.06	0.01
Cholesteryl Esters in IDL	0.05	0.10	0.00	0.01	-0.04	-0.01	0.04	-0.05	-0.05	-0.07	0.02
Free Cholesterol in IDL	0.05	0.11	-0.01	0.02	-0.01	-0.02	0.04	-0.03	-0.06	0.00	0.02
Triglycerides in IDL	0.09	-0.01	0.06	0.08	0.10	0.00	0.02	-0.08	0.01	-0.01	0.03
**LDL Particles, Large**											
Concentration of Large LDL Particles	0.08	0.07	-0.04	0.02	0.01	0.07	-0.02	-0.02	0.04	0.00	0.02
Total Lipids in Large LDL	0.07	0.09	-0.01	-0.03	0.02	0.04	0.00	-0.05	0.01	-0.01	0.01
Phospholipids in Large LDL	0.07	0.09	-0.03	-0.03	0.00	0.01	0.02	-0.04	-0.02	-0.01	0.00
Cholesterol in Large LDL	0.07	0.09	-0.02	-0.03	0.01	0.05	-0.01	-0.04	0.01	-0.01	0.01
Cholesteryl Esters in Large LDL	0.07	0.09	-0.01	-0.03	0.02	0.06	-0.01	-0.04	0.02	-0.01	0.01
Free Cholesterol in Large LDL	0.05	0.10	-0.02	-0.03	-0.01	0.03	0.01	-0.04	-0.01	-0.01	0.01
Triglycerides in Large LDL	0.09	0.00	0.05	0.05	0.11	0.02	0.02	-0.12	0.01	0.02	0.04

Table S6: Continued

**LDL Particles, Medium**											
Concentration of Medium LDL Particles	0.09	0.05	-0.04	-0.01	-0.01	0.00	0.00	0.01	-0.04	0.06	0.05
Total Lipids in Medium LDL	0.09	0.06	-0.03	-0.05	0.02	0.03	-0.01	-0.03	0.00	0.02	0.01
Phospholipids in Medium LDL	0.09	0.06	-0.04	-0.06	0.02	0.05	0.00	-0.05	0.01	0.00	0.01
Cholesterol in Medium LDL	0.09	0.06	-0.04	-0.05	0.01	0.03	-0.01	-0.02	-0.01	0.03	0.01
Cholesteryl Esters in Medium LDL	0.09	0.05	-0.03	-0.05	0.01	0.02	-0.01	-0.01	-0.01	0.03	0.02
Free Cholesterol in Medium LDL	0.07	0.09	-0.05	-0.06	0.01	0.05	0.00	-0.04	0.00	0.00	0.01
Triglycerides in Medium LDL	0.10	-0.02	0.04	0.02	0.08	0.04	-0.01	-0.09	0.03	0.04	0.03
**LDL Particles, Small**											
Concentration of Small LDL Particles	0.09	0.05	-0.04	0.02	-0.02	0.04	-0.01	-0.03	-0.01	0.04	0.03
Total Lipids in Small LDL	0.09	0.06	-0.04	-0.02	0.00	0.07	-0.01	-0.05	0.01	0.03	0.01
Phospholipids in Small LDL	0.07	0.07	-0.05	0.00	0.00	0.12	-0.01	-0.09	0.00	0.01	0.03
Cholesterol in Small LDL	0.08	0.06	-0.05	-0.03	-0.01	0.05	-0.01	-0.04	0.00	0.03	0.01
Cholesteryl Esters in Small LDL	0.09	0.05	-0.04	-0.03	-0.01	0.03	-0.01	-0.03	0.01	0.04	0.01
Free Cholesterol in Small LDL	0.06	0.09	-0.07	-0.04	0.00	0.08	0.00	-0.05	-0.01	0.00	0.03
Triglycerides in Small LDL	0.10	-0.03	0.04	0.03	0.01	0.04	-0.02	-0.04	0.03	0.03	0.02
**HDL Particles, Extra Large**											
Concentration of Very Large HDL Particles	-0.02	0.08	0.09	0.13	-0.07	0.06	-0.02	0.00	0.04	0.04	0.03
Total Lipids in Very Large HDL	-0.04	0.08	0.08	0.14	-0.07	0.04	-0.01	0.01	0.03	0.03	0.02
Phospholipids in Very Large HDL	-0.04	0.07	0.08	0.14	-0.08	0.03	-0.01	0.02	0.03	0.01	0.03
Cholesterol in Very Large HDL	-0.04	0.08	0.07	0.12	-0.07	0.05	-0.02	0.01	0.03	0.03	0.02
Cholesteryl Esters in Very Large HDL	-0.04	0.09	0.07	0.11	-0.07	0.05	-0.02	0.02	0.04	0.03	0.02
Free Cholesterol in Very Large HDL	-0.03	0.07	0.04	0.16	-0.10	0.07	0.01	-0.03	0.00	0.04	0.06
Triglycerides in Very Large HDL	0.07	0.00	0.10	0.12	0.00	0.03	-0.02	-0.03	0.03	0.01	0.07
**HDL Particles, Large**											
Concentration of Large HDL Particles	-0.04	0.08	0.12	0.05	-0.04	-0.01	-0.02	0.04	0.04	0.01	0.03
Total Lipids in Large HDL	-0.04	0.07	0.12	0.05	-0.03	-0.02	-0.01	0.03	0.03	0.01	0.02
Phospholipids in Large HDL	-0.04	0.07	0.13	0.04	-0.03	-0.02	0.00	0.02	0.02	0.00	0.04
Cholesterol in Large HDL	-0.05	0.08	0.10	0.05	-0.04	-0.01	-0.02	0.05	0.05	0.01	0.03
Cholesteryl Esters in Large HDL	-0.05	0.08	0.10	0.04	-0.04	-0.01	-0.03	0.06	0.05	0.01	0.02
Free Cholesterol in Large HDL	-0.04	0.08	0.11	0.07	-0.05	0.00	-0.01	0.02	0.03	0.00	0.05
Triglycerides in Large HDL	0.04	0.01	0.14	0.10	0.02	-0.06	0.01	0.00	-0.02	-0.04	0.11
**HDL Particles, Medium**											
Concentration of Medium HDL Particles	-0.01	0.06	0.15	-0.07	0.02	-0.07	-0.01	0.01	0.00	0.00	0.01
Total Lipids in Medium HDL	0.00	0.05	0.15	-0.07	0.02	-0.08	0.00	0.00	0.00	-0.01	0.01
Phospholipids in Medium HDL	0.00	0.05	0.16	-0.07	0.02	-0.09	0.01	0.00	-0.02	-0.01	0.02
Cholesterol in Medium HDL	-0.02	0.07	0.14	-0.08	0.02	-0.06	-0.01	0.02	0.01	0.00	0.01
Cholesteryl Esters in Medium HDL	-0.02	0.07	0.13	-0.09	0.02	-0.07	-0.01	0.02	0.01	0.00	0.01
Free Cholesterol in Medium HDL	-0.01	0.07	0.15	-0.05	0.01	-0.05	0.00	0.00	0.00	0.00	0.01
Triglycerides in Medium HDL	0.08	-0.03	0.09	0.03	0.04	-0.11	0.03	0.01	-0.03	-0.08	0.04

Table S6: Continued

**HDL Particles, Small**											
Concentration of Small HDL Particles	0.05	0.04	0.05	-0.19	0.06	-0.06	-0.01	-0.01	0.00	-0.01	0.02
Total Lipids in Small HDL	0.06	0.02	0.09	-0.16	0.06	-0.09	0.00	-0.02	-0.02	-0.03	0.02
Phospholipids in Small HDL	0.05	0.02	0.11	-0.15	0.05	-0.10	0.01	-0.01	-0.03	-0.04	0.03
Cholesterol in Small HDL	0.04	0.05	0.05	-0.20	0.07	-0.06	-0.01	-0.01	0.00	-0.01	0.02
Cholesteryl Esters in Small HDL	0.03	0.04	0.03	-0.22	0.06	-0.07	-0.01	0.00	0.00	-0.01	0.03
Free Cholesterol in Small HDL	0.06	0.05	0.10	-0.12	0.07	-0.03	-0.01	-0.04	0.00	-0.01	0.01
Triglycerides in Small HDL	0.09	-0.06	0.03	-0.01	0.05	-0.06	0.01	0.00	0.00	-0.06	0.02
**VLDL Ratios, Extra Large**											
Phospholipids:Tot. Lipids in Extr Large VLDL %	0.03	-0.03	-0.01	0.02	0.07	-0.12	-0.01	0.16	0.07	-0.15	0.49
Cholesterol:Tot. Lipids in Extr Large VLDL %	-0.02	0.05	-0.04	0.00	0.24	-0.09	-0.03	0.01	0.15	-0.05	0.20
Cholesteryl Esters:Tot. Lipids in Extr Large VLDL %	0.00	0.05	-0.05	-0.01	0.20	-0.09	-0.04	0.00	0.16	-0.01	0.32
Free Cholesterol:Tot. Lipids in Extr Large VLDL %	-0.04	0.04	-0.01	0.01	0.25	-0.09	-0.01	0.03	0.10	-0.09	0.24
Triglicerydes:Tot. Lipids in Extr Large VLDL %	0.01	-0.02	0.02	-0.01	-0.21	0.07	0.01	0.02	-0.10	0.09	0.55
**VLDL Ratios, Very Large**											
Phospholipids:Tot. Lipids in VLarge VLDL %	0.06	0.01	-0.07	0.10	-0.01	-0.14	0.00	0.12	0.05	-0.01	0.25
Cholesterol:Tot. Lipids in VLarge VLDL %	-0.04	0.09	-0.06	0.05	0.09	-0.04	0.02	-0.03	0.04	0.06	0.07
Cholesteryl Esters:Tot. Lipids in VLarge VLDL %	-0.04	0.09	-0.07	0.03	0.09	-0.03	0.02	-0.03	0.04	0.05	0.06
Free Cholesterol:Tot. Lipids in VLarge VLDL %	-0.03	0.08	-0.06	0.12	0.05	-0.06	0.03	0.01	0.02	0.09	0.17
Triglicerydes:Tot. Lipids in VLarge VLDL %	0.03	-0.09	0.07	-0.07	-0.09	0.06	-0.02	0.04	-0.03	-0.08	0.16
**VLDL Ratios, Large**											
Phospholipids:Tot. Lipids in Large VLDL %	0.07	-0.04	-0.05	0.04	0.05	-0.13	-0.01	0.07	0.08	-0.12	0.16
Cholesterol:Tot. Lipids in Large VLDL %	-0.01	0.08	-0.08	0.09	0.13	-0.14	0.04	0.03	0.04	0.02	0.04
Cholesteryl Esters:Tot. Lipids in Large VLDL %	-0.02	0.09	-0.06	0.10	0.10	-0.11	0.03	0.03	0.03	0.01	0.04
Free Cholesterol:Tot. Lipids in Large VLDL %	0.04	0.01	-0.10	0.04	0.15	-0.18	0.04	0.03	0.04	0.04	0.13
Triglicerydes:Tot. Lipids in Large VLDL %	-0.03	-0.04	0.09	-0.10	-0.14	0.18	-0.03	-0.05	-0.06	0.04	0.07
**VLDL Ratios, Medium**											
Phospholipids:Tot. Lipids in Medium VLDL %	0.01	0.10	-0.06	0.09	0.02	-0.09	0.04	-0.04	0.00	0.02	0.05
Cholesterol:Tot. Lipids in Medium VLDL %	-0.03	0.11	-0.06	0.06	0.01	-0.05	0.04	0.00	-0.01	0.01	0.04
Cholesteryl Esters:Tot. Lipids in Medium VLDL %	-0.03	0.10	-0.06	0.05	0.00	-0.05	0.03	0.01	-0.01	-0.01	0.06
Free Cholesterol:Tot. Lipids in Medium VLDL %	-0.01	0.11	-0.07	0.07	0.01	-0.06	0.04	0.00	-0.03	0.04	0.03
Triglicerydes:Tot. Lipids in Medium VLDL %	0.02	-0.11	0.06	-0.07	-0.02	0.06	-0.04	0.03	0.00	-0.03	0.03
**VLDL Ratios, Small**											
Phospholipids:Tot. Lipids in Small VLDL %	-0.02	0.11	-0.07	-0.01	-0.03	-0.01	0.02	-0.04	-0.01	0.04	0.03
Cholesterol:Tot. Lipids in Small VLDL %	-0.01	0.09	-0.11	0.03	0.01	-0.08	0.05	-0.04	-0.04	0.03	0.03
Cholesteryl Esters:Tot. Lipids in Small VLDL %	0.00	0.07	-0.12	0.06	0.05	-0.13	0.06	-0.03	-0.04	0.03	0.05
Free Cholesterol:Tot. Lipids in Small VLDL %	-0.02	0.11	-0.07	0.00	-0.04	-0.01	0.02	-0.03	-0.03	0.03	0.02
Triglicerydes:Tot. Lipids in Small VLDL %	0.02	-0.10	0.09	-0.02	0.00	0.05	-0.04	0.06	0.02	-0.04	0.06
**VLDL Ratios, Extra Small**											
Phospholipids:Tot. Lipids in VSmall VLDL %	0.02	-0.08	-0.04	0.12	0.07	0.08	-0.04	-0.01	0.08	0.08	0.25
Cholesterol:Tot. Lipids in VSmall VLDL %	-0.03	0.10	-0.05	0.01	-0.10	-0.05	0.04	0.03	-0.07	-0.04	0.03
Cholesteryl Esters:Tot. Lipids in VSmall VLDL %	-0.03	0.10	-0.04	0.01	-0.11	-0.06	0.04	0.02	-0.07	-0.06	0.04

Table S6: Continued

Free Cholesterol:Tot. Lipids in VSmall VLDL %	0.00	0.07	-0.11	0.01	-0.06	-0.02	0.00	0.07	-0.10	0.10	0.16
Triglicerydes:Tot. Lipids in VSmall VLDL %	0.03	-0.10	0.06	-0.03	0.10	0.03	-0.04	0.00	0.06	0.00	0.05
**ILDL Ratios**											
Phospholipids:Tot. Lipids in IDL %	-0.01	-0.04	-0.03	0.15	0.08	0.04	-0.10	0.11	0.16	0.13	0.26
Cholesterol:Tot. Lipids in IDL %	-0.02	0.09	-0.03	-0.09	-0.13	-0.02	0.05	0.01	-0.10	-0.09	0.07
Cholesteryl Esters:Tot. Lipids in IDL %	-0.01	0.08	0.00	-0.10	-0.15	-0.01	0.05	-0.02	-0.07	-0.18	0.16
Free Cholesterol:Tot. Lipids in IDL %	-0.03	0.06	-0.09	-0.02	0.01	-0.04	0.02	0.08	-0.09	0.18	0.29
Triglicerydes:Tot. Lipids in IDL %	0.03	-0.10	0.05	0.05	0.12	0.01	-0.02	-0.03	0.05	0.03	0.03
**LDL Ratios, Large**											
Phospholipids:Tot. Lipids in Large LDL %	-0.03	-0.01	-0.08	-0.02	-0.08	-0.22	0.12	0.07	-0.18	-0.03	0.15
Cholesterol:Tot. Lipids in Large LDL %	-0.01	0.10	-0.03	-0.06	-0.06	0.13	-0.08	0.06	0.08	-0.03	0.09
Cholesteryl Esters:Tot. Lipids in Large LDL %	0.04	0.03	0.01	-0.08	0.01	0.21	-0.15	0.07	0.17	-0.01	0.20
Free Cholesterol:Tot. Lipids in Large LDL %	-0.05	0.09	-0.04	0.00	-0.09	-0.04	0.05	0.02	-0.06	-0.03	0.07
Triglicerydes:Tot. Lipids in Large LDL %	0.03	-0.09	0.07	0.08	0.10	-0.02	0.02	-0.08	0.01	0.03	0.04
**LDL Ratios, Medium**											
Phospholipids:Tot. Lipids in MediumLDL %	-0.05	0.01	-0.06	-0.04	0.04	0.11	0.03	-0.17	0.07	-0.18	0.35
Cholesterol:Tot. Lipids in MediumLDL %	0.01	0.08	-0.05	-0.06	-0.10	-0.08	-0.01	0.18	-0.09	0.07	0.18
Cholesteryl Esters:Tot. Lipids in MediumLDL %	0.08	-0.02	0.00	-0.01	-0.07	-0.11	-0.03	0.17	-0.07	0.10	0.19
Free Cholesterol:Tot. Lipids in MediumLDL %	-0.07	0.08	-0.05	-0.03	-0.01	0.04	0.02	-0.01	0.00	-0.06	0.05
Triglicerydes:Tot. Lipids in MediumLDL %	0.02	-0.09	0.09	0.09	0.08	0.01	0.00	-0.07	0.05	0.03	0.03
**LDL Ratios, Small**											
Phospholipids:Tot. Lipids in Small LDL %	-0.08	0.01	-0.02	0.08	0.00	0.16	0.00	-0.11	-0.03	-0.06	0.14
Cholesterol:Tot. Lipids in Small LDL %	0.03	0.07	-0.05	-0.11	-0.02	-0.15	0.01	0.14	-0.01	0.02	0.08
Cholesteryl Esters:Tot. Lipids in Small LDL %	0.08	-0.01	0.01	-0.05	-0.02	-0.17	-0.01	0.13	0.02	0.06	0.13
Free Cholesterol:Tot. Lipids in Small LDL %	-0.06	0.08	-0.07	-0.06	0.00	0.04	0.03	0.00	-0.04	-0.06	0.09
Triglicerydes:Tot. Lipids in Small LDL %	0.05	-0.09	0.08	0.05	0.02	-0.01	-0.02	0.00	0.04	0.02	0.02
**HDL Ratios, Very Large**											
Phospholipids:Tot. Lipids in VL HDL %	-0.05	0.04	0.08	0.15	-0.08	-0.03	0.01	0.05	0.02	-0.03	0.17
Cholesterol:Tot. Lipids in VL HDL %	0.02	-0.01	-0.12	-0.17	0.06	0.03	0.00	-0.01	-0.01	0.01	0.09
Cholesteryl Esters:Tot. Lipids in VL HDL %	-0.01	0.07	-0.05	-0.17	0.05	0.04	-0.05	0.07	0.07	0.00	0.14
Free Cholesterol:Tot. Lipids in VL HDL %	0.04	-0.07	-0.12	-0.08	0.03	0.00	0.04	-0.07	-0.07	0.00	0.06
Triglicerydes:Tot. Lipids in VL HDL %	0.08	-0.07	0.00	-0.03	0.07	-0.01	0.00	-0.03	-0.01	-0.01	0.05
**HDL Ratios, Large**											
Phospholipids:Tot. Lipids in Large HDL %	0.02	-0.07	0.04	-0.07	0.06	-0.04	0.08	-0.08	-0.09	-0.05	0.43
Cholesterol:Tot. Lipids in Large HDL %	-0.05	0.08	-0.01	0.03	-0.07	0.02	-0.05	0.11	0.09	0.00	0.13
Cholesteryl Esters:Tot. Lipids in Large HDL %	-0.06	0.08	-0.02	0.01	-0.06	0.01	-0.06	0.14	0.10	0.00	0.13
Free Cholesterol:Tot. Lipids in Large HDL %	-0.01	0.07	0.05	0.12	-0.10	0.04	0.02	-0.03	0.02	-0.03	0.35
Triglicerydes:Tot. Lipids in Large HDL %	0.08	-0.07	-0.01	0.03	0.05	-0.03	0.02	-0.03	-0.05	-0.04	0.11
**HDL Ratios, Medium**											
Phospholipids:Tot. Lipids in Medium HDL %	0.05	-0.09	-0.02	0.06	-0.03	-0.01	0.05	-0.05	-0.10	-0.05	0.11
Cholesterol:Tot. Lipids in Medium HDL %	-0.07	0.08	0.01	-0.08	0.00	0.02	-0.04	0.05	0.06	0.03	0.05
Cholesteryl Esters:Tot. Lipids in Medium HDL %	-0.08	0.07	-0.02	-0.09	0.01	0.01	-0.04	0.07	0.07	0.03	0.05

Table S6: Continued

Free Cholesterol:Tot. Lipids in Medium HDL %	-0.01	0.09	0.11	0.00	-0.01	0.04	-0.01	-0.01	0.01	0.01	0.06
Triglicerydes:Tot. Lipids in Medium HDL %	0.08	-0.07	0.00	0.07	0.02	-0.06	0.03	0.01	-0.03	-0.08	0.04
**HDL Ratios, Small**											
Phospholipids:Tot. Lipids in Small HDL %	-0.02	-0.02	0.14	0.09	-0.06	-0.11	0.04	0.02	-0.07	-0.05	0.10
Cholesterol:Tot. Lipids in Small HDL %	-0.04	0.07	-0.10	-0.11	0.02	0.07	-0.03	0.02	0.05	0.05	0.05
Cholesteryl Esters:Tot. Lipids in Small HDL %	-0.04	0.04	-0.11	-0.15	0.01	0.02	-0.02	0.04	0.04	0.03	0.05
Free Cholesterol:Tot. Lipids in Small HDL %	0.01	0.08	0.05	0.11	0.04	0.16	-0.02	-0.08	0.04	0.04	0.13
Triglicerydes:Tot. Lipids in Small HDL %	0.08	-0.07	-0.01	0.07	0.03	-0.03	0.01	0.01	0.00	-0.05	0.03
